# Influence of Exercise Heat Acclimation Protocol Characteristics on Adaptation Kinetics: A Quantitative Review With Bayesian Meta‐Regressions

**DOI:** 10.1002/cph4.70017

**Published:** 2025-05-29

**Authors:** Peter McDonald, Harry A. Brown, Thomas H. Topham, Monica K. Kelly, William T. Jardine, Amelia Carr, Michael N. Sawka, Andrew P. Woodward, Brad Clark, Julien D. Périard

**Affiliations:** ^1^ Research Institute for Sport and Exercise University of Canberra Bruce Australian Capital Territory Australia; ^2^ Centre for Sport Research, School of Exercise and Nutrition Sciences Deakin University Melbourne Victoria Australia; ^3^ School of Biological Sciences Georgia Institute of Technology Atlanta Georgia USA; ^4^ Faculty of Health University of Canberra Bruce Australian Capital Territory Australia

**Keywords:** heat adaptation, heat stress, humidity, performance, temperature

## Abstract

The integrative influence of heat acclimation (HA) protocol characteristics and approach on adaptation kinetics and exercise capacity/performance in the heat remains unclear. Bayesian multilevel regression models were used to estimate adaptations with the number of exposures, exposure duration, ambient temperature, water vapor pressure, and HA approach (e.g., constant workrate) as predictors. Data from 211 papers were included in meta‐analyses with results presented as posterior means and 90% credible intervals. Mean protocol characteristics were as follows: 8 ± 4 exposures, 90 ± 36 min/exposure, 39.1°C ± 4.8°C, and 2.78 ± 0.83 kPa. HA decreased resting (−5 beats·min^−1^ [−7, −3]) and end‐exercise heart rate (−17 beats·min^−1^ [−19, −14]), resting (−0.19°C [−0.23, −0.14]) and end‐exercise core temperature (−0.43°C [−0.48, −0.36]), and expanded plasma volume (5.6% [3.8, 7.0]). HA also lowered exercise metabolic rate (−87 mL·min^−1^ [−126, −49]), increased whole‐body sweat rate (WBSR) (163 mL·h^−1^ [94, 226]), time to exhaustion (49% [35, 61]) and incremental exercise time (14% [7, 24]), and improved time trial performance (3.1% [1.8, 4.5]). An additional HA exposure increased hemoglobin mass (1.9 g [0.6, 3.2]) and WBSR (9 mL·h^−1^ [1, 17]), and an additional 15 min/exposure further lowered end‐exercise core temperature (−0.04°C [−0.05, −0.03]) and expanded plasma volume (0.4% [0.1, 0.7]). A 5°C increase in ambient temperature further lowered end‐exercise HR (−2 beats·min^−1^ [−3, −1]) and a 1 kPa increase enhanced WBSR (37 mL·h^−1^ [4, 72]). End‐exercise heart rate and core temperature decreased similarly following controlled hyperthermia (−16 beats·min^−1^ [−18, −14]; −0.43°C [−0.48, −0.36]) and constant workrate HA (−17 beats·min^−1^ [−18, −16]; −0.45°C [−0.49, −0.42]). HA protocol characteristics influence the adaptive response and may be manipulated to optimize adaptations. A predictor for estimating HA adaptations based on protocol characteristics is available at: https://www.canberra.edu.au/research/centres/uc‐rise/research/environmental‐physiology/exercise‐heat‐acclimation‐predictor.

## 1 Introduction

Adaptations to support sustained physical activity (i.e., scavenging and hunting) in warm/hot environments were an important selection factor for human evolution (Lieberman 2015) and thus humans have a unique capacity to adapt by natural selection and develop within‐life phenotypic adaptations (Taylor 2014; Horowitz 2014). As with our early ancestors, physical activity or exercise undertaken in the heat is accompanied by elevated thermal and cardiovascular strain relative to when undertaken in cool/temperate conditions, which contributes to impair exercise performance (Sawka et al. 1979; Périard et al. 2011) and increase the risk of exertional heat illness (Laitano et al. 2019; Périard et al. 2022). Climate change has increased the emphasis on interventions that reduce this exacerbated strain to allow for sustaining the health and performance of athletes, occupational workers, and military personnel (Masuda et al. 2024; Borg et al. 2021). Consequently, strategies to offset the negative effects of environmental heat stress on performance are commonly used by athletes, workers, and military personnel, as well as those in occupational settings, with heat acclimation (HA) considered the most important intervention one can adopt prior to training and competing in the heat (Racinais et al. 2015). The HA phenotype induces adaptations that reduce physiological strain and partly restore heat‐mediated decrements in performance (Lorenzo et al. 2010; Keiser et al. 2015). These integrative adaptations are characterized by increased blood volume as well as enhanced sweating and skin blood flow responses that attenuate the rise in body temperature and reduce cardiovascular strain during exercise in the heat (Sawka et al. 2011; Périard et al. 2021). Recent studies also suggest epigenetic‐mediated long‐term molecular protective adaptations (Murray et al. 2022, 2024). Although the benefits of HA have been reviewed, much less focus has been directed toward understanding the coordinated interorgan communication associated with repeated exercise‐heat exposure and quantifying the physiological systems time frame and magnitude (i.e., kinetics) of adaptation. Understanding the kinetics of HA would provide insight into the sequence/hierarchy of specific organ system adaptation stimuli and communication required to achieve heat‐related performance and health benefits.

Most of the physiological adaptations associated with HA are induced within the first week and are generally considered complete after 10–14 days of exposure (Pandolf 1998; Périard et al. 2015). However, several factors influence the kinetics of heat adaptations, including the active or passive nature of the approach, initial acclimation status, frequency, exposure duration, environmental characteristics (i.e., ambient temperature: *T*_a_ and partial pressure of water vapor in air: *P*_a_), and exercise intensity (Périard et al. 2015). Exercise‐HA is generally recommended over passive approaches for developing adaptations that are more specific to the combination of exercise and environmental heat stress athletes will encounter during competition (Périard et al. 2015; Tyler et al. 2016), which is based on the training principle of specificity (Taylor 2014). Moreover, although consecutive days of HA are the approach most commonly used, heat exposures every 2–3 days appear to induce similar adaptations to daily exposures, albeit over a longer time frame (Fein et al. 1975; Duvnjak‐Zaknich et al. 2019).

Longer HA regimens (> 15 exposures) have been reported in previous meta‐analyses to induce more robust adaptations when compared to medium‐term (8–14 exposures) and short‐term protocols (≤ 7 exposures), particularly for sudomotor adaptations (i.e., whole‐body sweat rate: WBSR; Tyler et al. 2016, 2024). However, the limited number of longer HA protocols restricts the formulation of definitive conclusions regarding the influence of total exposures on different adaptations. In addition to protocol length, it has been suggested that 100 min of daily exposure is the optimal dose for HA (Pandolf 1998). However, this suggestion is largely based on data from over 60 years ago (Lind and Bass 1963) in experimental groups with low sample sizes (*n* = 3–4) and low exercise intensities (< 40% peak oxygen uptake: $\dot{V}$O_2peak_). As such, additional empirical evidence to understand the extent to which exposure duration influences exercise‐HA adaptations is required. Moreover, our understanding of how environmental characteristics impact the adaptive response to exercise‐HA is limited. For example, some studies have reported greater improvements in WBSR when acclimating to an elevated *P*_a_ (i.e., humid HA) compared to a low/moderate *P*_a_ (i.e., dry HA; Shvartz et al. 1973; Griefahn 1997; Nielsen et al. 1993, 1997), whereas more recent data reported divergent findings (Tebeck et al. 2020). These data highlight some of the contrasting findings within the literature when acclimating to different time frames, exposure durations, and environmental characteristics, which require further elucidation.

Exercise intensity also appears to influence the adaptative response to exercise‐HA. Houmard et al. (1990) demonstrated that daily moderate‐intensity exercise (~60% $\dot{V}$O_2peak_) for 60 min elicited similar reductions in exercising heart rate (HR) and core temperature (*T*_core_) when compared to 30 min of daily high‐intensity (~75% $\dot{V}$O_2peak_) exercise. However, quantifying exercise intensity during heat training can be challenging for several reasons (Brown et al. 2023). Different HA approaches (e.g., constant workrate, controlled hyperthermia, and controlled HR) result in different absolute (i.e., workrate) and relative (i.e., % $\dot{V}$O_2peak_) exercise intensities, and as such levels of thermal and cardiovascular strain throughout each exposure (Périard et al. 2021). Given the challenges associated with accurately quantifying absolute and relative exercise intensity during HA, determining the adaptive response based on different HA approaches and the nature of the adaptive stimulus may be more insightful.

Therefore, the aim of this review was to systematically assess the exercise‐based HA literature and determine how total number of heat exposures, duration of each exposure, *T*_a_, and humidity (i.e., *P*_a_) influence the kinetics of adaptation. Bayesian multilevel regression models were used to provide estimated changes in resting HR, *T*_core_, blood volume (BV), plasma volume (PV), red cell volume (RCV), and hemoglobin mass (Hb_mass_), as well as end‐exercise HR, *T*_core_ and skin temperature (*T*_sk_), WBSR, local sweat rate (LSR), sweat sodium concentration ([Na^+^]), exercise capacity and performance, based on HA protocol characteristics. We also explored how different HA approaches influenced the adaptative response and the association between initial resting *T*_core_ and HR, as well as $\dot{V}$O_2peak_, on changes in resting measures following HA. Given that there currently exists only general guidance regarding the implementation of HA (Racinais et al. 2015, 2023; Périard et al. 2021; Saunders et al. 2019), the estimated changes in HA adaptations derived from our models have been made publicly available for use by all (https://www.canberra.edu.au/research/centres/uc‐rise/research/environmental‐physiology/exercise‐heat‐acclimation‐predictor). This predictive resource will provide quantitative guidance regarding expected outcomes based on HA approach and protocol characteristics, contributing to abate physiological strain during exercise‐heat stress.

## 2 Methods

This systematic review followed the Preferred Reporting Items for Systematic Reviews and Meta‐Analyses guidelines (Moher et al. 2009; Page et al. 2021). Registration of the review was completed with PROSPERO (CRD42022380238).

### 2.1 Data Sources

A search using databases MEDLINE, Scopus and SPORTDiscus and CINAHL Plus with Full Text was initially completed on July 8, 2022 and updated on December 19, 2024 for all available literature using first‐, second‐, and third‐order search terms. The electronic search was conducted using the following syntax: “acclim*” OR “adapt*” AND “heat*” OR “hot” OR “warm” OR “humid*” OR “dry” OR “desert” OR “tropical” AND “sweat*” OR “thermoreg*” OR “cardiovas*” OR “train*” OR “exer*” OR “work” OR “performance” OR “body temperature” OR “skin temperature” OR “core temperature” OR “rectal temperature” OR “VO2” OR “sweat loss” OR “body mass loss” OR “local sweat rate” OR “heart rate” OR “skin blood flow” OR “oxygen consumption” OR “plasma volume” OR “blood volume” OR “haemoglobin” OR “thermal strain” OR “subjective measures.” The search strategy was narrowed to include only human participants and full‐text journal articles in the English language.

### 2.2 Study Selection

Individual papers (i.e., journal article titles) were imported into Covidence (Covidence systematic review software, Veritas Health Innovation, Melbourne, Australia) and screened for duplicates by one author (PM). Titles and abstracts were screened independently by two authors (PM and HB) to assess the relevance of each paper. Two authors (PM and HB) independently screened full‐text articles. Disagreements were discussed further; if an agreement was not reached, then conflicts were resolved by a third author (JP).

Papers were retained if the following criteria were met: (i) peer‐reviewed studies on healthy human participants (≥ 18 years of age) in the English language, (ii) exercise‐based HA interventions (minimum of three exposures between pre‐ and post‐heat response tests), (iii) environmental conditions, total number of exposures, and exposure duration were reported, or a mean/median could be derived from the data provided. Where additional information was required to confirm inclusion, corresponding authors were emailed, and papers were excluded if authors did not respond within 4 weeks from the initial enquiry.

Papers were excluded if: (i) participants had previously been repeatedly exposed to environmental heat stress (< 4 weeks) or were heat acclimatized, (ii) environmental conditions alternated (e.g., from dry to humid conditions) between days within the HA regimen, (iii) exercise HA was induced with liquid perfused garments, sauna suits, or extra clothing, or (iv) HA was induced via passive heat exposure (e.g., sauna or hot water immersion) in isolation or alongside exercise‐HA.

### 2.3 Outcome Measures

Data were extracted from included papers independently by at least two authors (PM, HB, TT, MK, and WJ) for the following details: sample size, age, height, body mass, and $\dot{V}$O_2peak_ of participants, as well as HA regimen details (i.e., number of exposures, duration of exposures and HA approach) and environmental conditions (*T*_a_ and relative humidity: RH, from which *P*_a_ was calculated). Training status was also extracted based on the McKay et al. (2022) classification framework (Table 1). Outcome variables extracted included *T*_core_, *T*_sk_, HR, metabolic rate during exercise at a constant workrate, WBSR, LSR, sweat [Na^+^], BV, PV, RCV, Hb_mass_, constant workrate exercise capacity (i.e., time to exhaustion), incremental exercise to exhaustion (i.e., $\dot{V}$O_2peak_ test completion time), and performance (i.e., self‐paced exercise time and mean power output). Both resting and end‐exercise *T*_core_ and HR were extracted. Where data were only presented in figures, they were estimated via an online digital extraction tool (Rohatgi 2020). Where data were published in multiple companion papers, as verified by identical participant characteristics, they were only included in the analysis once.

**TABLE 1:** Summary of the participants, heat acclimation protocol characteristics, and risk of bias score for papers that met the inclusion criteria.

Reference	Participant characteristics (*n*/sex, athlete caliber)	Exercise modality and HA approach	HA frequency and duration	HA conditions (*T*_a_ (°C), RH (%), *P*_a_ [kPa])	McMaster risk of bias (/8)
Adams et al. (2017)	17/M, −	Treadmill running and/or cycling; Intermittent exercise and controlled hyperthermia (four intervals sessions at 60%–80% $\dot{V}$O_2peak_ with 5 min rest, six sessions with target *T*_core_: 38.5°C)	10 × ~126 min	40.0, 40, 2.90	5
Alkemade et al. (2022)	11/M, 6/F, Tier 1	Cycling; Controlled hyperthermia (target *T*_core_: 38.5°C)	10 × ~97 min	33.0, 65, 3.30	5
Alkemade et al. (2021)	15/M, 9/F, Tier 1	Cycling; Controlled hyperthermia (target *T*_core_: 38.5°C)	10 × ~97 min	33.0, 65, 3.27	5
Allan (1965)	13, −, −	Circuit training; Controlled hyperthermia (target *T*_core_: 38.3°C)	14 × 180 min	44.0, 45, 4.11	4
Amano et al. (2016)	7/M, Tier 0	Cycling; Constant workrate (2 × 30 min periods at 50% $\dot{V}$O_2peak_ followed by 10 min rest)	7 × 80 min	32.0, 50, 2.38	5
Amano et al. (2015)	9/M, Tier 0	Cycling; Constant workrate (2 × 30 min periods at 50% $\dot{V}$O_2peak_ followed by 10 min rest)	7 × 80 min	32.0, 50, 2.38	6
Amorim et al. (2011)	10/M, 2/F, −	Treadmill walking/running; Constant workrate (5 min warm‐up at 5.6 km·h^−1^, 0% gradient, followed by 2 × 50 min periods at 56% $\dot{V}$O_2peak_ with 15 min between)	10 × ~84 min	42.5, 27, 2.27	6
Aoyagi et al. (1994)	7/M, Tier 1	Treadmill walking; Constant workrate (45%–55% $\dot{V}$O_2peak_)	6 × 60 min	40.0, 30, 2.21	5
	9/M, −	Treadmill walking; Constant workrate (45%–55% $\dot{V}$O_2peak_)	6 × 60 min	40.0, 30, 2.21	
Aoyagi et al. (1995b)	6/M, −	Treadmill walking; Constant workrate (45%–55% $\dot{V}$O_2peak_)	6 × 60 min	40.0, 30, 2.21	6
	9/M, −	Treadmill walking; Constant workrate (45%–55% $\dot{V}$O_2peak_)	6 × 60 min	40.0, 30, 2.21	
Aoyagi et al. (1995a)	8/M, −	Treadmill walking; Constant workrate (45%–55% $\dot{V}$O_2peak_)	6 × 60 min	40.0, 30, 2.21	6
	8/M, −	Treadmill walking; Constant workrate (45%–55% $\dot{V}$O_2peak_)	12 × 60 min	40.0, 30, 2.21	
Aoyagi et al. (1998)	9/M, −	Treadmill walking; Constant workrate (45%–55% $\dot{V}$O_2peak_)	6 × 60 min	40.0, 30, 2.21	6
	6/M, Tier 1	Treadmill walking; Constant workrate (45%–55% $\dot{V}$O_2peak_)	6 × 60 min	40.0, 30, 2.21	
	8/M, −	Treadmill walking; Constant workrate (45%–55% $\dot{V}$O_2peak_)	6 × 60 min	40.0, 30, 2.21	
	8/M, −	Treadmill walking; Constant workrate (45%–55% $\dot{V}$O_2peak_)	12 × 60 min	40.0, 30, 2.21	
Armstrong et al. (1989)	13/M, −	Treadmill running; Intermittent exercise (alternating between running at 65% $\dot{V}$O_2peak_ and standing)	8 × 100 min	41.2, 39, 3.07	5
Armstrong et al. (1990)	5/M, −	Treadmill walking; Constant workrate (5.6 km·h^−1^, 5% gradient)	7 × 90 min	40.1, 25, 1.85	6
Armstrong et al. (1987)	14/M, −	Treadmill running; Intermittent exercise (intermittent bouts of self‐selected running speeds)	8 × 100 min	41.2, 39, 3.07	6
Avellini, Kamon, et al. (1980)	4/M, −	Treadmill walking; Constant workrate (5.6 km·h^−1^, 2% gradient)	10 × 120 min	36.0, 76, 4.52	6
Avellini, Shapiro, et al. (1980)	10/M, −	Treadmill walking; Constant workrate (4.8 km·h^−1^, 0% gradient)	6 × 120 min	49.0, 20, 2.35	6
	9/M, −	Treadmill walking; Constant workrate (4.8 km·h^−1^, 0% gradient)	6 × 120 min	49.0, 20, 2.35	
Avellini et al. (1982)	5/M, −	Treadmill walking; Constant workrate (30% $\dot{V}$O_2peak_)	10 × 120 min	49.0, 21, 2.46	6
Barberio et al. (2015)	8/M, −	Treadmill running; Constant workrate (Speed associated with 4 mmol·L^−1^ blood lactate until volitional exhaustion or an increase in *T*_core_ of 1.5°C)	5 × ~25 min	40.0, 40, 2.95	6
Bass and Jacobson (1965)	12/M, −	Treadmill walking; Constant workrate (6.1 km·h^−1^, 0% gradient: No drug group)	10 × 100 min	48.8, 18, 2.09	4
Best et al. (2014)	7/−, Tier 2	Cycling; Constant workrate (70% $\dot{V}$O_2peak_)	6 × 60 min	35.0, 40, 2.25	6
Borg et al. (2020)	8/M, Tier 2	Cycling; Constant workrate (50% PPO)	5 × 60 min	35.0, 50, 2.81	7
Brade et al. (2013)	10/M, Tier 1	Cycling; Intermittent exercise (8–12 bouts of 3 min at 80% PPO with 1 min passive rest between)	5 × 40 min	35.0, 60, 3.37	7
Brock et al. (1982)	5/F, −	Cycling; Constant workrate (52% $\dot{V}$O_2peak_)	8 × 120 min	40.6, 42, 3.20	6
Buono et al. (1998)	9/M, −	Treadmill walking and Cycling; Constant workrate (4 × 25 min periods at 4.8 km·h^−1^, 3% gradient/75 W with 5 min rest between)	7 × 120 min	35.0, 75, 4.22	5
Buono et al. (2007)	8/M, −	Treadmill walking; Constant workrate (3 × 30 min periods at 28%, 38%, and 47% $\dot{V}$O_2peak_ with 10 min seated rest in cool conditions between)	10 × 90 min	39.3, 47, 3.32	5
Buono et al. (2009)	3/M, 5/F, Tier 0	Treadmill walking and Cycling; Constant workrate (50% of age‐adjusted predicted HR reserve on day 1, then the same absolute work‐rate matched)	10 × 90 min	35.0, 40, 2.25	5
Buono et al. (2018)	3/M, 1/F, −	Treadmill walking; Controlled hyperthermia (target *T*_core_: 37.8°C, 38.1°C, and 38.5°C for 40 min periods each)	7 × 120 min	40.0, 40, 2.95	4
Burk et al. (2012)	21/M, −	Treadmill walking; Constant workrate (2 × 50 min periods at 55% $\dot{V}$O_2peak_ for first 5 days, then 60% O_2peak_ for final 5 days with 10 min rest between)	10 × 110 min	42.0, 18, 1.48	5
Callovini et al. (2021)	12/M, Tier 2	Cycling; Constant workrate (10 min at 30% PPO and 80 min at 50% PPO)	3 × 90 min	35.0, 50, 2.81	3
Campbell et al. (2022)	8/M, 5/F, Tier 1	Cycling; Controlled hyperthermia (target increase *T*_core_: 1.5°C or maximum tolerable workrate)	5 × 60 min	40.0, 52, 3.84	7
Casadio et al. (2016)	2/M, Tier 4	Cycling; Controlled HR (PO for HR corresponding to 60%–70% $\dot{V}$O_2peak_)	5 × 60 min	35.0, 60, 3.37	3
Castle et al. (2011)	8/M, Tier 2	Cycling; Constant workrate (50% $\dot{V}$O_2peak_)	10 × 60 min	33.0, 52, 2.62	5
Chalmers et al. (2016)	9/M, Tier 2	Treadmill running; Intermittent exercise (8 min at RPE 11, 12 min at RPE 13, 8 min at RPE 15, 4 min at RPE 17, and a 2 min warm‐down at RPE 9)	5 × 38 min	35.0, 30, 1.69	6
Chen and Elizondo (1974)	4/M, −	Cycling; Constant workrate (75 W)	9 × 90 min	49.0, 20, 2.35	5
Chen et al. (2013)	7/M, Tier 3	Cycling; Constant workrate (10% below ventilatory threshold in first session 1 and increased by 5% with each session)	5 × ~35 min	38.0, 52, 3.45	8
Cheuvront et al. (2008)	9/M, −	Treadmill walking; Constant workrate (5.6 km·h^−1^, 4% gradient)	9 × ~93 min	45.0, 20, 1.92	6
Chinevere et al. (2008)	8/M, −	Treadmill walking; Constant workrate (5.6 km·h^−1^, 4% gradient)	10 × 100 min	45.0, 20, 1.92	5
Ciuha et al. (2021)	12/M, −	Cycling; Controlled hyperthermia (target *T*_core_: 38.5°C)	10 × 90 min	35.0, 50, 2.81	4
Cleland et al. (1969)	3/F, −	Treadmill walking; Constant workrate (5.6 km·h^−1^, 2.5%–4% gradient)	7 × 52 min	42.2, 29, 2.40	3
Corbett et al. (2022)	16/M, Tier 2	Cycling; Controlled hyperthermia (target *T*_core_: 38.5°C)	8 × 90 min	40.0, 50, 3.69	7
Curley and Hawkins (1983)	6/M, Tier 1	Treadmill walking; Intermittent exercise (alternating between running at 50% $\dot{V}$O_2peak_ and resting)	10 × 155 min	33.3, 75, 3.84	5
Daanen et al. (2011)	15/M, −	Cycling; Constant workrate (45 min at 45% $\dot{V}$O_2peak_ followed by 5 min rest then incremental exercise to volitional exhaustion, with 5 min rest between bouts)	9 × ~103 min	35.0, 29, 1.63	6
Dawson et al. (1989)	5/M, −	Treadmill running; Intermittent exercise (4× bouts of 7.5 s at 15 km·h^−1^ every 30 s, 15% gradient for 15 min with 5 min rest periods)	7 × 80 min	34.3, 65, 3.52	6
Dileo et al. (2016)	10/M, −	Cycling; Constant workrate (2 × 45 min periods at 50% $\dot{V}$O_2peak_ with 15 min rest between)	5 × 105 min	45.0, 20, 1.92	5
Dini et al. (2007)	14/M, Tier 4	Rowing; Constant workrate (50% $\dot{V}$O_2peak_)	10 × 90 min	36.0, 30, 1.78	5
Duvnjak‐Zaknich et al. (2019)	8/M, Tier 2	Cycling; Intermittent exercise (8 × 10 s cycling efforts for 4–7 sets with 2–3 min rest between sets; Intermittent exposure group)	8 × ~41 min	35.0, 60, 3.37	8
	8/M, Tier 2	Cycling; Intermittent exercise (8 × 10 s cycling efforts for 4–7 sets with 2–3 min rest between sets; Daily exposure group)	8 × ~41 min	35.0, 60, 3.37	
Epstein et al. (2010)	4/M, −	Treadmill walking; Constant workrate (5 km·h^−1^, 2% gradient)	12 × 120 min	40.0, 40, 2.95	3
Febbraio et al. (1994)	13/M, −	Cycling; Constant workrate (50% $\dot{V}$O_2peak_)	7 × 90 min	40.0, 20, 1.48	5
Fein et al. (1975)	6/F, −	Treadmill walking; Constant workrate (5.2 km·h^−1^, 2% gradient; Intermittent exposure group)	10 × ~81 min	46.5, 15, 1.66	5
	6/F, −	Treadmill walking; Constant workrate (5.2 km·h^−1^, 2% gradient; Daily exposure group)	10 × ~81 min	46.5, 15, 1.66	
Finberg and Berlyne (1977)	4/M, −	Cycling; Controlled HR (PO to achieve HR of 150–170 beats·min^−1^)	7 × 100 min	50.0, 12, 1.48	5
Flouris et al. (2014)	10/M, −	Cycling; Constant workrate (50% $\dot{V}$O_2peak_)	14 × 90 min	40.0, 20, 1.48	5
Fortney and Senay (1979)	9/F, Tier 1	Cycling; Constant workrate (30% $\dot{V}$O_2peak_)	21 × 90 min	45.0, 30, 2.87	6
Francesconi et al. (1977a)	4/M, −	Treadmill walking; Constant workrate (5.6 km·h^−1^)	7 × 90 min	49.0, 18, 2.11	6
Francesconi et al. (1977b)	4/M, −	Treadmill walking; Constant workrate (5.6 km·h^−1^)	7 × 90 min	49.0, 18, 2.11	6
Francesconi et al. (1978)	4/M, −	Treadmill walking; Constant workrate (5.6 km·h^−1^)	7 × 90 min	49.0, 18, 2.11	6
Frank et al. (2001)	8/M, −	Treadmill walking; Constant workrate (5 min rest followed by 2 × 50 min periods at ~350 W with 10 min rest between)	10 × 115 min	40.0, 40, 2.95	4
Frye and Kamon (1983)	4/M, −	Treadmill walking; Constant workrate (25%–30% $\dot{V}$O_2peak_, humid HA only)	7 × 120 min	37.0, 60, 3.77	5
	4/F, −	Treadmill walking; Constant workrate (25%–30% $\dot{V}$O_2peak_, humid HA only)	7 × 120 min	37.0, 60, 3.77	
Frye and Kamon (1981)	4/M, −	Treadmill walking; Constant workrate (25%–30% $\dot{V}$O_2peak_)	8.5 × 120 min	48.0, 15, 1.67	6
	4/F, −	Treadmill walking; Constant workrate (25%–30% $\dot{V}$O_2peak_)	8.5 × 120 min	48.0, 15, 1.67	
Fujii et al. (2012)	10/M, Tier 0	Cycling; Constant workrate (4 × 20 min bouts of 50% $\dot{V}$O_2peak_ with 10 min rest between)	6 × 110 min	37.0, 50, 3.14	8
Gale et al. (2021)	10/M, Tier 2	Cycling; Intermittent exercise (5 min warm‐up followed by six sets of 5 × 10 s sprints with 30 s active recovery between, and 2 min passive rest between sets)	6 × 40 min	35.0, 50, 2.81	7
Garden et al. (1966)	12/M, −	Treadmill walking; Constant workrate (50 min at 5.6 km·h^−1^, 0% gradient, followed by 10 min rest)	7 × 60 min	36.7, 73, 4.51	4
	13/M, −	Treadmill walking; Constant workrate (2 periods of 50 and 30 min at 5.6 km·h^−1^, 0% gradient, with 10 min rest after each period)	7 × 100 min	36.7, 73, 4.51	
	13/M, −	Treadmill walking; Constant workrate (2 periods of 50 min at 5.6 km·h^−1^, 0% gradient, with 10 min rest after each period)	7 × 120 min	36.7, 73, 4.51	
Garrett et al. (2012)	8/M, Tier 4	Cycling; Controlled hyperthermia (target *T*_core_: 38.5°C)	5 × 90 min	40.0, 60, 4.43	5
Garrett et al. (2019)	10/F, −	Cycling; Controlled hyperthermia (target *T*_core_: 38.5°C)	5 × 90 min	39.5, 60, 4.31	6
Garrett et al. (2009)	10/M, −	Cycling; Controlled hyperthermia (target *T*_core_: 38.5°C)	5 × 90 min	39.5, 60, 4.31	5
Garrett et al. (2014)	9/M, −	Cycling; Controlled hyperthermia (target *T*_core_: 38.5°C; Dehydration group)	5 × 90 min	40.0, 60, 4.43	7
	9/M, −	Cycling; Controlled hyperthermia (target *T*_core_: 38.5°C; Euhydration group)	5 × 90 min	40.0, 60, 4.43	
Gerrett et al. (2021)	5/M, 3/F, Tier 2	Cycling; Controlled hyperthermia (target *T*_core_: 38.5°C; Controlled hyperthermia group)	10 × ~97 min	33.0, 65, 3.27	7
	5/M, 3/F, Tier 2	Cycling; Controlled hyperthermia (target *T*_core_: 38.5°C; Controlled hyperthermia re‐acclimation group)	5 × ~100 min	33.0, 65, 3.27	
	5/M, 3/F, Tier 1	Cycling; Controlled hyperthermia (target *T*_core_: 38.5°C; Immersion group)	10 × ~97 min	33.0, 65, 3.27	
	5/M, 3/F, Tier 1	Cycling; Controlled hyperthermia (target *T*_core_: 38.5°C; Control group)	10 × ~97 min	33.0, 65, 3.27	
Gibson, Mee, Taylor, et al. (2015)	8/M, −	Cycling; Constant workrate (50% $\dot{V}$O_2peak_)	10 × 90 min	40.2, 39, 2.81	7
	8/M, −	Cycling; Controlled hyperthermia (target *T*_core_: 38.5°C)	10 × 90 min	40.2, 39, 2.81	
	8/M, −	Cycling; Controlled hyperthermia (target *T*_core_: 38.5°C on days 1–5, target *T*_core_: 39°C on days 6–10)	10 × 90 min	40.2, 39, 2.81	
Gibson, Mee, Tuttle, et al. (2015)	8/M, −	Treadmill running; Constant workrate (50% $\dot{V}$O_2peak_)	10 × 90 min	40.2, 39, 2.91	5
	8/M, −	Treadmill running; Controlled hyperthermia (target *T*_core_: 38.5°C)	10 × 90 min	40.2, 39, 2.91	
	8/M, −	Treadmill running; Controlled hyperthermia (target *T*_core_: 38.5°C on days 1–5, target *T*_core_: 39°C on days 6–10)	10 × 90 min	40.2, 39, 2.91	
Gibson, Turner, et al. (2015)	8/M, Tier 1	Cycling; Controlled hyperthermia (target *T*_core_: 38.5°C)	10 × 90 min	40.2, 41, 3.06	6
Givoni and Goldman (1973)	24/−, −	Treadmill walking; Constant workrate (5.6 km·h^−1^)	7 × 100 min	49.0, 20, 1.15	4
Greenleaf et al. (1983)	5/M, −	Cycling; Constant workrate (75 W)	8 × 120 min	39.8, 50, 3.65	6
Greenleaf et al. (1985)	6/F, −	Cycling; Constant workrate (~44 $\dot{V}$O_2peak_)	12 × 120 min	40.0, 42, 3.28	6
	4/M, −	Cycling; Constant workrate (~49 $\dot{V}$O_2peak_)	12 × 120 min	40.0, 42, 3.28	
Greenleaf et al. (1981)	5/M, −	Cycling; Constant workrate (~47% $\dot{V}$O_2peak_)	8 × 120 min	39.8, 50, 3.65	6
Griefahn (1997)	6/M, 2F, −	Treadmill walking; Constant workrate (4 × 25 min at 4 km·h^−1^, 0% gradient with 3–5 min rest)	15 × 122 min	37.0, 71, 4.42	6
	6/M, 2/F, −	Treadmill walking; Constant workrate (4 × 25 min at 4 km·h^−1^, 0% gradient with 3–5 min rest)	15 × 122 min	50.0, 15, 1.90	
Guy et al. (2016)	8/M, Tier 2	Cycling; Intermittent exercise (3 min warm‐up followed by 4 × 10 min intervals at 55% $\dot{V}$O_2peak_ with 3 min rest between)	7 × 40 min	35.0, 70, 3.94	8
Hahn et al. (1986)	10/M, −	Cycling; Constant workrate (PO associated with 50% $\dot{V}$O_2peak_)	6 × 60 min	33.0, 67, 3.34	1
Hanson et al. (2022)	6/M, 1/F, Tier 2	Cycling; Constant workrate (58% PPO)	12 × 60 min	30.0, 80, 3.39	5
Haroutounian et al. (2021)	7/M, Tier 2	Cycling; Controlled hyperthermia (target *T*_core_: 38.5°C–38.7°C; Euhydration group)	7 × 90 min	40.0, 35, 2.58	8
	7/M, Tier 2	Cycling; Controlled hyperthermia (target *T*_core_: 38.5°C–38.7°C; Dehydration group)	7 × 90 min	40.0, 35, 2.58	
Heled et al. (2012)	8/M, −	Treadmill walking; Constant workrate (5 km·h^−1^, 2% gradient)	12 × 120 min	40.0, 40, 2.95	5
Hertig and Sargent 2nd (1963)	5/F, −	Treadmill walking; Constant workrate (5.6 km·h^−1^, 0% gradient)	10 × 120 min	45.0, 21, 2.01	3
Hodge et al. (2013)	5/M, 3/F, −	Treadmill walking; Constant workrate (~39% $\dot{V}$O_2peak_)	8 × 90 min	35.3, 40.2, 2.30	5
Hom et al. (2012)	11/M, −	Treadmill walking; Constant workrate (5.6 km·h^−1^, 5% gradient)	11 × 90 min	33.0, 40, 2.01	5
Horstman and Christensen (1982)	6/M, Tier 1	Cycling; Constant workrate (40% $\dot{V}$O_2peak_)	11 × ~91 min	45.0, 14, 1.34	5
	4/F, Tier 1	Cycling; Constant workrate (40% $\dot{V}$O_2peak_)	11 × ~108 min	45.0, 14, 1.34	
Horvath and Shelley (1946)	16/M, −	Marching; Constant workrate (4.7 km·h^−1^)	14 × 60 min	48.9, 37, 4.32	4
Houmard et al. (1990)	9/M, Tier 2	Treadmill walking or running; Constant workrate (40%–50% $\dot{V}$O_2peak_; Low‐intensity group)	7 × 60 min	40.0, 27, 1.99	5
	9/M, Tier 2	Treadmill running; Constant workrate (75% $\dot{V}$O_2peak_; High‐intensity group)	7 × 35 min	40.0, 27, 1.99	
Inoue et al. (1999)	5/M, Tier 1	Cycling; Constant workrate (35% $\dot{V}$O_2peak_)	8 × ~87 min	43.0, 30, 2.59	5
James et al. (2017)	8/M, 1/F, Tier 2	Cycling; Controlled hyperthermia (target *T*_core_: 38.5°C)	5 × 90 min	36.6, 59, 3.62	6
James et al. (2018)	8/M, 1/F, Tier 2	Cycling; Controlled hyperthermia (target *T*_core_: 38.5°C)	5 × 90 min	37.0, 60, 3.77	6
Joy et al. (1964)	21/M, −	Marching; Constant workrate (22.53 km)	12 × 210 min	44.7, 44, 4.11	5
Kaldur et al. (2013)	21/M, Tier 1	Treadmill walking; Constant workrate (2 × 50 min periods at 55% $\dot{V}$O_2peak_ on days 1–5, 60% $\dot{V}$O_2peak_ on days 6–10)	10 × 110 min	42.0, 18, 1.48	5
Kaldur et al. (2014)	21/M, −	Treadmill walking; Constant workrate (2 × 50 min periods at 55% $\dot{V}$O_2peak_ on days 1–5 and 60% $\dot{V}$O_2peak_ on days 6–10, with 10 min rest between)	10 × 110 min	42.0, 18, 1.48	5
Kaufman et al. (1988)	8/M, Tier 1	Cycling; Constant workrate (50% $\dot{V}$O_2peak_)	10 × ~77 min	39.0, 30, 2.10	5
Keiser et al. (2015)	8/M, Tier 2	Cycling; Controlled HR (PO corresponding to HR at 50% $\dot{V}$O_2peak_)	10 × 90 min	38.0, 30, 1.99	7
Kelly et al. (2016)	7/M, Tier 3	Cycling; Intermittent exercise (3 min warm‐up followed by 3 × 5 min periods of alternating intervals of 30 s at 90% and 30% $\dot{V}$O_2peak_ with 3 min active recovery at 50% $\dot{V}$O_2peak_ between sets)	5 × 27 min	38.7, 34, 2.37	8
King et al. (1985)	10/M, −	Cycling; Constant workrate (PO associated with ~55% $\dot{V}$O_2peak_)	8 × 90 min	39.7, 31, 2.25	5
Kirby and Convertino (1986)	10/M, −	Cycling; Controlled hyperthermia (target *T*_core_: ≤ 39.5°C)	10 × 120 min	40.0, 45, 3.32	5
Kirby et al. (2019)	8/F, −	Cycling; Controlled hyperthermia (target *T*_core_: 38.5°C)	9 × ~93 min	40.0, 30, 2.21	6
Kirwan et al. (1987)	8/M, −	Cycling; Constant workrate (PO associated with 50% $\dot{V}$O_2peak_)	8 × 90 min	39.6, 29, 2.11	5
Kissling et al. (2022)	8/M, 5/F, Tier 1	Cycling; Controlled hyperthermia (target *T*_core_ increase: 1.5°C)	5 × 60 min	40.0, 52, 3.84	7
Klous et al. (2021)	10/M, 5/F, Tier 2	Cycling; Controlled hyperthermia (target *T*_core_: 38.5°C; Controlled hyperthermia group)	10 × ~100 min	33.0, 65, 3.27	4
	4/M, 2/F, Tier 2	Cycling; Controlled hyperthermia (target *T*_core_: 38.5°C; Controlled hyperthermia re‐acclimation group)	5 × ~100 min	33.0, 65, 3.27	
Klous et al. (2020)	6/M, 2/F, −	Cycling; Controlled hyperthermia (target *T*_core_: 38.5°C; Controlled hyperthermia group)	10 × ~100 min	33.0, 65, 3.27	4
	4/M, −	Cycling; Controlled hyperthermia (target *T*_core_: 38.5°C; Controlled hyperthermia re‐acclimation group)	5 × ~100 min	33.0, 65, 3.27	
Kotze et al. (1977)	4/M, −	Bench stepping; Constant workrate (~35 W)	10 × 240 min	33.9, 89, 4.71	4
Kristal‐Boneh et al. (1995)	14/M, −	Cycling; Constant workrate (25% $\dot{V}$O_2peak_)	7 × 90 min	42.0, 25, 2.40	5
Kuennen et al. (2011)	8/M, −	Treadmill running; Controlled hyperthermia (50 min of exercise to reach *T*_core_ of 39°C, followed by 50 min with *T*_core_ ≥ 39°C with 10 min rest between)	7 × 110 min	46.5, 20, 2.07	6
Lee and Thake (2017)	7/M, Tier 2	Cycling; Constant workrate (PO associated with 50% $\dot{V}$O_2peak_)	10 × 60 min	40.0, 25, 1.27	6
Lee et al. (2016)	7/M, Tier 2	Cycling; Constant workrate (PO associated with 50% $\dot{V}$O_2peak_)	10 × 60 min	40.0, 25, 1.84	6
Lind and Bass (1963)	4/−, −	Treadmill walking; Constant workrate (5.6 km·h^−1^, 0% gradient; 50 min exposure daily group)	9 × 50 min	49.0, 18, 1.01	3
	3/−, −	Treadmill walking; Constant workrate (5.6 km·h^−1^, 0% gradient; 50 min exposure twice‐daily group)	9 × 100 min	49.0, 18, 1.01	
	3/−, −	Treadmill walking; Constant workrate (5.6 km·h^−1^, 0% gradient; 100 min exposure daily group)	9 × 100 min	49.0, 18, 1.01	
	3/−, −	Treadmill walking; Constant workrate (5.6 km·h^−1^, 0% gradient; 100 min exposure twice‐daily group)	9 × 200 min	49.0, 18, 1.01	
Loeppky (2015)	8/M, −	Cycling; Constant workrate (PO associated with 30% $\dot{V}$O_2peak_)	9 × 100 min	50.0, 16, 1.97	5
Lorenzo et al. (2010)	10/M, 2/F, Tier 2	Cycling; Constant workrate (two periods of 45 min at PO associated with 50% $\dot{V}$O_2peak_, with 10 min rest between)	10 × 100 min	40.0, 30, 2.21	7
Lorenzo and Minson (2010)	10/M, 2/F, Tier 2	Cycling; Constant workrate (two periods of 45 min at PO associated with 50% $\dot{V}$O_2peak_, with 10 min rest between)	10 × 100 min	40.0, 30, 2.21	7
Lundby et al. (2021)	9/M, 2/F, Tier 2	Cycling; Constant workrate (PO corresponding to 45% of PO at 4 mmol·L^−1^ blood lactate)	10 × 50 min	35.1, 62, 3.48	6
Lynch et al. (2024)	9/M, 6/F, Tier 1	Cycling; Constant workrate (120 min at a fixed *H*_prod_ of 275 W·m^2^) and controlled HR (90 min at 75% HR_max_)	7 × ~107 min	37.0, 49, 3.08	6
Magalhães et al. (2010)	9/M, −	Treadmill running; Controlled hyperthermia (target *T*_core_ increase: 1°C)	11 × 60 min	40.0, 45.2, 3.33	6
Mang et al. (2021)	7/M, 6/F, Tier 1	Treadmill walking; Constant workrate (two periods of 45 min at 30%–40% $\dot{V}$O_2peak_, with 10 min rest between)	10 × 100 min	43.0, 40, 3.46	6
Matias et al. (2023)	12/M, 6/F, −	Treadmill running; Intermittent exercise (5 min warm‐up at 5.6 km·h^−1^, 0% gradient, followed by eight periods of 30 s running at 80% APMHR with 90 s active recovery at 50% APMHR)	6 × 31 min	30.0, 50, 2.12	8
McCleave et al. (2020)	11/M, Tier 2	Cycling; Intermittent exercise (varying intervals from 55% to 85% of PPO with 2–7.5 min rest periods between)	12 × 60 min	32.0, 50, 2.38	6
McCleave et al. (2019)	6/M, 3/F, Tier 2	Treadmill running; Constant workrate (45 min at speed associated with 65% $\dot{V}$O_2peak_, followed by 45 min individualized cooldown) and Intermittent exercise (20 min warm‐up, followed by intervals of 80%–100% $\dot{V}$O_2peak_ with 60 s to 3 min rest periods between, and a 20 min cooldown)	9 × 90 min	32.7, 59, 2.91	6
McCleave et al. (2017)	6/M, 3/F, Tier 2	Treadmill running; Constant workrate (45 min at speed associated with 65% $\dot{V}$O_2peak_, followed by 45 min individualized cooldown) and Intermittent exercise (20 min warm‐up, followed by intervals of 80%–100% $\dot{V}$O_2peak_ with 60 s to 3 min rest periods between, and a 20 min cooldown)	9 × 90 min	33.0, 60, 3.02	6
McClung et al. (2008)	7/M, 1/F, −	Treadmill walking; Constant workrate (5.6 km·h^−1^, 4% gradient)	10 × ~87 min	49.0, 20, 2.35	6
McGlynn et al. (2022)	10/F, Tier 0	Cycling; Self‐paced (RPE 15)	14 × 60 min	33.0, 40, 2.01	7
McIntyre et al. (2021)	9/M, −	Treadmill running; Constant workrate (Speed associated with 65% $\dot{V}$O_2peak_)	6 × ~52 min	33.0, 40, 2.01	8
McIntyre et al. (2022)	7/M, −	Treadmill running; Constant workrate (Speed associated with 65% $\dot{V}$O_2peak_)	12 × ~57 min	33.0, 40, 2.01	7
McLellan and Aoyagi (1996)	8/M, −	Treadmill walking; Constant workrate (Speed and gradient associated with 45%–55% $\dot{V}$O_2peak_)	12 × 60 min	40.0, 30, 2.21	5
Mee et al. (2015)	8/M, −	Cycling; Controlled hyperthermia (target *T*_core_: 38.5°C)	10 × 90 min	40.0, 40, 2.95	6
	8/F, −	Cycling; Controlled hyperthermia (target *T*_core_: 38.5°C)	10 × 90 min	40.0, 40, 2.95	
Mee et al. (2016)	5/M, −	Cycling; Controlled hyperthermia (target *T*_core_: 38.5°C)	10 × 90 min	40.0, 40, 2.95	6
	5/F, −	Cycling; Controlled hyperthermia (target *T*_core_: 38.5°C)	10 × 90 min	40.0, 40, 2.95	
Mikkelsen et al. (2019)	12/M, Tier 3	Cycling; Constant workrate (PO associated with 60% $\dot{V}$O_2peak_)	28 × 60 min	37.5, 30, 1.93	7
Mitchell et al. (1976)	4/M, −	Cycling; Constant workrate (PO associated with 40%–50% $\dot{V}$O_2peak_ with 5 min rest periods every 60 min)	10 × 240 min	45.0, 41, 3.93	2
Mitchell et al. (2021)	13/M, Tier 1	Treadmill walking; Constant workrate (5 km·h^−1^, 2% gradient)	8 × 125 min	40.0, 40, 2.95	6
Molloy et al. (2013)	7/M, Tier 1	Treadmill running; Constant workrate (Speed associated with 75% $\dot{V}$O_2peak_; Middle‐aged group)	14 × 30 min	35.0, 35, 1.97	5
	9/M, Tier 1	Treadmill running; Constant workrate (Speed associated with 75% $\dot{V}$O_2peak_; Young group)	14 × 30 min	35.0, 35, 1.97	
Mornas et al. (2023)	8/M, 7/F, Tier 1	Cycling; Constant workrate (PO associated with 1.3–2.5 W·kg^−1^)	13 × 60 min	38.1, 58, 3.86	7
Moss et al. (2020)	13/M, 3/F, −	Cycling; Controlled hyperthermia (target *T*_core_: 38.5°C)	10 × 60 min	40.0, 50, 3.69	4
Naito et al. (2022)	7/M, Tier 1	Cycling; Intermittent exercise (60 × 5 s sprint, 25 s unloaded pedaling and 30 s passive rest, with 10 min rest at the halfway point)	5 × 82 min	36.5, 50, 4.50	7
Neal, Corbett, et al. (2016)	10/M, Tier 2	Cycling; Controlled hyperthermia (target *T*_core_: 38.5°C–38.7°C)	5 × ~85 min	40.0, 50, 3.69	5
Neal, Massey, et al. (2016)	8/M, Tier 1	Cycling; Controlled hyperthermia (target *T*_core_: 38.5°C; Euhydration group)	8 × ~93 min	40.0, 50, 3.69	7
	8/M, Tier 1	Cycling; Controlled hyperthermia (target *T*_core_: 38.5°C; Dehydration group)	8 × ~91 min	40.0, 50, 3.69	
Neufer et al. (1989)	10/M, −	Treadmill walking or running; Constant workrate (2 × 50 min periods at 50% $\dot{V}$O_2peak_ with 10 min rest between)	7 × 110 min	49.0, 20, 2.35	5
Nielsen et al. (1993)	8/M, −	Cycling; Constant workrate (PO associated with 55% $\dot{V}$O_2peak_ to volitional exhaustion)	10.5 × ~64 min	41.0, 13, 0.97	4
Nielsen et al. (1997)	12/M, Tier 2	Cycling; Constant workrate (PO associated with 45% $\dot{V}$O_2peak_ to volitional exhaustion)	10.5 × ~48 min	35.0, 87, 4.89	4
Notley et al. (2018)	12/M, −	Cycling; Controlled hyperthermia (target *T*_core_: 38.5°C)	8 × 90 min	40.0, 37, 2.73	5
Oberholzer et al. (2019)	12/M, Tier 3	Cycling; Constant workrate (PO associated with 60% $\dot{V}$O_2peak_)	28 × 60 min	37.5, 30, 1.93	8
Oöpik et al. (2014)	20/M, −	Treadmill walking; Constant workrate (2 × 50 min periods at 55% $\dot{V}$O_2peak_ on days 1–5 and 60% $\dot{V}$O_2peak_ on days 6–10, with 10 min rest between)	10 × 110 min	42, 18, 1.48	5
Osborne et al. (2021)	8/M, Tier 2	Cycling; Constant workrate (PO associated with 50% PPO)	5 × 60 min	34.9, 53, 2.96	8
O'Toole et al. (1983)	4/F, −	Cycling; Constant workrate (2 × 60 min periods at PO associated with 46% $\dot{V}$O_2peak_, with 5 min rest between)	10 × 125 min	40.0, 50, 4.93	4
Pandolf et al. (1977)	24/M, −	Treadmill walking; Constant workrate (2 × 50 min periods at 4.8 km·h^−1^, 0% gradient, with 10 min rest between)	9 × 110 min	49.0, 20, 2.35	4
Pandolf et al. (1988)	9/M, Tier 1	Treadmill walking; Constant workrate (5.6 km·h^−1^, 5% gradient; Middle‐aged group)	10 × ~119 min	49.0, 20, 2.35	6
	9/M, Tier 1	Treadmill walking; Constant workrate (5.6 km·h^−1^, 5% gradient; Young group)	10 × ~113 min	49.0, 20, 2.35	
Pandolf and Kamon (1974)	4/M, −	Treadmill walking; Constant workrate (Speed associated with 50% $\dot{V}$O_2peak_)	10 × 120 min	49.0, 20, 2.35	5
Parsons et al. (2020)	13/M, Tier 2	Cycling; Controlled hyperthermia (target *T*_core_: 38.5°C)	5 × 90 min	32.1, 70, 3.36	7
Patterson et al. (2004b)	12/M, −	Cycling; Controlled hyperthermia (target *T*_core_: 38.5°C)	16 × 90 min	39.8, 59, 4.32	5
Patterson et al. (2004a)	11/M, −	Cycling; Controlled hyperthermia (target *T*_core_: 38.5°C)	16 × 90 min	40.0, 60, 4.43	5
Patterson et al. (2014)	8/M, −	Cycling; Controlled hyperthermia (target *T*_core_: 38.5°C)	16 × 90 min	39.8, 59, 4.32	5
Périard et al. (2020)	13/M, Tier 2	Cycling; Intermittent exercise (four periods of 5 × 6 s sprint with 24 s passive recovery and 5 min rest between)	5 × 60 min	40.0, 40, 2.95	8
Périard et al. (2023)	14/M, Tier 4	Cycling; Controlled HR (PO corresponding to 65% $\dot{V}$O_2peak_ for 10 min then 60 min at HR corresponding to 65% $\dot{V}$O_2peak_)	5 × 70 min	35.0, 40, 2.25	6
Périard et al. (2024)	10/M, Tier 2	Cycling; Controlled HR (PO corresponding to 65% $\dot{V}$O_2peak_ for 20% of target kJ then HR corresponding to 65% $\dot{V}$O_2peak_ for remaining 80%, for four sessions per week) and; Intermittent exercise (20 min warm‐up, 5 × 30 s max efforts with 4 min 30 s active recovery, then 10 min cool down, for one session per week)	15 × ~68 min	35.5, 59, 3.51	6
Petersen et al. (2010)	6/M, −	Cycling; Intermittent exercise (4–6 bouts of 8 × 20 s sprints with 10 s rest and 4 min rest between sets)	4 × ~38 min	30.0, 61, 2.59	6
Pethick et al. (2019)	13/M, 1/F, Tier 2	Cycling; Controlled hyperthermia (target *T*_core_: 38.5°C; Euhydration group)	5 × 90 min	34.8, 55, 3.04	6
	9/M, 1/F, Tier 2	Cycling; Controlled hyperthermia (target *T*_core_: 38.5°C; Dehydration group)	5 × 90 min	34.8, 55, 3.04	
Philp et al. (2017)	6/M, Tier 3	Cycling; Controlled HR (5 min warm‐up followed by 45 min at PO corresponding to 70% HR reserve)	5 × 50 min	35.0, 56, 3.15	6
Philp et al. (2022)	4/M, 1/F, Tier 3	Rowing or Cycling; Constant workrate (PO corresponding to 45%–55% PPO)	10 × 60 min	34.0, 55, 2.93	5
Pichan et al. (1985)	12/−, −	–; Constant workrate (2 × 50 min exercise periods of 1.675 kJ·h^−1^, with 10 min rest between)	8 × 110 min	45.0, 30, 2.87	5
Piil et al. (2019)	13/M, −	Cycling; Constant workrate (PO associated with 60% $\dot{V}$O_2peak_)	28 × 60 min	39.4, 27, 1.90	5
Poh et al. (2012)	11/M, −	Treadmill walking; Constant workrate (5.6 km·h^−1^, 5% gradient)	10 × 90 min	33.0, 40, 2.01	6
Poirier et al. (2016)	10/M, −	Cycling; Constant workrate (PO associated with 50% $\dot{V}$O_2peak_)	14 × 90 min	40.0, 20, 1.48	5
Poirier et al. (2015)	10/M, −	Cycling; Constant workrate (PO associated with 50% $\dot{V}$O_2peak_)	14 × 90 min	40.0, 20, 1.48	5
Pryor et al. (2019)	9/M, Tier 1	Treadmill running and/or Cycling; Intermittent exercise (four intervals sessions at 60%–80% $\dot{V}$O_2peak_ with 5 min rest between bouts) and controlled hyperthermia (six sessions with target *T*_core_: 38.5°C)	10 × 126 min	40.0, 40, 2.95	7
Pryor et al. (2021)	24/M, Tier 1	Treadmill running and/or Cycling; Intermittent exercise (one interval session with 1–4 min periods of walking, running at 60% and 80% $\dot{V}$O_2peak_ at 1% gradient, with 5 min rest periods) and controlled hyperthermia (three sessions with target *T*_core_: 38.5°C–38.9°C)	4 × ~98 min	40.0, 40, 2.95	7
Pryor et al. (2020)	12/M, −	Treadmill running and/or Cycling; Intermittent exercise (one interval session with 1–4 min periods of walking, running at 60% and 80% $\dot{V}$O_2peak_ at 1% gradient, with 5 min rest periods) and controlled hyperthermia (three sessions with target *T*_core_: 38.5°C–38.9°C)	4 × ~98 min	40.0, 40, 2.95	7
Racinais et al. (2021)	14/M, Tier 4	Cycling; Controlled HR (PO corresponding to 65% $\dot{V}$O_2peak_ for 10 min then 60 min at HR corresponding to 65% $\dot{V}$O_2peak_)	5 × 70 min	35.0, 40, 2.25	7
Racinais et al. (2024)	10/M, Tier 2	Cycling; Controlled HR (PO corresponding to 65% $\dot{V}$O_2peak_ for 20% of target kJ then HR corresponding to 65% $\dot{V}$O_2peak_ for remaining 80%, for four sessions per week) and Intermittent exercise (20 min warm‐up, 5 × 30 s max efforts with 4 min 30 s active recovery, then 10 min cool down, for one session per week)	15 × 68 min	35.5, 59, 3.51	6
Radakovic et al. (2007)	40/M, −	Treadmill walking; Constant workrate (5.5 km·h^−1^)	10 × 150 min	35.0, 40, 2.58	7
Ravanelli et al. (2018)	6/M, 2/F, Tier 0	Treadmill walking; Controlled HR (Speed associated with 70% HR_max_, 3%–5% gradient)	8 × 90 min	38.0, 65, 4.31	6
Ravanelli et al. (2019)	6/M, 2/F, Tier 1	Treadmill walking; Controlled HR (Speed associated with 70% HR_max_, 3%–5% gradient)	6 × 90 min	38.0, 65, 4.31	6
Reeve et al. (2019)	13/M, Tier 2	Cycling; Intermittent exercise (6 min warm‐up at 50% PPO followed by 12 × 1 min at 100% PPO with 1 min of unloaded cycling)	5 × 40 min	35.0, 50, 2.81	7
Regan et al. (1996)	7/M, −	Cycling; Controlled hyperthermia (target *T*_core_ increase: 1°C)	10 × 60 min	38.2, 40, 2.66	6
Relf et al. (2020)	15/M, 4/F, −	Cycling; Controlled hyperthermia (target *T*_core_: 38.5°C or target *T*_core_ increase: 1.5°C)	5 × 120 min	35.0, 50, 2.81	5
Rendell et al. (2017)	8/M, Tier 2	Cycling; Controlled hyperthermia (target *T*_core_: 38.5°C)	8 × 90 min	40.0, 50, 3.69	7
Rivas et al. (2017)	7 M, 5/F, −	Cycling; Controlled hyperthermia (target *T*_core_ increase: 1.5°C)	10 × 90 min	42.0, 28, 2.30	7
Rønnestad et al. (2022)	13/M, Tier 4	Cycling; Constant workrate (45% of PO at 4 mmol·L^−1^ blood lactate, with daily workload increased by 25 W if session RPE < 11 or reduced by 20 W in > 15)	25 × 50 min	35.0, 62, 3.46	7
Rønnestad, Hamarsland, et al. (2021)	11/M, Tier 4	Cycling; Constant workrate (5 min warm‐up followed by 50 min at 45% of PO at 4 mmol·L^−1^ blood lactate, with daily workload increased by 25 W if session RPE ≤ 11)	24 × 55 min	38.0, 65, 4.31	8
Roussey et al. (2021)	9/M, Tier 2	Cycling; Intermittent exercise (two periods 10 × 20 s efforts at RPE 19 with 40 s active rest at RPE 9; High‐intensity group)	5 × ~54 min	39.0, 40, 2.80	7
	8/M, Tier 2	Cycling; Intermittent exercise (Intervals of 33%, 49%, and 64% of PPO: Low‐intensity group)	5 × 70 min	39.0, 40, 2.80	
Rowell et al. (1967)	7/M, −	Treadmill walking; Constant workrate (5.6 km·h^−1^, 0%–5% gradient)	11.5 × ~73 min	48.4, 16, 1.82	4
Saillant et al. (2022)	13/M, Tier 1	Treadmill walking; Constant workrate (5 km·h^−1^, 2% gradient)	8 × 120 min	40.0, 40, 2.95	6
Salgado et al. (2020)	13/M, −	Treadmill walking; Constant workrate (5 km·h^−1^, 2% gradient)	8 × 120 min	40.0, 40, 2.95	6
Sargent 2nd et al. (1965)	4/F, −	Treadmill walking; Constant workrate (5.6 km·h^−1^, 0% gradient)	10 × 120 min	40.0, 49, 4.69	3
Sawka et al. (1985)	13/M, −	Treadmill walking; Constant workrate (Speed associated with 40%–50% $\dot{V}$O_2peak_, 5.5 km·h^−1^, 2%–6% gradient)	9 × 120 min	49.0, 20, 2.35	5
Schleh et al. (2018)	13/M, −	Treadmill walking; Constant workrate (Speed associated with 50% $\dot{V}$O_2peak_; Euhydration group)	3 × 90 min	40.0, 30, 2.21	7
	13/M, −	Treadmill walking; Constant workrate (Speed associated with 50% $\dot{V}$O_2peak_; Dehydration group)	3 × 90 min	40.0, 30, 2.21	
Schmit et al. (2018)	10/M, Tier 2	Treadmill running; Constant workrate (lowest intensity sessions of individual training week, for example, ~11 km·h^−1^; Low‐intensity group)	10 × 45 min	30.0, 50, 2.12	7
	9/M, Tier 2	Treadmill running; Intermittent exercise (highest intensity sessions of individual training week, for example, 8 × 400 m at 19 km·h^−1^; High‐intensity group)	10 × 45 min	30.0, 50, 2.12	
Sekiguchi et al. (2021)	28/M, −	Treadmill running; Controlled hyperthermia (target *T*_core_: 38.5°C–39.75°C)	5 × ~82 min	38.7, 51, 3.53	6
Senay et al. (1976)	4/M, −	Cycling; Constant workrate (PO associated with 40%–50% $\dot{V}$O_2peak_)	10 × 240 min	45.0, 41, 3.93	6
Senay and Kok (1977)	5/M, −	Bench stepping; Constant workrate (30% $\dot{V}$O_2peak_)	8 × 240 min	33.8, 91, 4.79	5
Senay Jr (1978)	6/M, −	Bench stepping; Constant workrate (30% $\dot{V}$O_2peak_)	8 × 240 min	33.8, 93, 26.62	4
Shapiro et al. (1981)	8/M, −	Treadmill walking; Constant workrate (4.8 km·h^−1^, 0% gradient)	10 × 100 min	40.0, 30, 2.21	6
Shapiro et al. (1980)	10/M, −	Treadmill walking; Constant workrate (4.8 km·h^−1^, 0% gradient)	6 × 100 min	49.0, 20, 2.35	6
	9/F, −	Treadmill walking; Constant workrate (4.8 km·h^−1^, 0% gradient)	6 × 100 min	49.0, 20, 2.35	
Shaw et al. (2022)	12/M, −	Cycling; Controlled hyperthermia (target *T*_core_: 38.5°C)	4 × 90 min	40.0, 60, 4.43	5
Shvartz et al. (1973)	6/M, Tier 0	Bench stepping; Constant workrate (15 steps·min^−1^)	6 × ~73 min	39.8, 49, 3.58	6
Shvartz et al. (1977)	7/M, Tier 1	Bench stepping; Constant workrate (41 W; Trained group)	8 × 180 min	39.4, 52, 3.71	5
	7/M, Tier 1	Bench stepping; Constant workrate (41 W; Untrained group)	8 × 180 min	39.4, 52, 3.71	
	7/M, Tier 1	Bench stepping; Constant workrate (41 W; Unfit group)	8 × 180 min	39.4, 52, 3.71	
Shvartz et al. (1979)	5/M, −	Cycling; Constant workrate (PO associated with 50% $\dot{V}$O_2peak_)	8 × 102 min	39.8, 49, 3.58	5
Shvartz et al. (1975)	6/M, −	Bench stepping; Constant workrate (35 W)	8 × 240 min	33.9, 89, 4.71	6
Smith and Havenith (2019)	6/M, Tier 2	Cycling; Controlled hyperthermia (target *T*_core_ increase: 1.4°C)	6 × 90 min	45.0, 20, 1.92	6
Sotiridis et al. (2019)	12/M, Tier 2	Cycling; Controlled hyperthermia (target *T*_core_: 38.5°C)	10 × 90 min	35.0, 57, 3.17	7
Stearns et al. (2013)	10/M, −	Treadmill walking; Constant workrate (5.6 km·h^−1^, 5% gradient)	10 × 90 min	33.0, 40, 2.01	5
Strydom et al. (1976)	20/M, −	Bench stepping; Constant workrate (35 W)	10 × 240 min	33.9, 89, 4.71	6
Sumi et al. (2022)	8/M, Tier 1	Cycling; Constant workrate (Speed associated with 50% $\dot{V}$O_2peak_)	8 × 60 min	30.5, 25, 1.09	7
Sumi et al. (2023)	8/M, −	Cycling; Constant workrate (Speed associated with 50% $\dot{V}$O_2peak_)	8 × 60 min	35.0, 50, 2.81	7
Sunderland et al. (2008)	6/F, −	Running; Intermittent exercise (Loughborough intermittent shuttle test)	4 × ~38 min	30.0, 24, 1.02	6
Takamata et al. (1999)	6/M, −	Cycling; Controlled HR (four periods of 20 min at HR corresponding to 40% $\dot{V}$O_2peak_ with 10 min rest between)	6 × 80 min	36.0, 40, 2.38	6
Takeno et al. (2001)	5/M, −	Cycling; Controlled HR (two periods of 30 min separated by 10 min, starting at PO associated with 60% $\dot{V}$O_2peak_ for 5 min then adjusted to maintain ~140 beats·min^−1^)	10 × 70 min	30.0, 50, 3.70	6
Tamm et al. (2015)	20/M, −	Treadmill walking; Constant workrate (2 × 50 min periods at 55% $\dot{V}$O_2peak_ on days 1–5 and 60% $\dot{V}$O_2peak_ on days 6–10, with 10 min rest between)	10 × 100 min	42.0, 18, 1.48	6
Tebeck et al. (2020)	11/M, Tier 3	Cycling; Controlled hyperthermia (target *T*_core_: 38.5°C; Hot‐dry HA group)	5 × ~96 min	44.5, 18, 1.63	7
	11/M, Tier 3	Cycling; Controlled hyperthermia (target *T*_core_: 38.5°C; Warm‐humid HA group)	5 × ~96 min	32.3, 82, 3.98	
Travers, González‐Alonso, et al. (2020)	8/M, Tier 2	Cycling; Controlled HR (PO corresponding to 65% $\dot{V}$O_2peak_ for 15 min then 75 min at HR corresponding to 65% $\dot{V}$O_2peak_)	10 × 90 min	40.0, 40, 2.95	5
Travers et al. (2021)	7/M, Tier 2	Cycling; Controlled HR (PO corresponding to 65% $\dot{V}$O_2peak_ for 15 min then 75 min at HR corresponding to 65% $\dot{V}$O_2peak_; Euhydration group)	10 × 90 min	40.0, 40, 2.95	7
	7/M, Tier 2	Cycling; Controlled HR (PO corresponding to 65% $\dot{V}$O_2peak_ for 15 min then 75 min at HR corresponding to 65% $\dot{V}$O_2peak_; Dehydration group)	10 × 90 min	40.0, 40, 2.95	
Travers, Nichols, et al. (2020)	8/M, Tier 2	Cycling; Controlled HR (PO corresponding to 65% $\dot{V}$O_2peak_ for 15 min then 75 min at HR corresponding to 65% $\dot{V}$O_2peak_; Euhydration group)	10 × 90 min	40.0, 40, 2.95	6
	8/M, Tier 2	Cycling; Controlled HR (PO corresponding to 65% $\dot{V}$O_2peak_ for 15 min then 75 min at HR corresponding to 65% $\dot{V}$O_2peak_; Dehydration group)	10 × 90 min	40.0, 40, 2.95	
Vesić et al. (2013)	10/M, Tier 1	Treadmill walking; Constant workrate (5.5 km·h^−1^)	10 × 180 min	35.0, 40, 2.25	6
Waldock et al. (2021)	8/M, 3/F, −	Cycling; Controlled hyperthermia (target *T*_core_: 38.5°C)	5 × ~109 min	35.0, 50, 2.81	7
Waldron et al. (2019)	12/M, Tier 2	Cycling; Constant workrate (PO associated with 50% $\dot{V}$O_2peak_)	10 × 60 min	38.0, 30, 1.99	7
Waldron et al. (2020)	10/M, Tier 1	Cycling; Constant workrate (PO associated with 55% $\dot{V}$O_2peak_)	5 × 60 min	36.0, 40, 2.38	8
Watkins et al. (2008)	10/M, −	Cycling; Constant workrate (PO associated with 75% $\dot{V}$O_2peak_)	7 × 30 min	39.5, 27, 1.94	6
Wallett et al. (2022)	13/M, Tier 2	Cycling; Intermittent exercise (four periods of 5 × 6 s sprint with 24 s passive recovery and 5 min rest between)	5 × 60 min	40.0, 40, 2.95	7
Weller et al. (2007)	8/M, Tier 1	Treadmill walking and running; Constant workrate and controlled hyperthermia (60 min at 45% $\dot{V}$O_2peak_ followed by 10 min rest then 40 min at target *T*_core_: 38.5°C; HA group)	10 × 110 min	46.1, 17.9, 1.81	6
	8/M, Tier 1	Treadmill walking and running; Constant workrate and controlled hyperthermia (60 min at 45% $\dot{V}$O_2peak_ then 40 min at target *T*_core_: 38.5°C; Re‐acclimation group)	7 × 110 min	46.1, 18, 1.81	
White et al. (2015)	8/M, Tier 2	Cycling; Constant workrate (2 × 50 min periods at PO associated with 55% of $\dot{V}$O_2peak_, with 10 min rest between)	10 × 110 min	40.0, 20, 1.48	7
Williams et al. (1968)	20/M, −	Bench stepping; Constant workrate (12 steps·min^−1^)	12 × 240 min	33.9, 89, 4.71	3
	20/M, −	Bench stepping; Constant workrate (12 steps·min^−1^)	12 × 240 min	35.6, 89, 5.17	
	20/M, −	Bench stepping; Constant workrate (12 steps·min^−1^)	12 × 240 min	37.2, 89, 5.65	
Williams and Heyns (1969)	20/M, −	Bench stepping; Constant workrate (12 steps·min^−1^)	12 × 240 min	33.9, 89, 4.71	5
	21/M, −	Bench stepping; Constant workrate (12 steps·min^−1^)	12 × 240 min	35.5, 87, 5.20	
Willmott et al. (2016)	7/M, Tier 2	Cycling; Constant workrate (PO associated with 50% $\dot{V}$O_2peak_; Single daily group)	4 × 45 min	35.2, 60, 3.41	6
	7/M, Tier 2	Cycling; Constant workrate (PO associated with 50% $\dot{V}$O_2peak_; Twice‐daily group)	4 × 45 min	35.4, 61, 3.51	
Willmott et al. (2017)	8/M, Tier 2	Cycling; Controlled hyperthermia (target *T*_core_: 38.5°C)	4 × 60 min	44.6, 30, 2.82	6
Wingfield et al. (2016)	10/M, −	Cycling; Intermittent exercise (3 min intervals at 40% and 70% PPO; High‐intensity group)	5 × 30 min	32.0, 60, 2.85	5
	10/M, −	Cycling; Constant workrate (40% of PPO; Low‐intensity group)	5 × 90 min	32.0, 60, 2.85	
Wood and Bass (1960)	4/M, −	Treadmill walking; Constant workrate (4 × 30 min periods daily of walking at 5.6 km·h^−1^, 5% gradient; Study 1)	8 × 120 min	48.9, 18, 2.10	5
	4/M, −	Treadmill walking; Constant workrate (4 × 30 min periods daily of walking at 5.6 km·h^−1^, 5% gradient; Study 2)	5 × 120 min	48.9, 18, 2.10	
Wyndham et al. (1968)	6/M, −	Bench stepping; Constant workrate (12 steps·min^−1^)	17 × 240 min	33.9, 95, 5.03	6
Wyndham et al. (1976)	4/M, −	Cycling; Constant workrate (PO associated with 40%–50% $\dot{V}$O_2peak_)	10 × 240 min	45.0, 42, 4.02	3
Yamada et al. (2007)	10/M, −	Treadmill walking or running; Constant workrate (2 × 50 min periods at 56% $\dot{V}$O_2peak_, with 15 min rest between)	10 × ~96 min	42.5, 28, 2.35	7
Yamazaki (2008)	8/M, Tier 0	Cycling; Constant workrate (4 × 20 min periods at PO associated with 50% $\dot{V}$O_2peak_, with 10 min rest between)	6 × 110 min	36.0, 50, 2.97	6
Yamazaki and Hamasaki (2003)	8/M, −	Cycling; Constant workrate (4 × 20 min periods at PO associated with 50% $\dot{V}$O_2peak_, with 10 min rest between)	6 × 110 min	36.0, 50, 4.44	6
Young et al. (1985)	13/M, −	Treadmill walking; Constant workrate (Speed associated with 40%–50% $\dot{V}$O_2peak_)	9 × 120 min	49.0, 20, 2.35	6
Zappe et al. (1996)	6/M, −	Cycling; Constant workrate (PO associated with 50% $\dot{V}$O_2peak_)	4 × 90 min	30.0, 61, 2.59	6
Zhang and Zhu (2021)	30/M, −	Treadmill running; Constant workrate (5 km·h^−1^)	10 × 90 min	38.0, 40, 2.65	6
Zimmermann et al. (2018)	8/M, −	Cycling; Controlled HR (HR corresponding to 50% $\dot{V}$O_2peak_)	10 × 60 min	34.9, 49, 2.73	7

Abbreviations: APMHR, age predicted maximum heart rate; F, female; HA, heat acclimation; *H*_prod_, metabolic heat production; HR, heart rate; HR_max_, maximum heart rate; M, male; *P*_a_, partial pressure of water vapor in air; PO, power output; PPO, peak power output; RH, relative humidity; RPE, rate of perceived exertion; *T*_a_, ambient temperature; *T*_core_, core temperature; $\dot{V}$O_2peak_, peak oxygen uptake.

Exercise capacity and performance tests were only included if conducted in *T*_a_ ≥ 30°C. To be eligible for inclusion as a valid measure of constant workrate exercise capacity, the test could not have a predetermined fixed duration (e.g., 90 min), but was terminated due to volitional fatigue, or laboratory cutoff points (e.g., internal *T*_core_ > 40°C or 95% HR_max_). Incremental $\dot{V}$O_2peak_ tests in the heat were included in the incremental exercise capacity model if preceded by a period of warming (e.g., constant work exercise in the heat ≥ 20 min). Performance tests (i.e., self‐paced time trials) were converted to percent change in time to allow for comparing between tests by equating a 1% change in power output to a 1% change in running speed or time, and a 0.4% change in time during cycling time trials (Hopkins 2004). For outcome variables assessed during (i.e., exercise metabolic rate, LSR and sweat [Na^+^]) or at the end (i.e., HR, *T*_core_ and *T*_sk_) of exercise, data were only extracted if external workrate was the same for the pre‐ and post‐heat response tests. This was done to avoid potential differences in outcome variable responses between pre‐ and post‐testing being attributable to differences in workrate. Performance tests where workrate differed (e.g., time trials) were included for the assessment of WBSR if no data were reported for a heat response test. When WBSR was reported as sweat loss, it was converted to a rate (mL·h^−1^) using the information provided in the paper. If BV, PV, or RCV was reported in absolute terms (i.e., mL), it was converted to a percentage change from baseline. Where multiple heat response tests were reported throughout an intervention (e.g., mid‐ and post‐intervention), only the first and final heat response test were used. If no defined heat response test was completed pre‐ and post‐intervention and a constant workrate approach to HA was used, the first and last day were compared, with the final day not counting as an exposure day (e.g., 10‐day HA regimen = 9 exposures). If papers had pre‐to‐post heat response tests in multiple environments, the environment most akin to the HA conditions was used. Means and standard deviations (SD) or standard errors (SE) for all outcome variables were extracted. When median and interquartile ranges were reported, data were converted to mean and SD and checked for normality (Shi et al. 2023). Where only confidence intervals were reported, these were converted to SD/SE based on guidelines from the Cochrane Handbook (Higgins et al. 2008). Similarly, if the mean change from baseline was not available, it was calculated from pre‐to‐post test data (Higgins et al. 2008).

### 2.4 Risk of Bias Assessment

The risk of bias for all included studies was independently assessed by two reviewers (PM, HB, TT, MK, and/or WJ) using a modified version of the McMaster critical review form for quantitative studies (Chalmers et al. 2014; Law et al. 1998). The risk of bias for all studies was assessed using the low‐to‐high risk scale, with a scoring system of up to eight points used, with lower scores indicating a higher risk of bias. This critical appraisal tool (Material S1) incorporates six categories from the McMaster tool and includes an additional category that examines the process of controlling and randomization used. Percentage scores for risk of bias are presented as a percentage risk for a low risk of bias; therefore, high scores denote a low risk.

### 2.5 Data Analysis

To assess the relationship between changes in outcome variables and HA protocol characteristics, individual multilevel meta‐regression models were applied using “brms” package (Bürkner 2017) via R software (R Core Team 2009) in RStudio (version 4.2.1). A meta‐regression model was conducted for each outcome variable, fitted with the given outcome variable as the response variable, with *T*_a_ (in °C), *P*_a_ (in kPa), total number of exposures (in days), duration per exposure (in min), and HA approach (constant workrate, controlled hyperthermia, controlled HR or intermittent exercise) as the predictors. For exercise metabolic rate, RCV, Hb_mass_, LSR, sweat [Na^+^], and exercise capacity models, HA approach was not entered as a predictor variable due to a lack of papers/observations and difference in approaches used. Smooth terms (e.g., splines) were initially included in the models but these did not converge, likely due to a combination of data limitations and model complexity. As such, we examined the linear relationship between each outcome variable and an additional day of exposure, a 15‐min increase in duration per exposure, and a 5°C and 1 kPa increase in *T*_a_ and *P*_a_ during HA, respectively. The specified changes in predictor variables were chosen based on the range each predictor can be practically manipulated during HA. The response variables were defined as the mean difference and SE observed between pre‐ and post‐observations. Exposure days, exposure duration, *T*_a_ and *P*_a_ were entered as continuous variables, while HA approach was entered as a categorical variable. To assess the convergence of all models, Rhat statistics, the effective sample size, along with visual inspection of the Markov chain Monte Carlo (Vehtari et al. 2021) were used. Figures were developed using the “ggplot2” package (Wickham 2016). Additional analyses were conducted to determine the linear relationship between aerobic fitness (i.e., $\dot{V}$O_2peak_) and initial baseline value on changes in resting HR and *T*_core_. A meta‐regression model was fitted with resting HR and *T*_core_ as the response variable, and either initial $\dot{V}$O_2peak_ or baseline values as the predictor variable. These models were conducted separately due to the potential collinearity between variables. We examined the influence of a 15 beats·min^−1^ and a 0.5°C higher baseline for resting HR and resting *T*_core_, alongside a 15 mL·kg^−1^·min^−1^ lower $\dot{V}$O_2peak_, respectively.

Descriptive statistics are presented as mean ± SD, with data from models presented as posterior means with 90% credible intervals (CrI). In addition, 90% prediction intervals (PrI) were used in figures to estimate where data from future studies may fall (Material S2, Figures 2, 3, 4, 5, 6, 7, 8, 9, 10; Borg et al. 2023). The probability of direction (Pd) was defined as the proportion of posterior draws on either side of zero and is presented as a percentage (%). Priors for all outcome variables were intended to be weakly informative and were set as normally distributed, with the mean at zero and the SD as: resting *T*_core_ (0.4°C), end‐exercise *T*_core_ and *T*_sk_ (1°C), resting (10 beats·min^−1^) and end‐exercise HR (40 beats·min^−1^), PV (15%), BV (15%), RCV (5%), Hb_mass_ (50 g), exercise metabolic rate (200 mL·min^−1^), WBSR (400 mL·h^−1^), upper back and forearm LSR (0.4 mg·cm^−2^·min^−1^), sweat [Na^+^] (50 mmol·L^−1^), incremental exercise test (30%), time to exhaustion test (30%), and self‐paced time‐trial performance (10%). Scaled priors were applied to all continuous predictor variables (Gelman 2006).

## 3 Results

**FIGURE 1:**
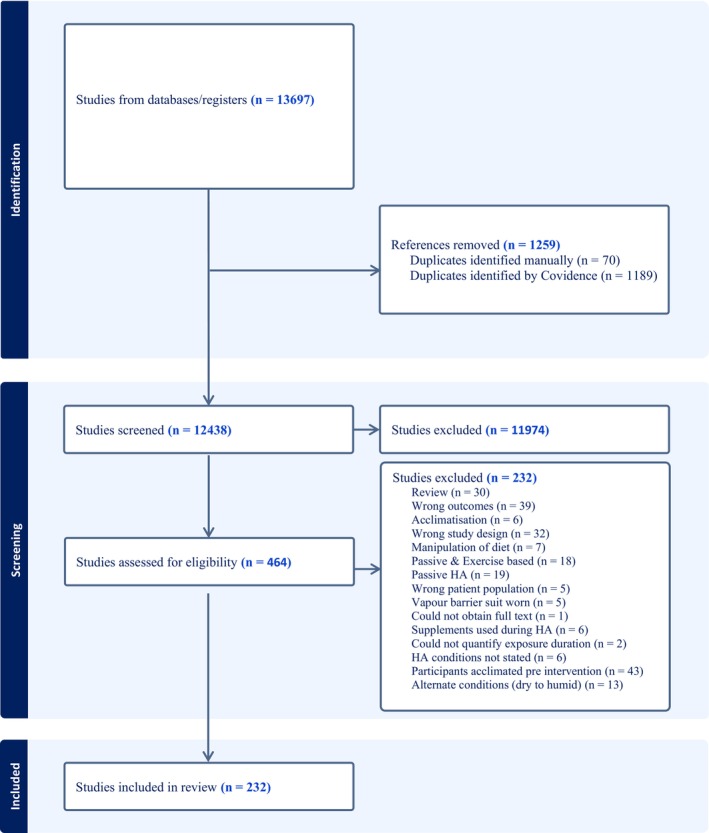
PRISMA flow diagram outlining the identification, screening, and inclusion of papers in this review.

The processes used to assess the 232 papers included in this meta‐analysis are shown in Figure 1. Papers were most commonly excluded due to participants being acclimated or acclimatized pre‐intervention, absence of relevant outcomes for the current analyses, review papers, wrong study design, using passive HA in isolation or alongside exercise‐HA, or alternating environmental conditions. Of the 232 papers (Table 1), data from a total of 2587 participants were extracted, of which a minimum of 239 participants were replicated in more than one paper. Of the total participant sample, 229 were female (8.9%), with four papers (*n* = 56) not directly specifying sex (Lind and Bass 1963; Best et al. 2014; Givoni and Goldman 1973; Pichan et al. 1985). The weighted means (±SD; weighting based on individual study sample size) for participant characteristics were as follows: age (25 ± 5 years), height (1.75 ± 0.06 m), body mass (73.8 ± 7.6 kg), and $\dot{V}$O_2max_ (52.6 ± 7.7 mL·kg^−1^·min^−1^). The most common exercise‐based HA approach was constant workrate (139 papers), followed by controlled hyperthermia (46 papers), intermittent exercise (19 papers), controlled HR (13 papers), self‐paced exercise (1 papers), a combination of intermittent and controlled hyperthermia (4 papers), constant workrate and intermittent exercise (2 papers), controlled HR and intermittent exercise (2 papers), constant workrate and controlled hyperthermia (1 paper), and constant workrate and controlled HR (1 paper), with four studies comparing constant workrate HA to controlled hyperthermia (2 papers) or intermittent exercise (2 papers). Of the 232 papers, SD, SE, or confidence intervals were not provided for 21 (Lind and Bass 1963; Givoni and Goldman 1973; Avellini, Shapiro, et al. 1980; Bass and Jacobson 1965; Borg et al. 2020; Garden et al. 1966; Hertig and Sargent 2nd 1963; Horvath and Shelley 1946; O'Toole et al. 1983; Pandolf and Kamon 1974; Radakovic et al. 2007; Sargent 2nd et al. 1965; Shvartz et al. 1975; Vesić et al. 2013; Williams et al. 1968; Williams and Heyns 1969; Buono et al. 2018; Senay and Kok 1977; Frye and Kamon 1983; Joy et al. 1964; Allan 1965), with data from Borg et al. (2020) captured in a companion paper (Osborne et al. 2021). Thus, a total of 211 papers had at least one outcome variable included in these meta‐analyses (Material S3).

### 3.1 Model Performance

The models for resting and end‐exercise HR, PV, resting and end‐exercise *T*_core_, end‐exercise *T*_sk_, WBSR and time trial performance with exposure days, *T*_a_, *P*_a_, exposure duration and HA approach as predictors, along with the models for exercise metabolic rate, BV, RCV, Hb_mass_, upper back and forearm LSR, sweat [Na^+^], time to exhaustion and incremental exercise with exposure days, *T*_a_, *P*_a_, and exposure duration as predictors, all successfully converged (Rhat = 1.0). The additional models for resting *T*_core_ and HR with pre‐HA (i.e., baseline) resting *T*_core_ and HR, as well as $\dot{V}$O_2peak_ as predictors also successfully converged (Rhat = 1.0).

### 3.2 Risk of Bias

Overall, included papers provided a low certainty of risk for an appropriate study purpose (96%) and conclusion (94%), reliable and valid outcome measures (96%), statistical analysis (88%), and implementation of a thoroughly detailed intervention (96%). In contrast, studies commonly scored a high certainty for risk of bias due to the lack of control groups (60%), randomization of participants (19%), and not justifying sample size (20%). A large number of papers included are at a high risk of bias due to the absence of a control group, which is in line with previous meta‐analyses (Tyler et al. 2016, 2024; Chalmers et al. 2014). Failing to justify sample size is a common occurrence for studies scoring a high risk of bias. However, due to factors such as population limitations (e.g., elite athletes), participant availability (e.g., team sports), and protocol time frame (i.e., weeks), HA studies are often limited in how many participants they include.

#### 3.2.1 Cardiovascular Responses

##### 3.2.1.1 Resting Heart Rate

**FIGURE 2:**
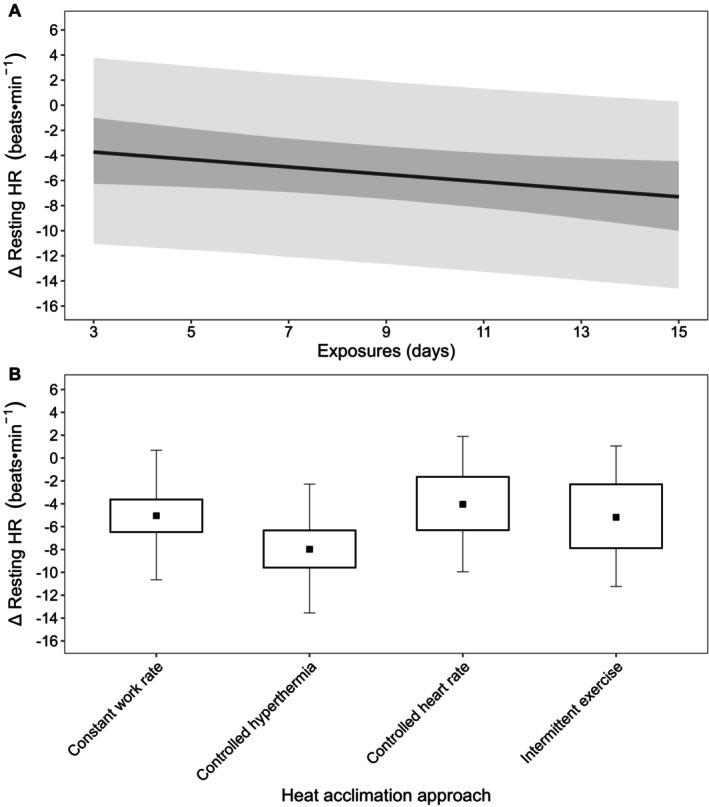
(A) Estimated change (Δ) in resting heart rate (HR) across days of exposure based on the global means of the predictor variables (*T*_a_: 38.5°C, *P*_a_: 2.92 kPa, 84 min per exposure). The inner dark gray band represents the 90% CrI and the outer light gray band denotes the 90% PrI. (B) Estimated change in resting HR based on the heat acclimation approach with 90% CrI (box) and 90% PrI (error bars). Estimates are based on the global means of the predictor variables for a mean duration of 8.1 days.

The resting HR model was based on 58 observations (*n* = 556) across 47 papers, with mean (SD) protocol characteristics of: 38.5°C ± 3.2°C, 2.92 ± 0.73 kPa, 8.1 ± 3.2 days, and 84 ± 31 min per exposure. Using these global means and based on a weighted average HA approach, a change in resting HR of −5.3 beats·min^−1^ (−7.2, −3.0; Pd > 99%) was estimated, with a change of −0.3 beats·min^−1^ (−0.6, 0.0; Pd = 96%) per additional day of exposure (Figure 2A). The estimated change in resting HR was −0.1 beats·min^−1^ (−0.6, 0.3; Pd = 67%) for a 15‐min increase in exposure duration, 0.3 beats·min^−1^ (−1.2, 1.8; Pd = 64%) for a 5°C increase in *T*_a_, and 0.1 beats·min^−1^ (−1.2, 1.4; Pd = 54%) for a 1 kPa increase in *P*_a_. The estimated change in resting HR was −5.0 beats·min^−1^ (−6.5, −3.6; Pd > 99%; *n* = 203) for constant workrate HA, −8.0 beats·min^−1^ (−9.6, −6.3; Pd > 99%; *n* = 199) for controlled hyperthermia HA, −4.1 beats·min^−1^ (−6.3, −1.6; Pd > 99%; *n* = 46) for controlled HR HA, and −5.2 beats·min^−1^ (−7.9, −2.3; Pd > 99%; *n* = 54) for intermittent exercise‐HA (Figure 2B).

The change in resting HR model accounting for pre‐HA resting HR (global mean: 72 ± 10 beats·min^−1^) was based on 56 observations (*n* = 534) across 45 papers. For a 15 beats·min^−1^ higher pre‐HA resting HR, a further change of −2.5 beats·min^−1^ (−3.9, −1.1; Pd > 99%) was estimated. The change in resting HR model accounting for pre‐HA $\dot{V}$O_2peak_ (global mean: 51 ± 6 mL·kg^−1^·min^−1^) was based on 52 observations (*n* = 503) across 41 papers. For a 15 mL·kg^−1^·min^−1^ lower pre‐HA $\dot{V}$O_2peak_, a further change of −0.6 beats·min^−1^ (−5.0, 3.8; Pd = 59%) was estimated.

##### 3.2.1.2 End‐Exercise Heart Rate

**FIGURE 3:**
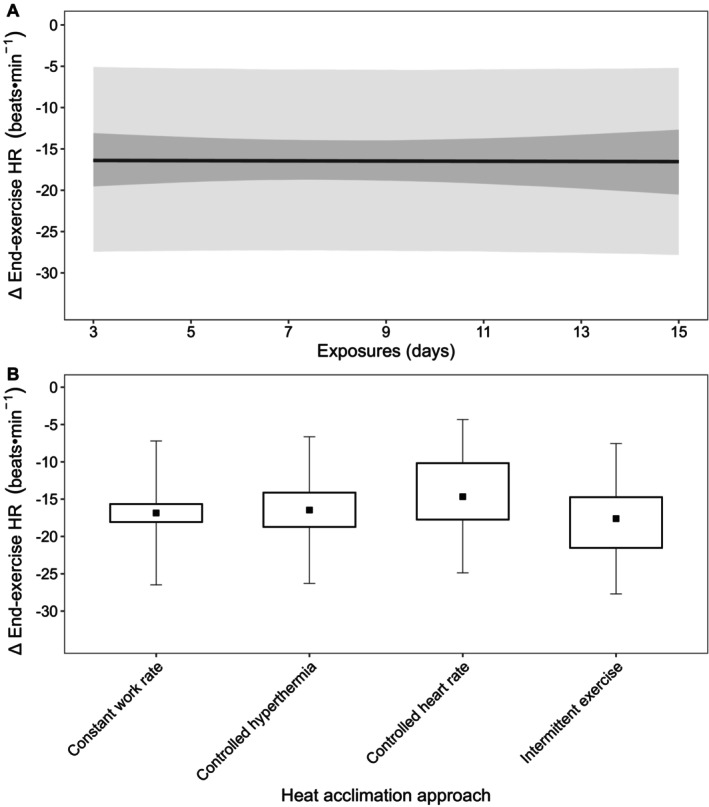
(A) Estimated change (Δ) in end‐exercise heart rate (HR) across days of exposure based on the global means of the predictor variables (*T*_a_: 40.0°C, *P*_a_: 2.66 kPa, 96 min per exposure). The inner dark gray band represents the 90% CrI and the outer light gray band denotes the 90% PrI. (B) Estimated change in end‐exercise HR based on the heat acclimation approach with 90% CrI (box) and 90% PrI (error bars). Estimates are based on the global means of the predictor variables for a mean duration of 8.0 days.

The end‐exercise HR model was based on 118 observations (*n* = 1089) across 101 papers, with mean protocol characteristics of: 40.0°C ± 4.9°C, 2.66 ± 0.84 kPa, 8.0 ± 2.5 days, and 96 ± 35 min per exposure. Using these global means and based on a weighted average HA approach, a change in end‐exercise HR of −16.5 beats·min^−1^ (−18.7, −14.0; Pd > 99%) was estimated, with a change of 0.0 beats·min^−1^ (−0.4, 0.4; Pd = 52%) per additional day of exposure (Figure 3A). The estimated change in end‐exercise HR was −1.1 beats·min^−1^ (−1.6, −0.5; Pd > 99%) for a 15‐min increase in exposure duration, −1.8 beats·min^−1^ (−3.0, −0.7; Pd = 99%) for a 5°C increase in *T*_a_, and 0.7 beats·min^−1^ (−0.8, 2.2; Pd = 79%) for a 1 kPa increase in *P*_a_. The estimated change in end‐exercise HR was −16.8 beats·min^−1^ (−18.1, −15.7; Pd > 99%; *n* = 755) for constant workrate HA, −16.5 beats·min^−1^ (−18.7, −14.1; Pd > 99%; *n* = 174) for controlled hyperthermia HA, −15.2 beats·min^−1^ (−17.7, −10.2; Pd > 99%; *n* = 39) for controlled HR HA, and −17.3 beats·min^−1^ (−21.5, −14.7; Pd > 99%; *n* = 49) for intermittent exercise‐HA (Figure 3B).

##### 3.2.1.3 Exercise Metabolic Rate

The exercise metabolic rate model was based on 26 observations (*n* = 244) across 20 papers, with mean protocol characteristics of: 40.0°C ± 4.1°C, 2.90 ± 0.86 kPa, 7.5 ± 2.0 days, and 90 ± 42 min per exposure. Using these global means, a change in exercise metabolic rate of −87 mL·min^−1^ (−126, −49; Pd > 99%) was estimated, with a change of 5 mL·min^−1^ (−8, 17; Pd = 73%) per additional day of exposure. The estimated change in exercise metabolic rate was −3 mL·min^−1^ (−17, 12; Pd = 62%) for a 15‐min increase in exposure duration, −28 mL·min^−1^ (−64, 11; Pd = 89%) for a 5°C increase in *T*_a_, and –20 mL·min^−1^ (−52, 15; Pd = 83%) for a 1 kPa increase in *P*_a_.

#### 3.2.2 Hematological Responses

##### 3.2.2.1 Blood Volume

The BV model was based on 31 observations (*n* = 258) across 26 papers, with mean protocol characteristics of: 38.4°C ± 3.6°C, 2.96 ± 0.71 kPa, 10.1 ± 5.7 days, and 80 ± 22 min per exposure. Using these global means and based on a weighted average HA approach, a change in BV of 2.9% (0.9, 4.7; Pd = 98%) was estimated, with a change of 0.0% (−0.2, 0.2; Pd = 60%) per additional day of exposure. The estimated change in BV was 0.7% (−0.2, 1.5; Pd = 91%) for a 15‐min increase in exposure duration, −0.8% (−2.6, 1.0; Pd = 77%) for a 5°C increase in *T*_a_, and −0.1% (−1.6, 1.5; Pd = 53%) for a 1 kPa increase in *P*_a_. The estimated change in BV was 3.7% (2.2, 5.5; Pd > 99%; *n* = 111) for constant workrate HA, 3.4% (1.5, 5.4; Pd > 99%; *n* = 50) for controlled hyperthermia HA, 2.7% (0.6, 4.3; Pd = 98%; *n* = 71) for controlled HR HA, and 2.6% (−0.8, 4.8; Pd = 92%; *n* = 16) for intermittent exercise‐HA.

##### 3.2.2.2 Plasma Volume

**FIGURE 4:**
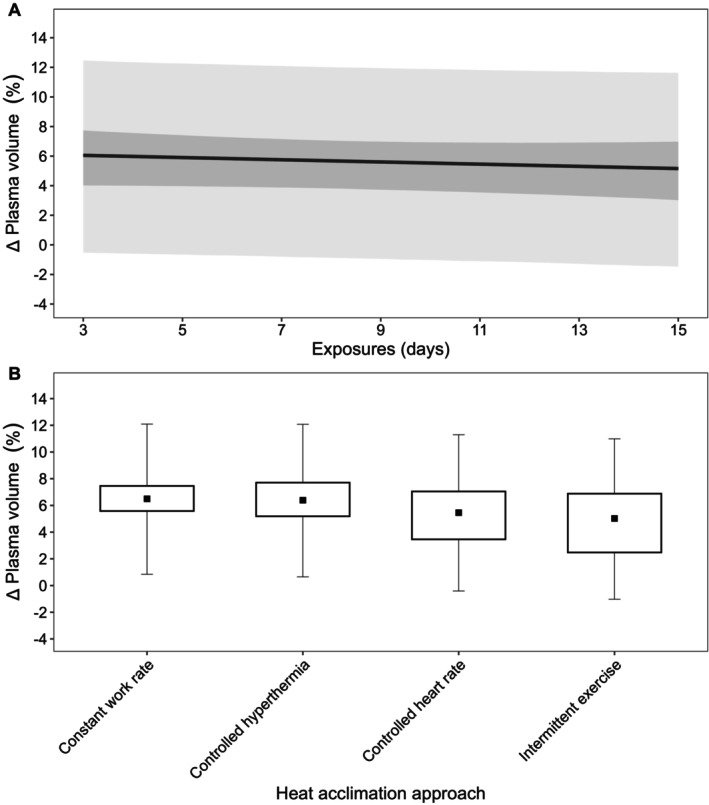
(A) Estimated change (Δ) in plasma volume (PV) across days of exposure based on the global means of the predictor variables (*T*_a_: 38.5°C, *P*_a_: 2.90 kPa, 86 min per exposure). The inner dark gray band represents the 90% CrI and the outer light gray band denotes the 90% PrI. (B) Estimated change in PV based on the heat acclimation approach with 90% CrI (box) and 90% PrI (error bars). Estimates are based on the global means of the predictor variables for a mean duration of 8.6 days.

The PV model was based on 94 observations (*n* = 859) across 82 papers, with mean protocol characteristics of: 38.5°C ± 4.2°C, 2.90 ± 0.88 kPa, 8.6 ± 4.5 days, and 86 ± 37 min per exposure. Using these global means and based on a weighted average HA approach, a change in PV of 5.6% (3.8, 7.0; Pd > 99%) was estimated, with a change of −0.1% (−0.2, 0.1; Pd = 77%) per additional day of exposure (Figure 4A). The estimated change in PV was 0.4% (0.1, 0.7; Pd = 98%) for a 15‐min increase in exposure duration, 0.1% (−0.8, 1.0; Pd = 57%) for a 5°C increase in *T*_a_, and −0.3% (−1.2, 0.7; Pd = 68%) for a 1 kPa increase in *P*_a_. The estimated change in PV was 6.5% (5.6, 7.5; Pd > 99%; *n* = 430) for constant workrate HA, 6.4% (5.2, 7.7; Pd > 99%; *n* = 263) for controlled hyperthermia HA, 5.6% (3.5, 7.0; Pd > 99%; *n* = 63) for controlled HR HA, and 5.2% (2.5, 6.9; Pd > 99%; *n* = 63) for intermittent exercise‐HA (Figure 4B).

##### 3.2.2.3 Red Cell Volume

The RCV model was based on 23 observations (*n* = 201) across 19 papers, with mean protocol characteristics of: 38.8°C ± 3.9°C, 3.13 ± 0.86 kPa, 11.5 ± 6.8 days, and 82 ± 21 min per exposure. Using these global means, a change in RCV of 1.3% (0.4, 2.2; Pd = 99%) was estimated, with a change of 0.0% (0.0, 0.2; Pd = 85%) per additional day of exposure. The estimated change in RCV was 0.0% (−0.4, 0.5; Pd = 53%) for a 15‐min increase in exposure duration, −0.5% (−1.6, 0.6; Pd = 77%) for a 5°C increase in *T*_a_, and 0.7% (−0.2, 1.6; Pd = 91%) for a 1 kPa increase in *P*_a_.

##### 3.2.2.4 Hemoglobin Mass

The Hb_mass_ model was based on 13 observations (*n* = 139) across 12 papers, with mean protocol characteristics of: 37.0° ± 4.6°C, 2.92 ± 0.76 kPa, 12.5 ± 8.1 days, and 71 ± 18 min per exposure. Using these global means, a change in Hb_mass_ of −3.6 g (−12.1, 6.0; Pd = 75%) was estimated, with a change of 1.9 g (0.6, 3.2; Pd = 99%) per additional day of exposure. The estimated change in Hb_mass_ was −0.9 (−5.4, 3.8; Pd = 62%) for a 15‐min increase in exposure duration, −1.7 g (−11.1, 7.4; Pd = 62%) for a 5°C increase in *T*_a_, and 2.7 g (−7.0, 12.9; Pd = 68%) for a 1 kPa increase in *P*_a_.

#### 3.2.3 Thermal Responses

##### 3.2.3.1 Resting Core Temperature

**FIGURE 5:**
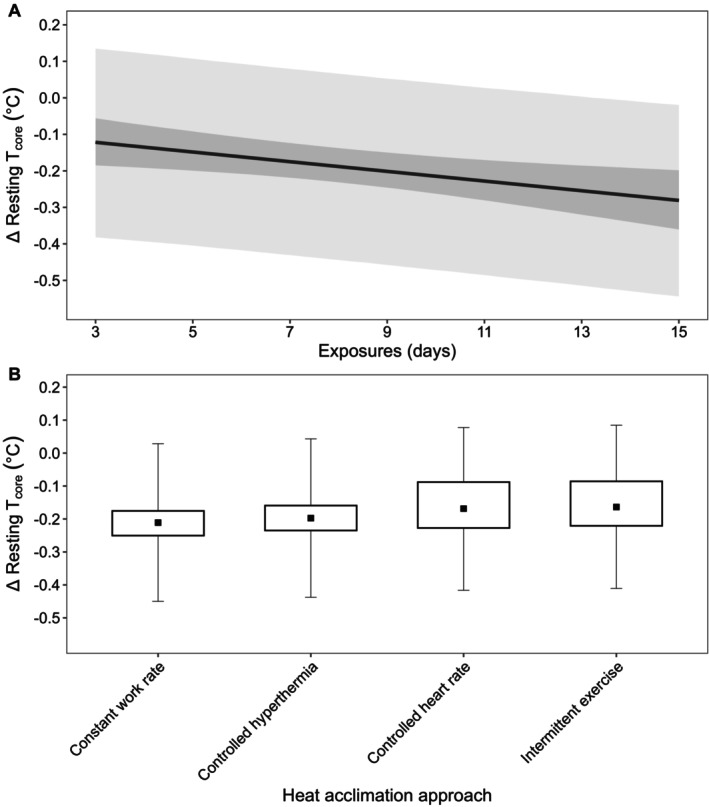
(A) Estimated change (Δ) in resting core temperature (*T*_core_) across days of exposure based on the global means of the predictor variables (*T*_a_: 38.0°C, *P*_a_: 2.94 kPa, 87 min per exposure). The inner dark gray band represents the 90% CrI and the outer light gray band denotes the 90% PrI. (B) Estimated change in resting *T*_core_ based on the heat acclimation approach with 90% CrI (box) and 90% PrI (error bars). Estimates are based on the global means of the predictor variables for a mean duration of 7.9 days.

The resting *T*_core_ model was based on 103 observations (*n* = 1014) across 82 papers, with mean protocol characteristics of: 38.0°C ± 3.8°C, 2.94 ± 0.80 kPa, 7.9 ± 2.8 days, and 87 ± 36 min per exposure. Using these global means and based on a weighted average HA approach, a change in resting *T*_core_ of −0.19°C (−0.23, −0.14; Pd > 99%) was estimated, with a change of −0.01°C (−0.02, 0.00; Pd = 99%) per additional day of exposure (Figure 5A). The estimated change in resting *T*_core_ was −0.01°C (−0.02, 0.01; Pd = 82%) for a 15‐min increase in exposure duration, −0.01°C (−0.05, 0.03; Pd = 67%) for a 5°C increase in *T*_a_, and −0.01°C (−0.05, 0.02; Pd = 71%) for a 1 kPa increase in *P*_a_. The estimated change in resting *T*_core_ was −0.21°C (−0.25, −0.18; Pd > 99%; *n* = 426) for constant workrate HA, −0.20°C (−0.23, −0.16; Pd > 99%; *n* = 383) for controlled hyperthermia HA, −0.18°C (−0.23, −0.09; Pd > 99%; *n* = 54) for controlled HR HA, and −0.17°C (−0.22, −0.09; Pd > 99%; *n* = 86) for intermittent exercise‐HA (Figure 5B).

The change in resting *T*_core_ model accounting for pre‐HA resting *T*_core_ (global mean: 37.2°C ± 0.2°C) was based on 103 observations (*n* = 1014) across 82 papers. For a 0.5°C higher pre‐HA resting *T*_core_, a further change of −0.08°C (−0.13, −0.02; Pd = 99%) was estimated. The change in resting *T*_core_ model accounting for pre‐HA $\dot{V}$O_2peak_ (global mean: 53 ± 7 mL·kg^−1^·min^−1^) was based on 93 observations (*n* = 913) across 73 papers. For a 15 mL·kg^−1^·min^−1^ lower pre‐HA $\dot{V}$O_2peak_, a further change of −0.01°C (−0.07, 0.05; Pd = 61%) was estimated.

##### 3.2.3.2 End‐Exercise Core Temperature

**FIGURE 6:**
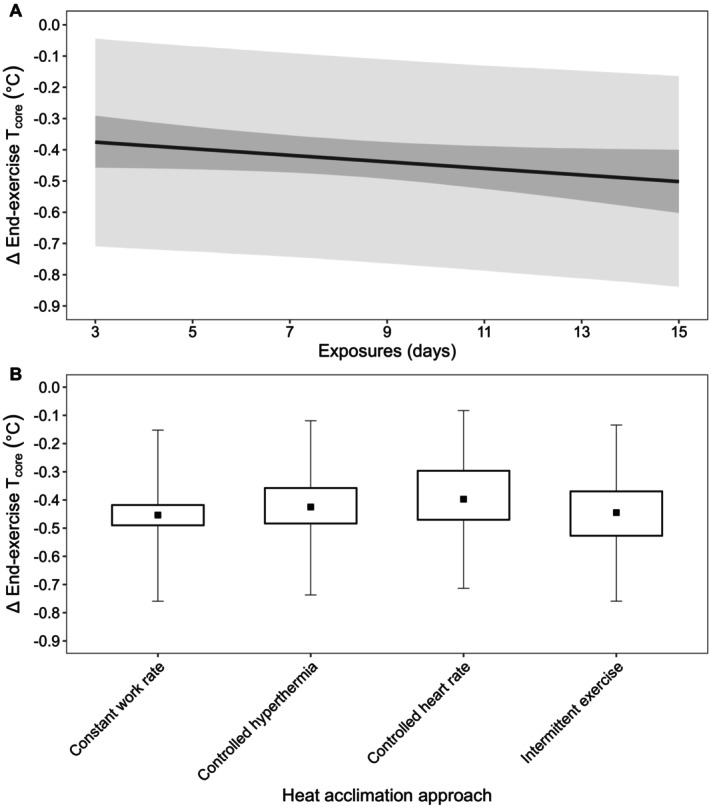
(A) Estimated change (Δ) in end‐exercise core temperature (*T*_core_) across days of exposure based on the global means of the predictor variables (*T*_a_: 39.7°C, *P*_a_: 2.69 kPa, 95 min per exposure). The inner dark gray band represents the 90% CrI and the outer light gray band denotes the 90% PrI. (B) Estimated change in end‐exercise *T*_core_ based on the heat acclimation approach with 90% CrI (box) and 90% PrI (error bars). Estimates are based on the global means of the predictor variables for a mean duration of 8.1 days.

The end‐exercise *T*_core_ model was based on 136 observations (*n* = 1275) across 115 papers, with mean protocol characteristics of: 39.7°C ± 4.9°C, 2.69 ± 0.88 kPa, 8.1 ± 2.5 days, and 95 ± 39 min per exposure. Using these global means and based on a weighted average HA approach, a change in end‐exercise *T*_core_ of −0.43°C (−0.48, −0.36; Pd > 99%) was estimated, with a change of −0.01°C (−0.02, 0.00; Pd = 93%) per additional day of exposure (Figure 6A). The estimated change in end‐exercise *T*_core_ was −0.04°C (−0.05, −0.03; Pd > 99%) for a 15‐min increase in exposure duration, −0.05°C (−0.09, −0.02; Pd > 99%) for a 5°C increase in *T*_a_, and 0.03°C (−0.01, 0.07; Pd = 88%) for a 1 kPa increase in *P*_a_. The estimated change in end‐exercise *T*_core_ was −0.45°C (−0.49, −0.42; Pd > 99%; *n* = 821) for constant workrate HA, −0.43°C (−0.48, −0.36; Pd > 99%; *n* = 238) for controlled hyperthermia HA, −0.41°C (−0.47, −0.30; Pd > 99%; *n* = 75) for controlled HR HA, and −0.44°C (−0.53, −0.37; Pd > 99%; *n* = 59) for intermittent exercise‐HA (Figure 6B).

##### 3.2.3.3 End‐Exercise Skin Temperature

**FIGURE 7:**
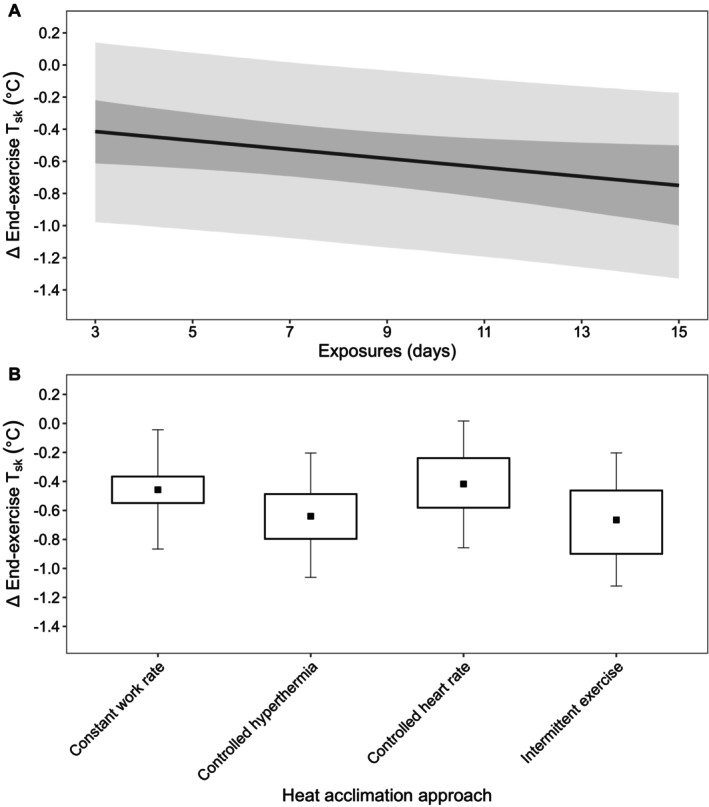
(A) Estimated change (Δ) in end‐exercise mean skin temperature (*T*_sk_) across days of exposure based on the global means of the predictor variables (*T*_a_: 39.8°C, *P*_a_: 2.76 kPa, 90 min per exposure). The inner dark gray band represents the 90% CrI and the outer light gray band denotes the 90% PrI. (B) Estimated change in end‐exercise *T*_sk_ based on the heat acclimation approach with 90% CrI (box) and 90% PrI (error bars). Estimates are based on the global means of the predictor variables for a mean duration of 7.6 days.

The end‐exercise *T*_sk_ model was based on 58 observations (*n* = 548) across 47 papers, with mean protocol characteristics of: 39.8°C ± 4.9°C, 2.76 ± 0.89 kPa, 7.6 ± 2.4 days, and 90 ± 36 min per exposure. Using these global means and based on a weighted average HA approach, a change in end‐exercise *T*_sk_ of −0.54°C (−0.71, −0.39; Pd > 99%) was estimated, with a change of −0.03°C (−0.05, 0.00; Pd = 96%) per additional day of exposure (Figure 7A). The estimated change in end‐exercise *T*_sk_ was −0.02°C (−0.06, 0.01; Pd = 88%) for a 15‐min increase in exposure duration, −0.18°C (−0.25, −0.10; Pd > 99%) for a 5°C increase in *T*_a_, and 0.06°C (−0.03, 0.14; Pd = 88%) for a 1 kPa increase in *P*_a_. The estimated change in end‐exercise *T*_sk_ based on the HA approach was −0.46°C (−0.55, −0.37; Pd > 99%; *n* = 296) for constant workrate HA, −0.64°C (−0.80, −0.49; Pd > 99%; *n* = 130) for controlled hyperthermia HA, −0.42°C (−0.58, −0.24; Pd > 99%; *n* = 47) for controlled HR HA, and −0.66°C (−0.90, −0.46; Pd > 99%; *n* = 34) for intermittent exercise‐HA (Figure 7B).

#### 3.2.4 Sweating Responses

##### 3.2.4.1 Whole Body Sweat Rate

**FIGURE 8:**
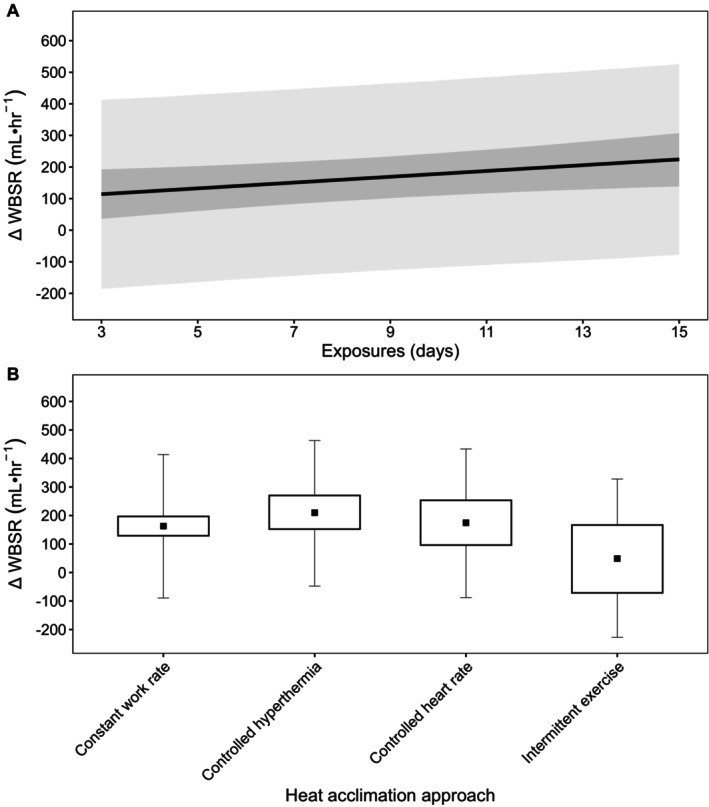
(A) Estimated change (Δ) in whole‐body sweat rate (WBSR) across days of exposure based on the global means of the predictor variables (*T*_a_: 39.1°C, *P*_a_: 2.77 kPa, 90 min per exposure). The inner dark gray band represents the 90% CrI and the outer light gray band denotes the 90% PrI. (B) Estimated change in WBSR based on the heat acclimation approach with 90% CrI (box) and 90% PrI (error bars). Estimates are based on the global means of the predictor variables for a mean duration of 8.2 days.

The WBSR model was based on 138 observations (*n* = 1284) across 113 papers, with mean protocol characteristics of: 39.1°C ± 4.9°C, 2.77 ± 0.80 kPa, 8.2 ± 3.0 days, and 90 ± 33 min per exposure. Using these global means and based on a weighted average HA approach, a change in WBSR of 163 mL·h^−1^ (94, 226; Pd > 99%) was estimated, with a change of 9 mL·h^−1^ (1, 17; Pd = 97%) per additional day of exposure (Figure 8A). The estimated change in WBSR was −11 mL·h^−1^ (−23, 2; Pd = 93%) for a 15‐min increase in exposure duration, 16 mL·h^−1^ (−12, 45; Pd = 82%) for a 5°C increase in *T*_a_, and 37 mL·h^−1^ (4, 72; Pd = 97%) for a 1 kPa increase in *P*_a_. The estimated change in WBSR based on the HA approach was 163 mL·h^−1^ (129, 197; Pd > 99%; *n* = 667) for constant workrate HA, 209 mL·h^−1^ (152, 270; Pd > 99%; *n* = 323) for controlled hyperthermia HA, 174 mL·h^−1^ (96, 253; Pd > 99%; *n* = 75) for controlled HR HA, and 49 mL·h^−1^ (−71, 167; Pd = 74%; *n* = 86) for intermittent exercise‐HA (Figure 8B).

##### 3.2.4.2 Upper Back Local Sweat Rate

The upper back LSR model was based on 13 observations (*n* = 128) across 11 papers, with mean protocol characteristics of: 38.3°C ± 3.1°C, 3.31 ± 0.75 kPa, 7.5 ± 2.0 days, and 96 ± 10 min per exposure. Using these global means, a change in upper back LSR of 0.17 mg·cm^−2^·min^−1^ (0.06, 0.28; Pd = 99%) was estimated, with a change of 0.00 mg·cm^−2^·min^−1^ (−0.02, 0.02; Pd = 51%) per additional day of exposure. The estimated change in upper back LSR was 0.02 mg·cm^−2^·min^−1^ (−0.03, 0.07; Pd = 75%) for a 15‐min increase in exposure duration, −0.06 mg·cm^−2^·min^−1^ (−0.15, −0.03; Pd = 87%) for a 5°C increase in *T*_a_, and −0.03 mg·cm^−2^·min^−1^ (−0.13, 0.07; Pd = 71%) for a 1 kPa increase in *P*_a_.

##### 3.2.4.3 Forearm Local Sweat Rate

The forearm LSR model was based on 15 observations (*n* = 129) across 12 papers, with mean protocol characteristics of: 37.7° ± 4.4°C, 3.19 ± 0.88 kPa, 7.5 ± 2.4 days, and 94 ± 13 min per exposure. Using these global means, a change in forearm LSR of 0.27 mg·cm^−2^·min^−1^ (0.20, 0.34; Pd > 99%) was estimated, with a change of 0.00 mg·cm^−2^·min^−1^ (−0.02, 0.01; Pd = 57%) per additional day of exposure. The estimated change in forearm LSR was 0.00 mg·cm^−2^·min^−1^ (−0.04, 0.05; Pd = 57%) for a 15‐min increase in exposure duration, −0.05 mg·cm^−2^·min^−1^ (−0.10, 0.01; Pd = 91%) for a 5°C increase in *T*_a_, and −0.03 mg·cm^−2^·min^−1^ (−0.10, 0.04; Pd = 78%) for a 1 kPa increase in *P*_a_.

##### 3.2.4.4 Sweat Sodium Concentration

The sweat [Na^+^] model was based on 16 observations (*n* = 132) across 14 papers, with mean protocol characteristics of: 38.1° ± 3.9°C, 3.11 ± 0.69 kPa, 8.1 ± 2.8 days, and 95 ± 10 min per exposure. Using these global means, a change in sweat [Na^+^] of −20.3 mmol·L^−1^ (−27.6, −13.0; Pd > 99%) was estimated, with a change of 0.0 mmol·L^−1^ (−1.8, 1.8; Pd = 50%) per additional day of exposure. The estimated change in sweat [Na^+^] was −2.4 mmol·L^−1^ (−7.0, 2.3; Pd = 80%) for a 15‐min increase in exposure duration, 3.7 mmol·L^−1^ (−5.2, 12.0; Pd = 77%) for a 5°C increase in *T*_a_, and −0.2 mmol·L^−1^ (−9.0, 8.7; Pd = 52%) for a 1 kPa increase in *P*_a_.

#### 3.2.5 Exercise Capacity and Performance

##### 3.2.5.1 Time to Exhaustion

**FIGURE 9:**
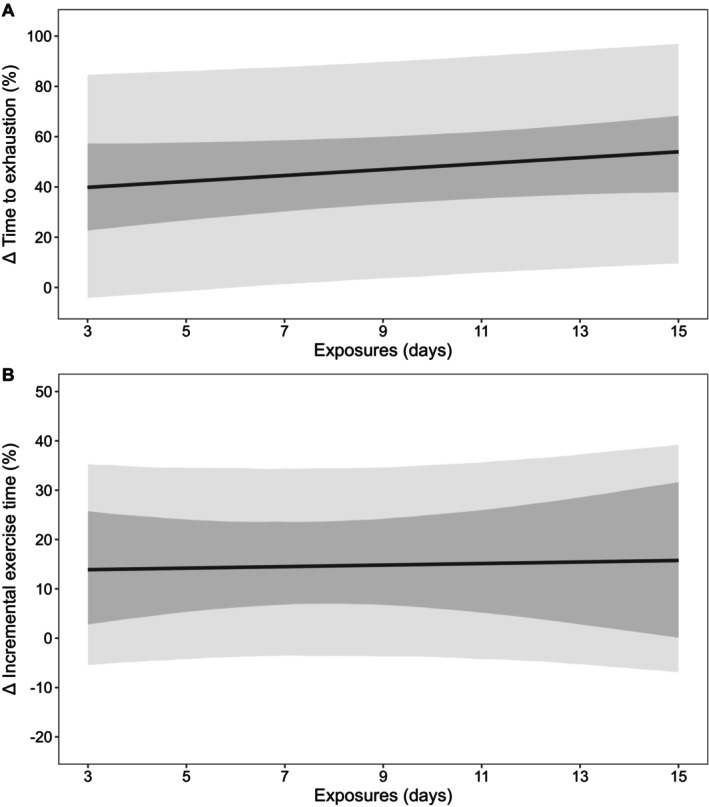
(A) Estimated change (Δ) in time to exhaustion, and (B) incremental exercise over days of exposure based on the global means of the predictor variables (A: *T*_a_: 38.5°C, *P*_a_: 2.13 kPa, 78 min per exposure; B: *T*_a_: 34.9°C, *P*_a_: 3.49 kPa; and 88 min per exposure). The inner dark gray band represents the 90% CrI and the outer light gray band denotes the 90% PrI.

The time to exhaustion test model was based on 10 observations (*n* = 148) across 10 papers, with mean protocol characteristics of: 38.5°C ± 3.7°C, 2.13 ± 1.14 kPa, 10.3 ± 6.8 days, and 78 ± 28 min per exposure. Using these global means, a change in time to exhaustion of 48.7% (34.7, 61.2; Pd > 99%) was estimated, with a change of 1.2% (−0.5, 2.7; Pd = 89%) per additional day of exposure (Figure 9A). The estimated change in time to exhaustion was 3.4% (−1.1, 7.7; Pd = 89%) for a 15‐min increase in exposure duration, 8.6% (−2.9, 19.5; Pd = 89%) for a 5°C increase in *T*_a_, and −5.3% (−12.3, 1.9; Pd = 89%) for a 1 kPa increase in *P*_a_.

##### 3.2.5.2 Incremental Exercise

The incremental exercise test model was based on eight observations (*n* = 88) across five papers, with mean protocol characteristics of: 34.9°C ± 3.1°C, 3.49 ± 0.57 kPa, 7.5 ± 2.7 days, and 88 ± 20 min per exposure. Using these global means, a change in incremental exercise test capacity of 14.2% (6.7, 23.6; Pd > 99%) was estimated, with a change of 0.2% (−1.6, 1.9; Pd = 57%) per additional day of exposure (Figure 9B). The estimated change in incremental exercise test capacity was −2.7% (−6.1, 1.5; Pd = 87%) for a 15‐min increase in exposure duration, 3.9% (−6.1, 13.6; Pd = 74%) for a 5°C increase in *T*_a_, and 0.1% (−7.2, 7.6; Pd = 51%) for a 1 kPa increase in *P*_a_.

##### 3.2.5.3 Time Trial Performance

**FIGURE 10:**
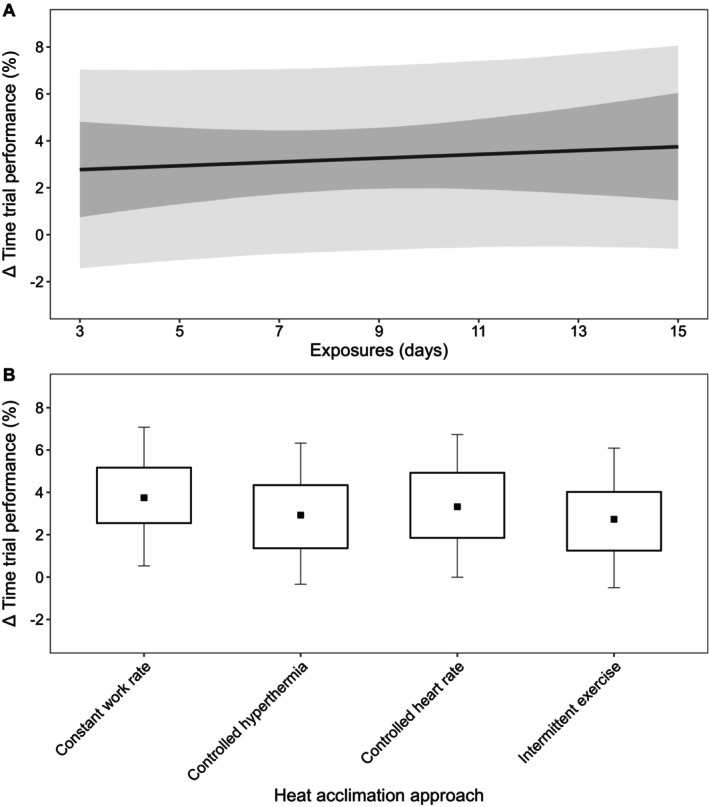
(A) Estimated change (Δ) in time trial performance across days of exposure based on the global means of the predictor variables (*T*_a_: 35.8°C, *P*_a_: 2.95 kPa, 69 min per exposure). The inner dark gray band represents the 90% CrI and the outer light gray band denotes the 90% PrI. (B) Estimated change in time trial performance based on the heat acclimation approach with 90% CrI (box) and 90% PrI (error bars). Estimates are based on the global means of the predictor variables for a mean duration of 7.6 days.

The time‐trial performance model was based on 23 observations (*n* = 211) across 17 papers, with mean protocol characteristics of: 35.8°C ± 3.2°C, 2.95 ± 0.62 kPa, 7.6 ± 3.0 days, and 69 ± 22 min per exposure. Using these global means and based on a weighted average HA approach, a change in performance of 3.1% (1.8, 4.5; Pd > 99%) was estimated, with a change of 0.1% (−0.2, 0.4; Pd = 69%) per additional day of exposure (Figure 10A). The estimated change in time trial performance was 0.1% (−0.6, 0.8; Pd = 58%) for a 15‐min increase in exposure duration, −0.5% (−2.1, 1.0; Pd = 73%) for a 5°C increase in *T*_a_, and 0.2% (−1.1, 1.6; Pd = 61%) for a 1 kPa increase in *P*_a_. The estimated change in performance based on HA approach was 3.7% (2.5, 5.2; Pd > 99%; *n* = 73) for constant workrate HA, 3.0% (1.4, 4.3; Pd > 99%; *n* = 49) for controlled hyperthermia HA, 3.3% (1.9, 4.9; Pd > 99%; *n* = 32) for controlled HR HA, and 2.8% (1.3, 4.0; Pd > 99%; *n* = 47) for intermittent exercise‐HA (Figure 10B).

## 4 Discussion

The aim of this review was to quantify the adaptive response to exercise‐HA and determine the influence of protocol characteristics on adaptation kinetics. Our analyses indicated that exercise‐HA (~8 days, ~90 min per exposure, ~39.1°C, and ~2.78 kPa) lowered resting HR (5 beats·min^−1^), end‐exercise HR (17 beats·min^−1^), resting *T*_core_ (0.19°C), end‐exercise *T*_core_ (0.43°C), end‐exercise *T*_sk_ (0.54°C), and exercise metabolic rate (87 mL·min^−1^) during a heat response test (92 ± 49 min; range: 15– 240 min). Exercise‐HA also expanded BV (2.9%), PV (5.6%), and RCV (1.3%), increased WBSR (163 mL·h^−1^), and improved time to exhaustion (49%), incremental exercise capacity (14%), and time trial performance (3%). Our analyses also indicated that the addition of one exposure day further lowered resting *T*_core_ (0.01°C) and end‐exercise *T*_sk_ (0.03°C), and increased Hb_mass_ (1.9 g) and WBSR (9 mL·h^−1^), while increasing exposure duration by 15 min further lowered end‐exercise HR (1 beat·min^−1^) and end‐exercise *T*_core_ (0.04°C) and expanded PV (0.4%). A 5°C higher *T*_a_ during HA further lowered end‐exercise HR (2 beats·min^−1^), end‐exercise *T*_core_ (0.05°C), and end‐exercise *T*_sk_ (0.18°C), whereas a 1 kPa increase in *P*_a_ increased RCV (0.7%) and enhanced WBSR (37 mL·h^−1^). To guide individualized HA prescription for athletes and military/occupational personnel or exercise physiology experiments, an online tool for estimating HA adaptations based on protocol characteristics is publicly available via the web site (https://www.canberra.edu.au/research/centres/uc‐rise/research/environmental‐physiology/exercise‐heat‐acclimation‐predictor).

### 4.1 Cardiovascular Adaptations

Our analyses revealed that exercise‐HA decreased resting (5 beats·min^−1^) and end‐exercise HR (17 beats·min^−1^), and lowered exercise metabolic rate (87 mL·min^−1^; Figures 2 and 3). The changes in resting and end‐exercise HR differed widely between the papers included in this review (Figures S1 and S2), ranging from a 4 beats·min^−1^ increase in resting HR (Aoyagi et al. 1994) to an 18 beats·min^−1^ reduction (Gibson, Turner, et al. 2015), and from a 1 beat·min^−1^ increase in end‐exercise HR (Aoyagi et al. 1994; Travers et al. 2021) to a reduction of > 30 beats·min^−1^ (Rowell et al. 1967; Shapiro et al. 1980; Kotze et al. 1977; Pandolf et al. 1977; Finberg and Berlyne 1977; Shvartz et al. 1977). For end‐exercise HR, increasing *T*_a_ and exposure duration during HA led to further reductions of 2 and 1 beat·min^−1^, respectively. These novel observations highlight the intricate interaction between HA protocol characteristics and adaptation kinetics, with specific adjustments in characteristics having the potential to impact the HA phenotype.

A large portion of the reduction in HR during exercise occurs within the first week of HA (Griefahn 1997; Pandolf et al. 1988), which is supported by our findings that demonstrate no influence of an additional exposure day on end‐exercise HR (Figure 3A). It has also been suggested that constant workrate HA may result in incomplete adaptations compared to controlled hyperthermia HA (Tyler et al. 2016, 2024); however, our results indicate a similar reduction in end‐exercise HR between approaches (15–17 beats·min^−1^). When comparing HA approaches, the reduction in resting HR was also within 4 beats·min^−1^ between constant workrate (−5 beats·min^−1^), controlled hyperthermia (−8 beats·min^−1^), controlled HR (−4 beats·min^−1^), and intermittent exercise HA (−5 beats·min^−1^). A sub‐analysis revealed that an additional reduction of ~3 beats·min^−1^ in resting HR occurred when pre‐HA resting HR was 15 beats·min^−1^ higher than the global mean of 72 beats·min^−1^. In line with this observation, reductions of > 10 beats·min^−1^ have been demonstrated when pre‐HA resting HR was ≥ 75 beats·min^−1^ (Rendell et al. 2017; Moss et al. 2020; Magalhães et al. 2010; Lee et al. 2016), with studies having lower pre‐HA values (52–57 beats·min^−1^) often reporting smaller reductions (2–4 beats·min^−1^; Osborne et al. 2021; Travers, Nichols, et al. 2020; James et al. 2017). It was anticipated that a similar relationship would exist with a lower pre‐HA $\dot{V}$O_2peak_, but this was not the case. Nevertheless, these data indicate that individuals with a higher resting HR, which are typically less aerobically trained (Reimers et al. 2018), have a greater potential for adaptations.

For exercising metabolic rate, our analysis revealed a reduction of ~87 mL·min^−1^ from pre‐ to post‐HA, with one paper reporting an improvement in exercise economy of 181 mL·min^−1^ (Shvartz et al. 1977). Historically, improvements in metabolic rate have been shown to be more pronounced in unfit (181 mL·min^−1^; $\dot{V}$O_2peak_: 36 mL·kg^−1^·min^−1^) than highly fit individuals (121 mL·min^−1^: $\dot{V}$O_2peak_: 60 mL·kg^−1^·min^−1^; Shvartz et al. 1977). Several mechanistic pathways have been suggested to mediate the decrease in metabolic rate following HA, including an increase in BV allowing for a greater fraction of cardiac output to perfuse exercising muscles (Rowell 1974; Senay et al. 1976). Support for this pathway is limited, however, with reports of leg blood flow remaining similar from pre‐ to post‐HA (Nielsen et al. 1993; Kirwan et al. 1987). Others have suggested that HA lowers metabolic rate by improving muscular efficiency (Jansen et al. 1990), possibly via alterations in motor unit recruitment patterns that favor slow‐twitch fiber utilization (Sawka et al. 1983). Further reports have shown lower glycogen utilization in type 1 fibers (Kirwan et al. 1987; Febbraio et al. 1994; King et al. 1985) and a slight increase in lipid oxidation following HA (Kirwan et al. 1987). It has also been shown that HA improves skeletal muscle contractile function, along with the torque/electromyographic relationship (Racinais et al. 2017), which may contribute to improve exercise economy during exercise at a given workrate post‐HA. Alternatively, the decrease in metabolic rate following HA may relate to a reduction in plasma epinephrine (Febbraio et al. 1994), which during exercise increases glycogenolytic rate by stimulating the conversion of phosphorylase to its active form (Richter 1984). Febbraio et al. (1994) demonstrated that a post‐HA reduction in plasma epinephrine was accompanied by decreases in muscle lactate accumulation, blood glucose, and lactate concentration. Given the limited research in this area, however, the mechanistic pathway(s) via which metabolic rate is reduced following HA remains to be elucidated.

### 4.2 Hematological Responses

Hematological adaptations in response to repeated heat exposure are well documented and are typically characterized by an increase in BV due to the rapid expansion of PV (Horowitz 2014; Périard et al. 2021). The reduction in HR during exercise has been partly attributed to this expansion of PV (Sawka et al. 2011; Sawka 1996; Périard et al. 2016), which may also contribute to increase ventricular filling and stroke volume, allowing for cardiac output and blood pressure to be better maintained (Keiser et al. 2015; Nielsen et al. 1997). Although additional research is required to fully elucidate this relationship (Wilson et al. 2020; Trachsel et al. 2020; Travers, González‐Alonso, et al. 2020), our model estimated a PV expansion of 5.6% after 8 days of HA, which is slightly greater than other meta‐analyses reporting a 3.8%–4.3% expansion (Tyler et al. 2016, 2024) and much larger than a recent meta‐analysis of HA in females reporting a 1% contraction (Kelly et al. 2023). Of note, the PV analysis conducted in the current review included a much larger sample (*n* = 859) than previous meta‐analyses primarily in males (Tyler et al. 2016: *n* = 209, Tyler et al. 2024: *n* = 354) and females only (Kelly et al. 2023: *n* = 28), with our data highlighting the pronounced variability between studies, even with similar HA protocol characteristics (Figure S5).

The estimated change in BV following HA from a sample of 258 participants in the current study was 2.9%. Our results did not indicate any influence of HA protocol characteristics on changes in BV, other than a potential link with an additional 15 min of exposure (i.e., 0.7% further increase in BV). Another possible link, albeit small, between PV expansion and exposure duration was noted, with a 0.4% further increase estimated per additional 15 min of exposure, which contrasts with a previous meta‐analysis reporting no association (Tyler et al. 2016). The mechanisms of fluid retention and PV expansion with HA relate to the combined osmotic pressure exerted by proteins (e.g., albumin) and electrolytes (e.g., sodium) across fluid compartments (Harrison et al. 1981; Senay 1979). These changes in osmotic pressure result in the intravascular influx of fluid from the interstitial compartment, increasing PV. As such, the potential link between HA exposure duration and PV expansion is unclear and likely to be an artifact associated with measurement errors (Gore et al. 2005; Harrison 1985). For example, Senay et al. (1976) reported a mean PV expansion of 23.5% following nine prolonged (240 min) HA exposures. In contrast, the two other studies with 16 and 9 exposures of 240 min reported a PV expansion of 4.8% (Wyndham et al. 1968) and 8.0% (Kotze et al. 1977), respectively. While the relationship between PV expansion and exposure duration is intriguing, it requires clarification.

An increase in RCV of 1.3% was estimated from our analysis after ~12 days of exercise‐HA. Although an increase in RCV is typically accompanied by a concomitant increase in Hb_mass_ (Lundby et al. 2021; Rønnestad, Hamarsland, et al. 2021), based on our model, ~13 days of exercise‐HA did not increase Hb_mass_ (−3.6 g), with only four of the 12 papers reporting an increase (range: 17–42 g; Lundby et al. 2021; Rønnestad, Hamarsland, et al. 2021; Rønnestad et al. 2022; Oberholzer et al. 2019). These papers involved pooling data from three separate 10‐day HA regimens to demonstrate an increase in Hb_mass_ (Lundby et al. 2021), or were conducted over a much longer time frame (~5 weeks of HA; Rønnestad, Hamarsland, et al. 2021; Rønnestad et al. 2022; Oberholzer et al. 2019). Accordingly, it has recently been suggested that prolonged HA regimens are required to increase RCV and Hb_mass_ (Bennett et al. 2024), which is supported by data from studies with 25–40 days of HA using thermal clothing to limit heat loss during exercise in temperate conditions (Rønnestad, Hansen, et al. 2021; Lundby et al. 2023; Cubel et al. 2024). Our analysis supports this view, with an estimated change in Hb_mass_ of 1.9 g for every additional day of heat exposure beyond the global mean of ~13 days (e.g., 19.9 g increase in Hb_mass_ after 25 days of HA). The kinetics of change in RCV and Hb_mass_ with exercise‐HA align somewhat with those of an endurance training study demonstrating a 6% increase in RCV after 4 weeks and a 10% increase in Hb_mass_ after 8 weeks, following the expansion of PV and increase in circulating erythropoietin (EPO) concentration after 2 weeks of training (Montero et al. 2017). The potential pathways for increasing RCV and Hb_mass_ include the acute activation of hypoxia‐inducible factor‐α and EPO release from exercising muscle (Lundby et al. 2006; Rundqvist et al. 2009), as well as post‐exercise peripheral vasodilation prompting the secretion of BV‐regulating hormones and stimulating erythropoiesis (Montero et al. 2016; Piepoli et al. 1993). It may also relate to early PV expansion driving hypoxia feedback loops to restore hematocrit (Sawka et al. 2000; Schmidt and Prommer 2008; Kjellberg et al. 1949), or potential alterations in the secretion patterns of growth hormone, insulin‐like growth factor, testosterone, catecholamines, and cortisol influencing RCV production and/or release from bone marrow (Hu and Lin 2012). Given the limited sample size in this model (*n* = 139), more studies are required to further our understanding of the relationship between exercise‐HA protocol length and increases in RCV and Hb_mass_.

### 4.3 Thermal Responses

The 0.19°C reduction in resting *T*_core_ following exercise‐HA estimated in this meta‐analysis aligns with previous reports conducted primarily in males (Tyler et al. 2016: −0.18°C, Tyler et al. 2024: −0.16°C) and females only (Kelly et al. 2023: −0.15°C). Although our model estimated a further 0.01°C reduction in resting *T*_core_ with an additional exposure day (Figure 5A), it is likely not meaningful as an additional 7 days of HA would only further reduce resting *T*_core_ by ~0.07°C. When examining the impact of HA protocol characteristics on resting *T*_core_, none appeared to have a meaningful influence, although Tyler et al. (2016) previously suggested that maintaining the thermal impulse throughout HA may further reduce resting *T*_core_. This suggestion was largely based on data from Patterson et al. (2004a) who demonstrated that reductions in resting *T*_core_ were greater after 22 days (0.32°C) compared to 8 days (0.20°C) of controlled hyperthermia HA (target *T*_core_: ~38.5°C). In contrast, Gibson, Mee, Tuttle, et al. (2015) reported similar reductions in resting *T*_core_ after 5 (0.20°C) and 10 days (0.19°C) of controlled hyperthermia HA (target *T*_core_: ~38.5°C), with no further reduction experienced in a separate group in which the target *T*_core_ increased to 39.0°C from days 5 to 10 (0.21°C and 0.19°C, respectively). In the current meta‐analysis, the constant workrate (−0.21°C), controlled hyperthermia (−0.20°C), controlled HR (−0.18°C), and intermittent exercise (−0.17°C) HA approaches appear to elicit a similar reduction in resting *T*_core_. These data highlight how the adaptive stimulus (i.e., exercise‐HA) might obscure the potential for a specific HA approach to provide a greater reduction in resting *T*_core_.

A reduction in end‐exercise *T*_core_ of 0.43°C was estimated in the current meta‐analysis, which is similar to Kelly et al. (2023: −0.41°C) and slightly higher than Tyler et al. (2016: −0.34°C). The reduction in *T*_core_ has been reported to primarily develop within the first week of HA (Pandolf 1998), which is in line with the current analysis estimating a small additional benefit (−0.01°C) for every additional exposure (Figure 6A). This notion is strengthened by the large sample size used to estimate the change in end‐exercise *T*_core_ in the current meta‐analysis (*n* = 1261). Notwithstanding, *T*_a_ (5°C increase) and exposure duration (additional 15 min per exposure) were estimated to influence the change in end‐exercise *T*_core_, with a further reduction of 0.05°C and 0.04°C, respectively. Although independently these changes are relatively small, the combination of increasing *T*_a_ and exposure duration over additional HA days is likely to promote a further reduction in end‐exercise *T*_core_.

A lowering of exercising *T*_sk_ following HA is purported to arise from an enhanced sweat rate (Sawka 1996; Périard et al. 2016; Sawka et al. 2010), coupled with greater evaporative heat loss (Poirier et al. 2015). Our analysis estimated a comparable reduction in end‐exercise *T*_sk_ (−0.54°C) to that of a meta‐analysis in females only (−0.50°C; Kelly ey al. 2023), and predominantly in males (−0.57; Tyler et al. 2016). Our analysis extends these observations to indicate that end‐exercise *T*_sk_ is further lowered by a 5°C increase in *T*_a_ (0.18°C) and an additional day of exposure (0.03°C) during HA (Figure 7A). While these further reductions in end‐exercise *T*_sk_ may appear trivial, our modeling indicates that increasing *T*_a_ by more than 5°C during HA and/or adding several HA days would promote a more robust adaptive response. Moreover, when comparing the influence of HA approaches on the change in end‐exercise *T*_sk_, controlled hyperthermia (−0.64°C) appeared to provide a stronger stimulus compared to constant workrate HA (−0.46°C; Figure 7B), which may partly be explained by the slightly larger increase in WBSR (209 mL·h^−1^ vs. 163 mL·h^−1^), assuming additional evaporation. Changes in end‐exercise *T*_sk_ were also noted following intermittent exercise (−0.66°C) and controlled HR (−0.42°C) HA, although in much smaller samples (*n* = 34 and 47, respectively).

### 4.4 Sweating Responses

Data from our meta‐analysis indicate an increase in WBSR of 163 mL·h^−1^ following ~8 days of exercise‐HA (Figure 8A), with large heterogeneity between studies (Figure S11). Indeed, several studies documented increases in WBSR of ≥ 500 mL·h^−1^ (Lundby et al. 2021; Lee et al. 2016; Gibson, Mee, Tuttle, et al. 2015; Curley and Hawkins 1983), with values included in the analysis ranging from −300 to 890 mL·h^−1^ (Lee and Thake 2017; Roussey et al. 2021). Despite the 5%–7% day‐to‐day variability in individual WBSR (Baker et al. 2009; Hayden et al. 2004), several studies documented a lack of change in WBSR with exercise‐HA (Shapiro et al. 1980; Pandolf et al. 1977; Roussey et al. 2021; Amano et al. 2015, 2016; Aoyagi et al. 1995a, 1998; Brock et al. 1982; Frank et al. 2001; Hahn et al. 1986; Kelly et al. 2016; Petersen et al. 2010; Reeve et al. 2019; Rivas et al. 2017; Takeno et al. 2001; Willmott et al. 2016; Armstrong et al. 1987; McGlynn et al. 2022; Waldock et al. 2021), which is somewhat surprising given that an increase in WBSR is a hallmark heat adaptation (Sawka 1996). The heterogeneity in WBSR adaptations may stem from methodological issues with heat response testing (e.g., short‐duration or intermittent heat response tests, and/or post‐testing conducted in less thermally challenging environments).

Of the HA protocol characteristics potentially influencing WBSR, the addition of one exposure day (9 mL·h^−1^) and 1 kPa (37 mL·h^−1^) emerged as influential. Tyler et al. (2016) previously reported a strong‐positive relationship between *T*_a_ and WBSR, but not relative humidity. The discrepancy in findings may stem from our analysis using *P*_a_, which reflects absolute humidity at a given *T*_a_ and thus the total amount of water vapor in the air, and the potential for evaporative heat loss (Gagge and Hardy 1967; Gagge and Gonzalez 2010). In contrast, relative humidity is the amount of water vapor in the air expressed as a percentage of the amount of water vapor needed to achieve saturation at a given temperature, which is not reflective of evaporative heat loss potential (Kerslake 1972). To the best of our knowledge, there are only three studies to date that have directly compared exercise‐HA in a range of environments with different *P*_a_ (Shvartz et al. 1973; Griefahn 1997; Tebeck et al. 2020), alongside two separate studies with similar designs conducted in low (Nielsen et al. 1993) and high *P*_a_ environments (Nielsen et al. 1997). Data from these studies indicate the potential for exercise‐HA undertaken in environments with high *P*_a_ (4.42–5.65 kPA) to elicit a slightly greater increase in WBSR compared to low‐moderate *P*_a_ (1.68–3.45 kPa) (Shvartz et al. 1973; Griefahn 1997; Nielsen et al. 1993, 1997). Of these studies, Griefahn (1997) reported a slightly larger change in WBSR following HA in high (132 mL·h^−1^) compared to low (89 mL·h^−1^) *P*_a_ conditions, noting that the change in high *P*_a_ conditions was highly variable. Shvartz et al. (1973) reported a 234 and 128 mL·h^−1^ increase in WBSR after exercise‐HA in 5.5 and 3.5 kPa conditions, respectively, although no direct comparison between conditions was made. This is similar to Nielsen et al. (1993, 1997) who demonstrated a larger WBSR increase after exercise‐HA in high (26%; Nielsen et al. 1997) compared to low (17%; Nielsen et al. 1993) *P*_a_ environments in separate studies. In contrast, Tebeck et al. (2020) reported improvements in WBSR following exercise‐HA in low *P*_a_ conditions (200 mL·h^−1^) and no change in high *P*_a_. The latter observation may relate to the 5‐day HA protocol not being long enough to induce robust sudomotor adaptations, with our model estimating a slightly more pronounced WBSR increase with additional exposure days (9 mL·h^−1^) beyond the initial 8 days of exercise‐HA (Figure 8A). However, it is important to note the limited number of HA papers with 15 or more days of exposure (Griefahn 1997; Patterson et al. 2004a, 2014; Fortney and Vroman 1985; Racinais et al. 2024), such that caution should be used when extrapolating how WBSR may increase with additional exposures. Further research with longer regimens is thus required to develop a better understanding of the potential for WBSR changes to occur over a protracted timeframe, in hot‐dry and warm‐humid environments, as well as the cross‐acclimation potential between these environments. Notwithstanding, our model supports a further increase in WBSR with HA undertaken in high relative to low *P*_a_ conditions, despite a lack of evidence from studies directly comparing HA in dry and humid conditions.

To the best of our knowledge, this is the first meta‐analysis to examine LSR following exercise‐HA, with changes in both the upper back (0.17 mg·cm^−2^·min^−1^) and forearm (0.27 mg·cm^−2^·min^−1^) estimated from our models. These estimates align with a recent 7‐day HA study demonstrating increases in upper back and forearm LSR of 0.18 and 0.33 mg·cm^−2^·min^−1^, respectively (Lynch et al. 2024). The slightly greater increase in forearm LSR likely relates to interregional variations in the capacity to increase LSR with HA, which has previously been demonstrated following hot‐dry (Smith and Havenith 2019) and hot‐humid HA (Patterson et al. 2004a). Our data are thus in agreement with the relative preferential redistribution of LSR toward the forearm following HA (Patterson et al. 2004a; Smith and Havenith 2019). The greater improvement in LSR of the upper limbs and preferential shift toward the periphery contributes to a more uniform distribution of skin wettedness, potentially leading to a higher evaporative efficiency (Smith and Havenith 2019). However, given the limited LSR data, along with the low inter‐day reliability (Peel et al. 2022) and high inter‐study variability (Figures S12 and S13), additional research is required to better characterize the impact of HA protocol characteristics on LSR.

The increase in WBSR during HA typically occurs alongside a reduction in sweat [Na^+^] (Keiser et al. 2015; Rendell et al. 2017; Patterson et al. 2014; Kaufman et al. 1988; McCleave et al. 2019; Neal, Massey et al. 2016; Neal, Corbett, et al. 2016). Our findings align with this notion as a reduction in sweat [Na^+^] of 20 mmol·L^−1^ was estimated following ~8 days of exercise‐HA. However, while the increase in WBSR is commonly suggested to develop over a longer period (Sawka et al. 2011; Périard et al. 2021; Daanen et al. 2018), which is supported by our WBSR model, this pattern was not evident with sweat [Na^+^] (i.e., no change per additional day of exposure). This suggests that the reduction in sweat [Na^+^] may occur more rapidly than the increase in WBSR. However, similar to the LSR analysis, a lack of studies examining sweat [Na^+^] does not allow for drawing robust inferences regarding the influence of HA protocol characteristics on changes in sweat [Na^+^]. Nevertheless, sweat [Na^+^] decreases following HA, which leads to a more dilute sweat and may improve evaporative heat loss due to an increase in the saturated water vapor pressure at the surface of the skin for a given temperature (Kerslake 1972). Reductions in sweat [Na^+^] are thus an important adaptation following HA (Périard et al. 2015), particularly in humid environments where evaporative efficiency is reduced due to an elevated *P*_a_.

### 4.5 Exercise Capacity and Performance

Our analysis estimated a large improvement in time to exhaustion (48.7%), with 70% of the included papers showing an improvement following exercise‐HA (Burk et al. 2012; Kaldur et al. 2014; Oöpik et al. 2014; Tamm et al. 2015, Nielsen et al. 1993, 1997; Mikkelsen et al. 2019) and one reporting an impairment (27.2%), which the authors attributed to a lack of recovery following a short‐term high‐intensity protocol (Reeve et al. 2019). The estimated improvement from our analysis is effectively double the 22% and 23% improvement in exercise capacity reported by Tyler et al. (2016, 2024), and likely relates to our analysis separating time to exhaustion and incremental exercise tests, rather than analyzing them in combination. Indeed, an improvement of 14.2% in incremental exercise time was estimated from our analysis following an exercise preload in the heat (range: 21–90 min), with all papers reporting an improvement following exercise‐HA (Gale et al. 2021; Shaw et al. 2022; Alkemade et al. 2021; Garrett et al. 2009; Gerrett et al. 2021). The magnitude of improvement in incremental exercise capacity was substantially less than that of time to exhaustion, likely due to the distinctiveness of the protocols and the vastly different mean baseline exercise times (8.5 min vs. 65.4 min, respectively). This difference reinforces the rationale for separating time to exhaustion and incremental exercise tests when investigating changes in exercise capacity following HA.

In a recent meta‐analysis of HA and exercise capacity, Benjamin et al. (2019) reported an improvement of 144.30 s in time to exhaustion based on 24 papers. However, given the use of different inclusion/exclusion criteria, such as time to exhaustion tests in both temperate (< 25°C) and hot (≥ 25°C) conditions, as well as time‐based heat response tests (Benjamin et al. 2019), it is difficult to draw direct comparisons with our data. In our analysis, time to exhaustion tests were based on the attainment of volitional fatigue, or a physiological/ethical cutoff point (*T*_core_ > 40°C or 95% HR_max_) in hot conditions (≥ 30°C), and as such excluded studies that had a predefined endpoint based on time (Pandolf et al. 1988; Gibson, Mee, Tuttle, et al. 2015; Aoyagi et al. 1998; Horstman and Christensen 1982). The rationale for this decision was based on participants exercising until a similar physiological endpoint to that of the pre‐acclimation test, rather than a time point that may or may not have been reached during the pre‐acclimation heat response test.

Along with changes in exercise capacity, HA improves performance during self‐paced (i.e., time trial) exercise in hot environments. This improvement was highlighted in our analysis by the 3.1% increase in performance, although with no apparent influence of an additional day of exposure (Figure 10A). The smallest improvement in performance was 0.7% (Garrett et al. 2012), with the largest being ~7% (James et al. 2017; Guy et al. 2016). The larger improvements were reported during 5‐km cycling (Guy et al. 2016) and running (James et al. 2017) time trials in 35°C–37°C following 7 and 5 days of controlled hyperthermia HA, respectively. Our analysis failed to identify a relationship between time trial performance and a 5°C increase in *T*_a_ during exercise‐HA (−0.5% [−2.1, 1.0]), which contrasts the weak‐moderate correlation between performance improvements and *T*_a_ reported by Tyler et al. (2016). Taken together, it appears that further research is required to better understand the impact of environmental characteristics during HA on performance, as only 17 papers were included in our model. This call for research also extends to HA approach, despite estimated changes in performance of 2.8%–3.7% across the different approaches, which corroborates the findings of a previous meta‐analysis (Benjamin et al. 2019).

### 4.6 Interorgan Communication

The kinetics of cardiovascular, hematological, thermal, and sweating adaptations related to exercise‐heat stress are underpinned by molecular and interorgan communication signals that coordinate integrated physiological responses. Indeed, molecular and cellular mechanisms provide the foundation for the phenotypical changes associated with hyperthermia and HA (Horowitz 2014; Murray et al. 2022). During exercise‐heat exposure, thermal stress is sensed by peripheral and central thermoreceptors (Boulant and Dean 1986; Filingeri 2016). These receptors provide afferent input to the hypothalamic thermoregulatory center, where they are integrated and produce efferent neural signals for heat dissipation via cutaneous vasodilation and eccrine sweating (Ravanelli et al. 2021; Romanovsky 2018). With repeated heat exposures, neural adaptations occur that lower the temperature threshold required to initiate heat loss responses, and peripheral neurotransmitter adaptations in the cutaneous vasculature and eccrine sweat glands augment the sensitivity of these responses (Horowitz 2014; Sawka et al. 2011; Werner 1994). The improved capacity to dissipate heat attenuates thermal strain during exercise in the heat and contributes to reduce resting core temperature.

The cardiovascular response to heat stress includes increased cutaneous vasodilation, which elevates cardiac output (Crandall and Wilson 2015). The ability to sustain cardiac output is challenged during prolonged exercise in the heat by reduced cardiac filling pressures and difficulty defending blood pressure, especially with dehydration (Périard et al. 2021; Trangmar and González‐Alonso 2019). However, the repeatedly elevated cardiac output (i.e., chronic volume overload) with HA improves myocardial contractility and efficiency (in the animal model; Levy et al. 1997), which supports cardiac output and blood pressure regulation (Sawka et al. 2011). Likewise, improvements in the sweating response lower skin temperature and increase the core‐to‐skin temperature gradient, allowing for reduced skin blood flow requirements, and thus cardiac output, contributing to lower heart rate during exercise‐heat stress. Cardiac output and blood pressure regulation are further supported by HA‐induced BV and PV expansion, which also supports thermoregulation.

Elevated tissue temperatures during exercise‐heat stress induce cellular stress responses and under‐perfusion activates oxidative and nitrogen species stress signaling pathways. These communication signals upregulate heat shock protein and cytokine responses to provide tissue/organ protection (Sawka et al. 2011; Hasday et al. 2014). The endocrine system is also influenced by the HA phenotype, with adjustments in signals originating from the hypothalamus, pituitary, adrenal, thyroid, and pancreas (Francesconi 1988; Hannan et al. 2024). Indeed, exercise‐heat exposure alters renin, aldosterone, and vasopressin secretion to expand and conserve body fluids. Likewise, catecholamine, glucocorticoid, growth, and thyroid hormones provide communication signals supporting blood pressure regulation, metabolism, protein synthesis, and inflammatory responses.

Taken together, there is clear interorgan communication involved in the development of the HA phenotype, mediated through coordinated physiological responses and mechanistic pathways. The relative contribution of each response and mechanism to adaptation kinetics is determined by the strength of the perturbation to homeostasis, resulting from HA protocol characteristics.

### 4.7 Limitations

The aim of this review was to provide insights regarding the influence of HA protocol characteristics on the kinetics of adaptation; however, due to the nature of HA studies, certain limitations should be acknowledged. As previously highlighted, there are important intra‐individual differences in HA adaptations within studies (Corbett et al. 2018), which are compounded when comparing studies. The heterogeneity between paper outcomes can be clearly observed in Material S2, which highlights the differences between outcomes based on HA approach and testing. Notwithstanding, a large proportion of HA protocol characteristics within the literature are somewhat homogenous, with several study characteristics being similar to those of the global means (i.e., ~8 days, ~90 min per exposure, ~39.1°C and ~2.78 kPa). Another important consideration is that while the data used in the meta‐analyses were obtained from heat response tests, the characteristics (i.e., *T*_a_, *P*_a_ and duration) of these tests differed at times from those of the HA protocol (Travers et al. 2021; Travers, Nichols, et al. 2020; Travers, González‐Alonso, et al. 2020; Gibson, Mee, Tuttle, et al. 2015; Poirier et al. 2015, 2016; Garrett et al. 2012, 2014, 2019; Flouris et al. 2014; James et al. 2018; Neufer et al. 1989), which were used to estimate changes in outcome variables. However, this is the case for all HA meta‐analyses. Finally, although percentage changes in PV, time to exhaustion, incremental exercise capacity, and time trial performance were modeled in our analyses, which may not be the optimal approach (Vickers 2001), it aligns with the common reporting method for PV in the literature, accounts for the differing baseline times in exercise capacity tests, and allows for combining different types of performance tests (e.g., distance and time‐based time trials). Ultimately, additional studies and/or greater uniformity between pre‐ and post‐HA testing and protocols within and between studies may help strengthen the interpretability of future study findings.

## 5 Conclusion

**FIGURE 11:**
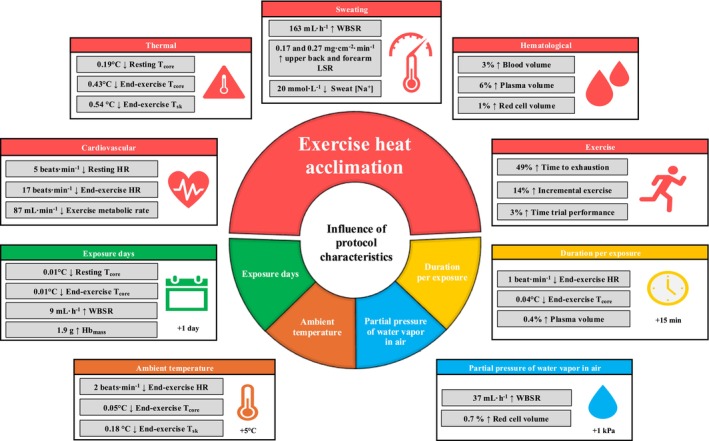
Physiological and performance adaptations associated with exercise heat acclimation (~8 days, 90 min per day, 39.1°C, and 2.78 kPa), along with the influence of adjusting protocol characteristics on adaptation kinetics.

To date, this is the largest systematic review with meta‐analyses to explore the adaptive response and performance/capacity changes associated with HA. A novel feature of our analyses was the integration of HA protocol characteristics and HA approach used to induce adaptations (Figure 11). These analyses indicate that:

- Exercise‐HA lowered resting and end‐exercise HR (5 and 17 beats·min^−1^), exercise metabolic rate (87 mL·min^−1^), resting and end‐exercise *T*_core_ (0.19°C and 0.43°C), and end‐exercise *T*_sk_ (0.54°C).
- Exercise‐HA expanded BV (2.9%), PV (5.6%), and RCV (1.3%), increased WBSR (163 mL·h^−1^), and improved time to exhaustion (48.7%), incremental exercise time (14.2%), and time trial performance (3.1%).
- Extending HA by 1 day further reduced resting *T*_core_ (0.01°C) and end‐exercise *T*_sk_ (0.03°C), and increased Hb_mass_ (1.9 g) and WBSR (9 mL·h^−1^).
- The addition of 15 min to each exposure duration further lowered end‐exercise HR (1 beats·min^−1^), lowered end‐exercise *T*_core_ (0.04°C), and expanded PV (0.4%).
- A 5°C increase in *T*_a_ during HA further lowered end‐exercise HR (2 beats·min^−1^), end‐exercise *T*_core_ (0.05°C), and end‐exercise *T*_sk_ (0.18°C).
- Elevating *P*_a_ by 1 kPa during HA increased RCV (0.7%) and enhanced WBSR (37 mL·h^−1^).
- Controlled hyperthermia HA does not further reduce end‐exercise HR or *T*_core_ compared to constant workrate HA; however, the controlled hyperthermia approach does appear to lower end‐exercise *T*_sk_ to a greater extent and possibly increase WBSR.
- Higher pre‐HA baseline values in resting HR and *T*_core_ are associated with a greater magnitude of change following HA.

## 6 Further Research Considerations

The heterogeneity between studies within the HA literature has previously been identified as a problem when ascertaining the effects of HA protocols (Tyler et al. 2016). To improve the ability to compare HA protocol characteristics and further our understanding of their influence on the adaptive response, it is recommended that:

- More long‐term HA interventions (> 15 exposure days) be undertaken to expand the volume of data regarding the kinetics of adaptation, particularly sweating and hematological adaptations.
- Female representation should be increased across all HA interventions to develop our understanding of potential sex differences in the heat acclimated phenotype.
- Cross‐acclimation studies comparing the physiological and performance responses to dry and humid HA conditions be conducted.
- Studies comparing consecutive to nonconsecutive day HA protocols should be performed, as these apply to elite athlete populations.
- Additional research using different HA approaches (i.e., controlled HR, intermittent exercise) be conducted to more clearly elucidate their impact on the adaptive process.
- Sudomotor adaptations be appropriately contextualized to avoid methodological issues with testing (e.g., short‐duration or intermittent heat response tests, less thermally challenging post‐test) leading to misinterpretation of findings.
- When investigating changes in time trial performance and exercise capacity following HA, consider accounting for factors such as participant fitness level ($\dot{V}$O_2peak_), training history, test protocol and duration, and the number of rest/recovery days following HA before testing.

## Author Contributions

P.M., H.A.B., M.N.S., B.C., and J.D.P. conceptualized and designed the study. P.M. and H.A.B. completed all database searches. P.M., H.A.B., T.H.T., M.K.K., and W.T.J. were involved in the data extraction and assessment of bias. P.M., J.D.P., and A.P.W. analyzed the data and created the publicly available online predictor. P.M., M.N.S., B.C., and J.D.P. were involved in the interpretation of the data. P.M. and J.D.P. drafted the initial manuscript, which was reviewed and edited by all authors. All authors approved the submitted manuscript.

## Ethics Statement

The authors have nothing to report.

## Consent

The authors have nothing to report.

## Conflicts of Interest

The authors declare no conflicts of interest.

## Supporting information


Data S1.

## Data Availability

The data that support the findings of this study are available from the corresponding author upon reasonable request.
