# Supplementary material for: Influence of Exercise Heat Acclimation Protocol Characteristics on Adaptation Kinetics: A Quantitative Review With Bayesian Meta‐Regressions
Source: Compr Physiol. 2025 May 29;15(3):e70017. doi: 10.1002/cph4.70017 (PMC12122934; doi:10.1002/cph4.70017)
Supplement: Supplementary file 1 — Data S1. [file CPH4-15-e70017-s001.zip › Supplementary Materal S2 (figure legends).docx]

**Electronic Supplementary Material S2.**

**Supplementary Figure 1.** Change (Δ) in resting heart rate (HR) for each study included in the model estimate (solid back line), along with the 90% credible interval (dark grey band) and prediction interval (light grey band), plotted across number of exposures (A), exposure duration (B), ambient temperature (T_a_; C), and partial water vapour pressure in air (P_a_; D). Model estimates based on global means when the predictor is not on the x-axis.

**Supplementary Figure 2.** Change (Δ) in end-exercise heart rate (HR) for each study included in the model estimate (solid back line), along with the 90% credible interval (dark grey band) and prediction interval (light grey band), plotted across number of exposures (A), exposure duration (B), ambient temperature (T_a_; C), and partial water vapour pressure in air (P_a_; D). All model estimates utilise global means when the predictor is not on the x-axis.

**Supplementary Figure 3.** Change (Δ) in exercise metabolic rate for each study included in the model estimate (solid back line), along with the 90% credible interval (dark grey band) and prediction interval (light grey band), plotted across number of exposures (A), exposure duration (B), ambient temperature (T_a_; C), and partial water vapour pressure in air (P_a_; D). All model estimates utilise global means when the predictor is not on the x-axis.

**Supplementary Figure 4.** Change (Δ) in blood volume (BV) for each study included in the model estimate (solid back line), along with the 90% credible interval (dark grey band) and prediction interval (light grey band), plotted across number of exposures (A), exposure duration (B), ambient temperature (T_a_; C), and partial water vapour pressure in air (P_a_; D). All model estimates utilise global means when the predictor is not on the x-axis.

**Supplementary Figure 5.** Change (Δ) in plasma volume (PV) for each study included in the model estimate (solid back line), along with the 90% credible interval (dark grey band) and prediction interval (light grey band), plotted across number of exposures (A), exposure duration (B), ambient temperature (T_a_; C), and partial water vapour pressure in air (P_a_; D). All model estimates utilise global means when the predictor is not on the x-axis.

**Supplementary Figure 6.** Change (Δ) in red cell volume (RCV) for each study included in the model estimate (solid back line), along with the 90% credible interval (dark grey band) and prediction interval (light grey band), plotted across number of exposures (A), exposure duration (B), ambient temperature (T_a_; C), and partial water vapour pressure in air (P_a_; D). All model estimates utilise global means when the predictor is not on the x-axis.

**Supplementary Figure 7.** Change (Δ) in plasma hemoglobin mass (Hb_mass_) for each study included in the model estimate (solid back line), along with the 90% credible interval (dark grey band) and prediction interval (light grey band), plotted across number of exposures (A), exposure duration (B), ambient temperature (T_a_; C), and partial water vapour pressure in air (P_a_; D). All model estimates utilise global means when the predictor is not on the x-axis.

**Supplementary Figure 8.** Change (Δ) in resting core temperature (T_core_) for each study included in the model estimate (solid back line), along with the 90% credible interval (dark grey band) and prediction interval (light grey band), plotted across number of exposures (A), exposure duration (B), ambient temperature (T_a_; C), and partial water vapour pressure in air (P_a_; D). All model estimates utilise global means when the predictor is not on the x-axis.

**Supplementary Figure 9.** Change (Δ) in end-exercise core temperature (T_core_) for each study included in the model estimate (solid back line), along with the 90% credible interval (dark grey band) and prediction interval (light grey band), plotted across number of exposures (A), exposure duration (B), ambient temperature (T_a_; C), and partial water vapour pressure in air (P_a_; D). All model estimates utilise global means when the predictor is not on the x-axis.

**Supplementary Figure 10.** Change (Δ) in end-exercise skin temperature (T_sk_) for each study included in the model estimate (solid back line), along with the 90% credible interval (dark grey band) and prediction interval (light grey band), plotted across number of exposures (A), exposure duration (B), ambient temperature (T_a_; C), and partial water vapour pressure in air (P_a_; D). model estimates utilise global means when the predictor is not on the x-axis.

**Supplemenntary Figure 11.** Change (Δ) in whole body sweat rate (WBSR) for each study included in the model estimate (solid back line), along with the 90% credible interval (dark grey band) and prediction interval (light grey band), plotted across number of exposures (A), exposure duration (B), ambient temperature (T_a_; C), and partial water vapour pressure in air (P_a_; D). All model estimates utilise global means when the predictor is not on the x-axis.

**Supplementary Figure 12.** Change (Δ) in upper back local sweat rate (LSR) for each study included in the model estimate (solid back line), along with the 90% credible interval (dark grey band) and prediction interval (light grey band), plotted across number of exposures (A), exposure duration (B), ambient temperature (T_a_; C), and partial water vapour pressure in air (P_a_; D). All model estimates utilise global means when the predictor is not on the x-axis.

**Supplementary Figure 13.** Change (Δ) in forearm local sweat rate (LSR) for each study included in the model estimate (solid back line), along with the 90% credible interval (dark grey band) and prediction interval (light grey band), plotted across number of exposures (A), exposure duration (B), ambient temperature (T_a_; C), and partial water vapour pressure in air (P_a_; D). All model estimates utilise global means when the predictor is not on the x-axis.

**Supplementary Figure 14.** Change (Δ) in sweat sodium ([Na^+^]) for each study included in the model estimate (solid back line), along with the 90% credible interval (dark grey band) and prediction interval (light grey band), plotted across number of exposures (A), exposure duration (B), ambient temperature (T_a_; C), and partial water vapour pressure in air (P_a_; D). All model estimates utilise global means when the predictor is not on the x-axis.

**Supplementary Figure 15.** Change (Δ) in time to exhaustion for each study included in the model estimate (solid back line), along with the 90% credible interval (dark grey band) and prediction interval (light grey band), plotted across number of exposures (A), exposure duration (B), ambient temperature (T_a_; C), and partial water vapour pressure in air (P_a_; D). All model estimates utilise global means when the predictor is not on the x-axis.

**Supplementary Figure 16.** Change (Δ) in incremental exercise time for each study included in the model estimate (solid back line), along with the 90% credible interval (dark grey band) and prediction interval (light grey band), plotted across number of exposures (A), exposure duration (B), ambient temperature (T_a_; C), and partial water vapour pressure in air (P_a_; D). All model estimates utilise global means when the predictor is not on the x-axis.

**Supplementary Figure 17.** Change (Δ) in time trial performance for each study included in the model estimate (solid back line), along with the 90% credible interval (dark grey band) and prediction interval (light grey band), plotted across number of exposures (A), exposure duration (B), ambient temperature (T_a_; C), and partial water vapour pressure in air (P_a_; D). All model estimates utilise global means when the predictor is not on the x-axis.
