# Supplementary material for: Influence of Exercise Heat Acclimation Protocol Characteristics on Adaptation Kinetics: A Quantitative Review With Bayesian Meta‐Regressions
Source: Compr Physiol. 2025 May 29;15(3):e70017. doi: 10.1002/cph4.70017 (PMC12122934; doi:10.1002/cph4.70017)
Supplement: Supplementary file 1 — Data S1. [file CPH4-15-e70017-s001.zip › Supplementary Material S1 (risk of bias) - UPDATED.docx]

**Electronic Supplementary Material Table S1.** Modified McMaster Critical Appraisal Tool

Comments

| **Study concept** | **Question** | **Scoring (awarded points)**  **No = high risk of bias**  **Yes = low risk of bias** |
| --- | --- | --- |
| **Study Purpose:**  Was the purpose stated clearly? | Outline the purpose of the study. How does the study apply to the research question? Was relevant background literature reviewed? | - No (0) - Yes (1) - Unsure (0) |
| **Study Design (1):**  Was there any control within the study design? | Describe the study design. Was the design appropriate for the study question? | - No (0) - Yes (1) - Unsure (0) |
| **Study Design (2):**  Was there any randomization? |  | - No (0) - Yes (1) - Unsure (0) |
| **Sample:**  Was the sample described and the size justified? | Sampling (who; characteristics; how many; how was sampling done?) If more than one group, was there similarity between the groups? Was this sample size justified? | - No (0) - Yes (1) - Unsure (0) |
| **Reliability and Validity:**  Were the outcome measures reliable and valid? | Was the frequency of the outcome measurements identified? | - No (0) - Yes (1) - Unsure (0) |
| **Intervention:**  Intervention was described in detail? | Provide a short description of the intervention (focus, who delivered it, how often, setting). Could the intervention be replicated? | - No (0) - Yes (1) - Unsure (0) |
| **Statistical Analysis:**  Were the  analysis method(s) appropriate? And reported in terms of statistical significance? | What were the results? Were they statistically significant (i.e., p < 0.05)? If not statistically significant, was study big enough to show an important difference if it should occur? If there were multiple outcomes, was that considered for the statistical analysis? | - No (0) - Yes (1) - Unsure (0) |
| **Conclusion:**  Conclusions were appropriate given study methods and results | What did the study conclude? What are the implications of these results? What were the main limitations or biases in the study? | - No (0) - Yes (1) - Unsure (0) |
|  | Total score: | /8 |

Modified version of an extracted file from: Evidence-Based Practice Research Group at McMaster University. Quantitative Review Form. Copyright: Law M, Stewart D, Pollock N, Letts L, Bosch J and Westmorland M, 1998. McMaster University. Available from: http://srs-mcmaster.ca/research/evidence-based-practiceresearch-group/#OlXEXdby
