# Supplementary material for: Influence of Exercise Heat Acclimation Protocol Characteristics on Adaptation Kinetics: A Quantitative Review With Bayesian Meta‐Regressions
Source: Compr Physiol. 2025 May 29;15(3):e70017. doi: 10.1002/cph4.70017 (PMC12122934; doi:10.1002/cph4.70017)
Supplement: Supplementary file 1 — Data S1. [file CPH4-15-e70017-s001.zip › Supplementary Material S3 (table) - Updated.docx]

| **Electronic Supplementary Material S3. Papers with outcome variables included in the meta-analysis** | | | | | | | | | | | | | | | | | |
| --- | --- | --- | --- | --- | --- | --- | --- | --- | --- | --- | --- | --- | --- | --- | --- | --- | --- |
| Reference | Resting heart rate | End-exercise heart rate | Exercise metabolic rate | Blood volume | Plasma volume | Red cell volume | Hemoglobin mass | Resting core temperature | End-exercise core temperature | End exercise skin temperature | Whole-body sweat rate | Upper back local sweat rate | Forearm local sweat rate | Sweat sodium concentration | Time to exhaustion | Incremental exercise time | Time trial Performance |
| Adams *et al.* (198) | N | Y | N | N | N | N | N | N | Y | N | Y | N | N | N | N | N | N |
| Alkemade *et al.* (199) | N | Y | N | N | N | N | N | Y | Y | N | Y | N | N | N | N | N | N |
| Alkemade *et al.* (172) | N | Y | N | N | N | N | N | Y | Y | Y | Y | N | N | N | N | Y | N |
| Amano *et al.* (135) | N | Y | N | N | N | N | N | N | Y | N | Y | N | N | N | N | N | N |
| Amano *et al.* (134) | Y | Y | N | N | N | N | N | Y | Y | Y | Y | Y | N | N | N | N | N |
| Aoyagi *et al.* (72) | Y | Y | N | Y | Y | Y | N | Y | Y | Y | Y | N | N | N | N | N | N |
|  | Y | Y | N | Y | Y | Y | N | Y | Y | Y | Y | N | N | N | N | N | N |
| Aoyagi *et al.* (201) | N | N | N | N | N | N | N | Y | Y | Y | N | N | N | N | N | N | N |
|  | N | N | N | N | N | N | N | Y | Y | Y | N | N | N | N | N | N | N |
| Aoyagi *et al.* (136) | Y | Y | N | Y | Y | N | N | Y | Y | Y | Y | N | N | N | N | N | N |
|  | Y | Y | N | Y | Y | N | N | Y | Y | Y | Y | N | N | N | N | N | N |
| Aoyagi *et al.* (147) | N | N | N | N | N | N | N | N | N | N | Y | N | N | N | N | N | N |
|  | N | N | N | N | N | N | N | N | N | N | Y | N | N | N | N | N | N |
|  | N | N | N | N | N | N | N | N | N | N | Y | N | N | N | N | N | N |
|  | N | N | N | N | N | N | N | N | N | N | Y | N | N | N | N | N | N |
| Amorim *et al.* (200) | N | Y | N | N | N | N | N | Y | Y | N | N | N | N | N | N | N | N |
| Armstrong *et al.* (202) | N | Y | N | N | N | N | N | N | Y | Y | N | N | N | N | N | N | N |
| Armstrong *et al.* (203) | N | Y | N | N | Y | N | N | N | Y | N | Y | N | N | Y | N | N | N |
| Armstrong *et al.* (146) | N | Y | N | N | Y | N | N | N | Y | N | Y | Y | N | N | N | N | N |
| Avellini *et al.* (52) | N | N | N | N | N | N | N | N | N | N | Y | N | N | N | N | N | N |
| Avellini *et al.* (205) | N | Y | N | N | Y | N | N | N | Y | Y | Y | N | N | N | N | N | N |
| Barberio *et al.* (206) | Y | N | N | Y | Y | N | N | Y | N | N | N | N | N | N | N | N | N |
| Best *et al.* (48) | N | Y | N | N | N | N | N | N | Y | Y | Y | N | N | N | N | N | N |
| Brade *et al.* (207) | N | N | N | N | N | N | N | Y | N | N | N | N | N | N | N | N | N |
| Brock *et al.* (137) | N | Y | N | N | Y | N | N | N | Y | N | Y | N | N | N | N | N | N |
| Buono *et al.* (208) | N | Y | N | N | N | N | N | Y | Y | N | N | N | N | N | N | N | N |
| Buono *et al.* (209) | N | N | N | N | N | N | N | N | N | N | Y | N | N | N | N | N | N |
| Buono *et al.* (210) | N | Y | N | N | N | N | N | N | Y | N | Y | N | Y | N | N | N | N |
| Burk *et al.* (164) | Y | Y | N | N | Y | N | N | Y | Y | N | N | N | N | N | Y | N | N |
| Callovini *et al.* (165) | N | N | N | N | N | N | N | N | N | N | N | N | N | N | Y | N | N |
| Campbell *et al.* (211) | N | N | N | N | N | N | N | Y | Y | N | N | N | N | N | N | N | N |
| Casadio *et al.* (212) | N | N | N | N | N | N | N | N | N | N | Y | N | N | N | N | N | N |
| Castle *et al.* (213) | N | Y | N | N | Y | N | N | Y | Y | N | Y | N | N | N | N | N | N |
| Chalmers *et al.* (320) | N | N | N | N | Y | N | N | N | N | N | N | N | N | N | N | N | N |
| Chen *et al.* (215) | N | N | N | N | N | N | N | N | N | N | Y | N | Y | N | N | N | N |
| Cheuvront *et al.* (217) | N | Y | N | N | N | N | N | N | Y | N | Y | N | N | N | N | N | N |
| Chinevere *et al.* (218) | N | Y | N | N | N | N | N | N | Y | N | Y | N | N | N | N | N | N |
| Ciuha *et al.* (219) | Y | N | N | N | N | N | N | Y | N | N | N | N | N | N | N | N | N |
| Cleland *et al.* (220) | N | Y | Y | N | N | N | N | N | Y | Y | N | N | N | N | N | N | N |
| Corbett *et al.* (221) | N | N | N | N | Y | N | N | Y | N | N | Y | N | N | N | N | N | N |
| Curley *et al.* (129) | N | N | N | N | N | N | N | N | Y | N | Y | N | N | N | N | N | N |
| Daanen *et al.* (222) | N | Y | N | N | N | N | N | Y | Y | Y | Y | N | N | N | N | N | N |
| Dawson *et al.* (223) | N | Y | N | Y | Y | Y | N | Y | Y | Y | Y | N | N | N | N | N | N |
| Dileo *et al.* (224) | Y | Y | N | N | N | N | N | Y | Y | Y | Y | N | N | N | N | N | N |
| Dini *et al.* (225) | N | Y | N | N | N | N | N | N | Y | N | N | N | N | N | N | N | N |
| Duvnjak-Zaknich *et al.* (21) | Y | N | N | N | N | N | N | Y | N | N | N | N | N | N | N | N | N |
|  | Y | N | N | N | N | N | N | Y | N | N | N | N | N | N | N | N | N |
| Epstein *et al.* (226) | N | Y | N | N | N | N | N | N | Y | N | N | N | N | N | N | N | N |
| Febbraio *et al.* (94) | N | N | Y | N | N | N | N | Y | Y | N | N | N | N | N | N | N | N |
| Fein *et al.* (20) | N | Y | N | N | N | N | N | N | Y | N | Y | N | N | N | N | N | N |
|  | N | Y | N | N | N | N | N | N | Y | N | Y | N | N | N | N | N | N |
| Finberg *et al.* (79) | N | Y | N | N | N | N | N | N | Y | Y | N | N | N | N | N | N | N |
| Flouris *et al.* (191) | Y | Y | N | N | N | N | N | Y | Y | N | N | N | N | N | N | N | N |
| Fortney *et al.* (227) | N | Y | N | N | Y | Y | N | N | Y | N | Y | N | N | N | N | N | N |
| Francesconi *et al.* (229) | N | Y | N | N | N | N | N | N | Y | N | Y | N | N | N | N | N | N |
| Francesconi *et al.* (230) | N | Y | N | N | N | N | N | N | Y | N | Y | N | N | N | N | N | N |
| Francesconi *et al.* (228) | N | Y | N | N | N | N | N | N | Y | N | Y | N | N | N | N | N | N |
| Frank *et al.* (138) | Y | Y | N | N | N | N | N | Y | N | N | Y | N | N | N | N | N | N |
| Frye *et al.* (231) | N | Y | N | N | N | N | N | N | Y | Y | N | N | N | N | N | N | N |
|  | N | Y | N | N | N | N | N | N | Y | Y | N | N | N | N | N | N | N |
| Fujii *et al.* (232) | Y | Y | Y | N | Y | N | N | Y | Y | Y | N | N | N | N | N | N | N |
| Gale *et al.* (166) | N | N | N | N | N | N | N | N | N | N | N | N | N | N | N | Y | N |
| Garrett *et al.* (177) | Y | Y | N | Y | Y | N | N | Y | Y | N | N | N | N | N | N | N | Y |
| Garrett *et al.* (193) | Y | N | N | N | Y | N | N | N | N | N | N | N | N | N | N | N | N |
| Garrett *et al.* (173) | N | Y | N | N | Y | Y | N | Y | Y | N | N | N | N | N | N | Y | N |
| Garrett *et al.* (192) | N | Y | N | N | Y | Y | N | N | Y | Y | N | N | Y | N | N | Y | N |
|  | N | Y | N | N | Y | Y | N | N | Y | Y | N | N | Y | N | N | Y | N |
| Gerrett *et al.* (174) | N | Y | N | N | N | N | N | Y | Y | Y | Y | N | N | N | N | Y | N |
|  | N | Y | N | N | N | N | N | Y | Y | Y | Y | N | N | N | N | Y | N |
|  | N | N | N | N | N | N | N | Y | Y | N | Y | N | N | N | N | Y | N |
|  | N | N | N | N | N | N | N | Y | Y | N | Y | N | N | N | N | Y | N |
| Gibson *et al.* (126) | Y | N | N | N | N | N | N | Y | N | N | Y | N | N | N | N | N | N |
|  | Y | N | N | N | N | N | N | Y | N | N | Y | N | N | N | N | N | N |
|  | Y | N | N | N | N | N | N | Y | N | N | Y | N | N | N | N | N | N |
| Gibson *et al.* (73) | Y | N | N | Y | Y | N | N | Y | N | N | N | N | N | N | N | N | N |
| Gibson *et al.* (233) | Y | Y | N | N | N | N | N | Y | N | N | N | N | N | N | N | N | N |
|  | Y | N | N | N | N | N | N | Y | N | N | N | N | N | N | N | N | N |
|  | Y | N | N | N | N | N | N | Y | N | N | N | N | N | N | N | N | N |
| Greenleaf *et al.* (234) | N | N | N | Y | Y | Y | N | N | N | N | Y | N | N | N | N | N | N |
| Greenleaf *et al.* (235) | N | N | N | Y | Y | Y | N | N | N | N | N | N | N | N | N | N | N |
|  | N | N | N | Y | Y | Y | N | N | N | N | N | N | N | N | N | N | N |
| Greenleaf *et al.* (236) | N | Y | N | N | Y | N | N | N | Y | N | Y | N | N | N | N | N | N |
| Griefahn (25) | N | N | N | N | N | N | N | N | N | N | Y | N | N | N | N | N | N |
|  | N | N | N | N | N | N | N | N | N | N | Y | N | N | N | N | N | N |
| Guy *et al.* (178) | N | N | N | N | N | N | N | N | Y | N | N | N | N | N | N | N | Y |
| Hahn *et al.* (139) | N | N | N | N | N | N | N | N | N | N | Y | N | N | N | N | N | N |
| Hanson *et al.* (237) | N | N | N | N | Y | N | N | N | N | N | N | N | N | N | N | N | N |
| Haroutounian *et al.* (238) | N | Y | N | N | N | N | N | N | Y | N | Y | N | N | N | N | N | N |
|  | N | Y | N | N | N | N | N | N | Y | N | Y | N | N | N | N | N | N |
| Heled *et al.* (239) | N | N | N | N | N | N | N | N | Y | N | N | N | N | N | N | N | N |
| Hodge *et al.* (240) | N | Y | N | N | N | N | N | N | Y | N | N | N | N | N | N | N | N |
| Hom *et al.* (241) | N | Y | N | N | N | N | N | N | Y | N | Y | N | N | N | N | N | N |
| Horstman *et al.* (176) | N | Y | N | N | N | N | N | N | Y | N | Y | N | N | N | N | N | N |
|  | N | Y | N | N | N | N | N | N | Y | N | Y | N | N | N | N | N | N |
| Houmard *et al.* (29) | N | Y | Y | N | N | N | N | N | Y | N | N | N | N | N | N | N | N |
|  | N | Y | Y | N | N | N | N | N | Y | N | N | N | N | N | N | N | N |
| Inoue *et al.* (242) | N | N | N | N | N | N | N | N | Y | Y | Y | Y | Y | Y | N | N | N |
| James *et al.* (321) | N | N | N | N | N | N | N | N | N | N | Y | N | N | N | N | N | N |
| James *et al.* (87) | Y | Y | N | N | Y | N | N | Y | Y | N | Y | N | N | N | N | N | Y |
| Kaldur *et al.* (243) | Y | N | N | N | N | N | N | Y | N | N | N | N | N | N | N | N | N |
| Kaldur *et al.* (167) | N | N | N | N | N | N | N | N | N | N | N | N | N | N | Y | N | N |
| Kaufman *et al.* (159) | N | Y | N | N | N | N | N | N | Y | Y | Y | N | N | Y | N | N | N |
| Keiser *et al.* (12) | N | N | N | Y | Y | Y | N | N | N | N | Y | N | N | Y | N | N | Y |
| Kelly *et al.* (140) | N | Y | N | N | Y | N | N | Y | Y | N | Y | N | N | N | N | N | N |
| King *et al.* (95) | N | N | Y | N | Y | N | N | N | N | N | N | N | N | N | N | N | N |
| Kirby *et al.* (244) | N | Y | N | N | Y | N | N | N | Y | N | Y | N | N | Y | N | N | N |
| Kirby *et al.* (245) | N | N | N | N | N | N | N | N | N | N | N | N | N | N | N | N | Y |
| Kirwan *et al.* (91) | N | Y | N | N | Y | N | N | N | N | N | N | N | N | N | N | N | N |
| Kissling *et al.* (246) | N | N | N | N | Y | N | N | N | N | N | N | N | N | N | N | N | N |
| Klous *et al.* (247) | N | N | N | N | N | N | N | N | N | N | N | Y | Y | Y | N | N | N |
|  | N | N | N | N | N | N | N | N | N | N | N | Y | Y | Y | N | N | N |
| Klous *et al.* (248) | N | N | N | N | N | N | N | Y | N | N | N | Y | Y | Y | N | N | N |
|  | N | N | N | N | N | N | N | Y | N | N | N | Y | Y | Y | N | N | N |
| Kotze *et al.* (77) | Y | Y | N | N | Y | N | N | Y | Y | N | Y | N | N | N | N | N | N |
| Kristal-Boneh *et al.* (249) | N | N | N | N | Y | N | N | N | N | N | N | N | N | N | N | N | N |
| Kuennen *et al.* (250) | N | Y | Y | N | Y | N | N | N | Y | Y | N | N | N | N | N | N | N |
| Lee *et al.* (130) | N | N | N | N | Y | N | N | N | Y | N | Y | N | N | N | N | N | N |
| Lee *et al.* (85) | Y | N | N | N | Y | N | N | Y | N | N | Y | N | N | N | N | N | N |
| Loeppky (251) | N | N | N | Y | Y | Y | Y | N | N | N | N | N | N | N | N | N | N |
| Lorenzo *et al.* (11) | N | Y | N | N | Y | N | N | N | Y | N | Y | N | N | N | N | N | N |
| Lorenzo *et al.* (252) | N | Y | N | N | Y | N | N | N | Y | N | Y | N | N | N | N | N | Y |
| Lundby *et al.* (50) | N | N | N | Y | Y | Y | Y | Y | Y | N | Y | N | N | N | N | N | Y |
| Lynch *et al.* (156) | Y | Y | N | N | N | N | N | Y | N | N | Y | Y | Y | N | N | N | N |
| Magalhães *et al.* (84) | Y | N | N | N | Y | N | N | Y | N | N | Y | N | N | N | N | N | N |
| Mang *et al.* (253) | N | Y | N | N |  | N | N | Y | Y | N | N | N | N | N | N | N | N |
| Matias *et al.* (254) | Y | N | N | N | N | N | N | N | N | N | N | N | N | N | N | N | N |
| McCleave *et al.* (255) | N | N | N | Y | Y | N | Y | N | N | N | N | N | N | N | N | N | Y |
| McCleave *et al.* (160) | N | N | N | N | Y | N | N | N | Y | N | Y | N | N | Y | N | N | N |
| McCleave *et al.* (256) | N | N | N | N | Y | N | N | N | N | N | N | N | N | N | N | N | N |
| McClung *et al.* (257) | N | Y | N | N | N | N | N | N | Y | N | Y | N | N | N | N | N | N |
| McGlynn *et al.* (148) | N | N | N | N | N | N | N | N | Y | N | Y | N | N | N | N | N | N |
| McIntyre *et al.* (258) | N | Y | Y | Y | Y | N | Y | Y | Y | Y | Y | N | N | N | Y | N | N |
| McIntyre *et al.* (168) | N | N | N | N | Y | N | N | N | N | N | N | N | N | N | N | N | N |
| McLellan *et al.* (259) | N | Y | N | N | N | N | N | N | N | N | N | N | N | N | N | N | N |
| Mee *et al.* (260) | Y | N | N | N | N | N | N | Y | Y | N | Y | N | N | N | N | N | N |
|  | Y | N | N | N | N | N | N | Y | Y | N | Y | N | N | N | N | N | N |
| Mee *et al.* (261) | N | N | N | N | N | N | N | Y | N | N | N | N | N | N | N | N | N |
|  | N | N | N | N | N | N | N | Y | N | N | N | N | N | N | N | N | N |
| Mikkelsen *et al.* (262) | N | N | N | N | N | N | N | N | N | N | N | N | N | N | Y | N | N |
| Mitchell *et al.* (263) | N | Y | N | N | N | N | N | Y | Y | Y | Y | N | N | N | N | N | N |
| Mitchell *et al.* (264) | N | Y | N | N | N | N | N | N | Y | N | Y | N | N | N | N | N | N |
| Molloy *et al.* (265) | N | N | N | N | Y | N | N | N | Y | N | N | N | N | N | N | N | N |
|  | N | N | N | N | Y | N | N | N | Y | N | N | N | N | N | N | N | N |
| Mornas *et al.* (266) | N | Y | N | N | N | N | N | N | Y | Y | Y | N | N | N | N | N | N |
| Moss *et al.* (83) | Y | Y | N | N | N | N | N | Y | Y | Y | Y | N | N | N | N | N | N |
| Naito *et al.* (267) | N | N | N | N | N | N | N | Y | N | N | N | N | N | N | N | N | N |
| Neal *et al.* (162) | N | N | N | Y | Y | N | N | Y | N | N | Y | Y | N | Y | N | N | N |
| Neal *et al.* (161) | Y | Y | N | Y | Y | N | N | Y | Y | Y | Y | Y | N | Y | N | N | N |
|  | Y | Y | N | Y | Y | N | N | Y | Y | Y | Y | Y | N | Y | N | N | N |
| Neufer *et al.* (195) | N | Y | N | N | N | N | N | Y | Y | N | N | N | N | N | N | N | N |
| Nielsen *et al.* (27) | N | N | N | N | Y | N | N | N | N | N | Y | N | N | N | Y | N | N |
| Nielsen *et al.* (26) | N | Y | Y | N | Y | N | N | Y | Y | Y | Y | N | N | N | Y | N | N |
| Notley *et al.* (268) | N | N | N | N | N | N | N | Y | N | N | N | N | N | N | N | N | N |
| Oberholzer *et al.* (111) | N | N | N | Y | Y | Y | Y | N | N | N | N | N | N | N | N | N | N |
| Oöpik *et al.* (169) | N | N | N | N | N | N | N | Y | Y | N | Y | N | N | N | Y | N | N |
| Osborne *et al.* (71) | Y | N | N | N | Y | N | N | Y | N | N | Y | N | N | N | N | N | Y |
| Pandolf *et al.* (78) | N | Y | N | N | N | N | N | N | Y | Y | Y | N | N | N | N | N | N |
| Pandolf *et al.* (81) | N | N | N | N | Y | N | N | N | N | N | Y | N | N | N | N | N | N |
|  | N | N | N | N | N | N | N | N | N | N | Y | N | N | N | N | N | N |
| Parsons *et al.* (269) | N | N | N | N | Y | N | N | N | N | N | N | N | N | N | N | N | N |
| Patterson *et al.* (125) | Y | N | N | N | N | N | N | Y | N | N | Y | N | N | N | N | N | N |
| Patterson *et al.* (270) | N | N | N | N | Y | Y | N | N | N | N | N | N | N | N | N | N | N |
| Patterson *et al.* (154) | N | N | N | N | Y | N | N | N | N | N | Y | N | N | Y | N | N | N |
| Périard *et al.* (271) | Y | N | N | N | N | N | N | N | N | N | N | N | N | N | N | N | N |
| Périard *et al.* (272) | N | N | N | Y | Y | Y | Y | N | Y | N | N | N | N | N | N | N | N |
| Périard *et al.* (273) | Y | N | N | N | N | N | Y | N | N | N | N | N | N | N | N | N | Y |
| Petersen *et al.* (141) | Y | N | N | N | N | N | N | Y | Y | Y | Y | N | N | N | N | N | N |
| Pethick *et al.* (274) | N | N | N | N | Y | N | Y | Y | N | N | N | N | N | N | N | N | Y |
|  | N | N | N | N | Y | N | Y | Y | N | N | N | N | N | N | N | N | Y |
| Philp *et al.* (275) | N | N | N | N | Y | N | N | N | N | N | N | N | N | N | N | N | N |
| Philp *et al.* (276) | N | N | Y | N | Y | N | N | N | N | N | Y | N | N | N | N | N | N |
| Pichan *et al.* (51) | N | N | N | N | N | N | N | N | N | N | N | N | N | Y | N | N | N |
| Piil *et al.* (277) | Y | N | N | N | N | N | N | Y | N | N | N | N | N | N | N | N | N |
| Poh *et al.* (278) | N | Y | N | N | N | N | N | N | Y | N | N | N | N | N | N | N | N |
| Poirier *et al.* (196) | N | N | N | N | N | N | N | N | N | N | Y | Y | Y | N | N | N | N |
| Poirier *et al.* (128) | N | Y | N | N | N | N | N | N | Y | Y | N | N | N | N | N | N | N |
| Pryor *et al.* (279) | Y | N | N | N | N | N | N | Y | Y | Y | Y | N | N | N | N | N | N |
| Pryor *et al.* (280) | N | Y | N | N | N | N | N | N | Y | Y | Y | N | N | N | N | N | N |
| Pryor *et al.* (281) | N | N | N | N | N | N | N | N | N | N | Y | N | N | N | N | N | N |
| Racinais *et al.* (282) | N | N | N | N | N | N | N | N | Y | N | Y | N | N | N | N | N | N |
| Racinais *et al.* (155) | N | N | N | Y | Y | Y | N | Y | N | N | Y | N | N | N | N | N | N |
| Ravanelli *et al.* (283) | Y | Y | N | N | N | N | N | Y | Y | Y | N | Y | Y | N | N | N | N |
| Ravanelli *et al.* (284) | N | N | N | N | N | N | N | Y | Y | Y | Y | Y | Y | N | N | N | N |
| Reeve *et al.* (142) | Y | N | N | N | N | N | N | Y | N | N | Y | N | N | N | Y | N | N |
| Regan *et al.* (285) | N | N | N | N | N | N | N | Y | Y | N | N | N | Y | N | N | N | N |
| Relf *et al.* (286) | Y | N | N | N | N | N | N | Y | N | N | Y | Y | Y | N | N | N | N |
| Rendell *et al.* (82) | Y | Y | Y | Y | N | N | Y | Y | Y | Y | Y | Y | N | Y | N | N | N |
| Rivas *et al.* (143) | N | N | N | N | N | N | N | N | Y | Y | Y | N | N | N | N | N | N |
| Rønnestad *et al.* (110) | N | N | N | Y | Y | Y | Y | N | N | N | N | N | N | N | N | N | N |
| Rønnestad *et al.* (109) | N | N | N | Y | Y | Y | Y | N | N | N | N | N | N | N | N | N | N |
| Roussey *et al.* (131) | N | N | N | N | Y | N | N | N | N | N | Y | N | N | N | N | N | Y |
|  | N | N | N | N | Y | N | N | N | N | N | Y | N | N | N | N | N | Y |
| Rowell *et al.* (322) | N | Y | N | N | N | N | N | N | Y | Y | Y | N | N | N | N | N | N |
| Saillant *et al.* (287) | N | Y | N | N | Y | N | N | N | Y | N | Y | N | N | N | N | N | N |
| Salgado *et al.* (288) | N | Y | N | N | Y | N | N | N | Y | N | Y | N | N | N | N | N | N |
| Sawka *et al.* (289) | N | Y | N | N | N | N | N | N | Y | N | N | N | N | N | N | N | N |
| Schleh *et al.* (290) | N | Y | N | N | Y | N | N | N | Y | Y | Y | N | N | N | N | N | N |
|  | N | Y | N | N | Y | N | N | N | Y | Y | Y | N | N | N | N | N | N |
| Schmit *et al.* (291) | N | N | N | N | N | N | N | Y | N | N | Y | N | N | N | N | N | Y |
|  | N | N | N | N | N | N | N | Y | N | N | Y | N | N | N | N | N | Y |
| Sekiguchi *et al.* (292) | N | N | N | N | N | N | N | N | N | N | Y | N | N | N | N | N | N |
| Senay *et al.* (90) | N | N | N | N | Y | N | N | N | N | N | N | N | N | N | N | N | N |
| Senay (293) | N | N | N | N | N | N | N | N | Y | N | N | N | N | N | N | N | N |
| Shapiro *et al.* (294) | N | Y | N | Y | N | N | N | N | Y | N | Y | N | N | N | N | N | N |
| Shapiro *et al.* (76) | N | Y | N | N | N | N | N | N | Y | N | Y | N | N | N | N | N | N |
|  | N | Y | N | N | N | N | N | N | Y | N | Y | N | N | N | N | N | N |
| Shaw *et al.* (171) | N | N | N | N | N | N | N | N | N | Y | N | N | N | N | N | Y | N |
| Shvartz *et al.* (24) | N | N | Y | N | N | N | N | N | N | N | Y | N | N | N | N | N | N |
| Shvartz *et al.* (80) | Y | Y | Y | N | N | N | N | Y | Y | N | Y | N | N | N | N | N | N |
|  | Y | Y | Y | N | N | N | N | Y | Y | N | Y | N | N | N | N | N | N |
|  | Y | Y | Y | N | N | N | N | Y | Y | N | Y | N | N | N | N | N | N |
| Shvartz *et al.* (295) | Y | Y | N | N | N | N | N | N | Y | N | Y | N | N | N | N | N | N |
| Shvartz *et al.* (62) | N | N | N | N | N | N | N | N | N | N | N | N | N | N | N | N | N |
| Smith *et al.* (157) | Y | N | N | N | N | N | N | Y | N | N | N | N | N | N | N | N | N |
| Sotiridis *et al.* (296) | N | N | Y | N | Y | N | N | Y | N | N | N | N | N | N | N | N | N |
| Strydom *et al.* (298) | N | N | N | N | N | N | N | N | Y | N | N | N | N | N | N | N | N |
| Stearns *et al.* (297) | N | Y | N | N | N | N | N | N | Y | N | N | N | N | N | N | N | N |
| Sumi *et al.* (299) | N | Y | N | N | Y | N | N | Y | Y | N | Y | N | N | N | N | N | N |
| Sumi *et al.* (300) | N | Y | N | N | Y | N | N | Y | Y | N | Y | N | N | N | N | N | N |
| Sunderland *et al.* (301) | N | N | N | N | Y | N | N | N | N | N | N | N | N | N | N | N | N |
| Takamata *et al.* (302) | N | N | N | Y | Y | N | N | N | N | N | N | N | N | N | N | N | N |
| Takeno *et al.* (144) | N | Y | N | Y | Y | Y | N | N | Y | Y | Y | N | N | N | N | N | N |
| Tamm *et al.* (170) | Y | Y | N | N | N | N | N | Y | Y | N | N | N | N | N | Y | N | N |
| Tebeck *et al.* (28) | N | N | N | N | Y | N | N | N | N | N | Y | N | N | N | N | N | N |
|  | N | N | N | N | Y | N | N | N | N | N | Y | N | N | N | N | N | N |
| Travers *et al.* (102) | Y | Y | N | Y | Y | Y | Y | N | Y | Y | N | N | N | N | N | N | N |
| Travers *et al.* (74) | Y | Y | N | Y | N | N | N | Y | Y | Y | Y | N | N | N | N | N | N |
|  | Y | Y | N | Y | N | N | N | Y | Y | Y | Y | N | N | N | N | N | N |
| Travers *et al.* (86) | Y | N | N | Y | Y | Y | N | Y | N | N | Y | N | N | N | N | N | Y |
|  | Y | N | N | Y | Y | Y | N | Y | N | N | Y | N | N | N | N | N | Y |
| Chen *et al.* (216) | N | N | N | N | N | N | N | N | N | N | Y | N | N | N | N | N | N |
| Waldock *et al.* (149) | Y | N | N | N | Y | N | N | Y | N | N | Y | N | N | N | N | N | N |
| Waldron *et al.* (303) | N | Y | N | N | Y | N | N | N | Y | N | N | N | N | N | N | N | N |
| Waldron *et al.* (304) | Y | N | N | N | N | N | N | Y | N | N | N | N | N | N | N | N | N |
| Watkins *et al.* (305) | N | Y | Y | N | Y | N | N | Y | Y | Y | Y | N | N | N | N | N | N |
| Wallett *et al.* (323) | Y | N | N | N | N | N | N | Y | N | N | Y | N | N | N | N | N | N |
| Weller *et al.* (307) | N | Y | N | N | N | N | N | Y | Y | Y | Y | N | N | N | N | N | N |
|  | N | Y | N | N | N | N | N | Y | Y | Y | Y | N | N | N | N | N | N |
| White *et al.* (308) | N | Y | N | N | Y | N | N | N | Y | Y | Y | N | N | N | N | N | N |
| Willmott *et al.* (145) | Y | Y | N | N | N | N | N | Y | Y | N | Y | N | N | N | N | N | Y |
|  | N | N | N | N | N | N | N | N | N | N | Y | N | N | N | N | N | Y |
| Willmott *et al.* (309) | Y | N | N | N | Y | N | N | Y | N | N | N | N | N | N | N | N | N |
| Wingfield *et al.* (310) | Y | Y | N | N | N | N | N | N | N | Y | N | N | N | N | N | N | Y |
|  | Y | Y | N | N | N | N | N | N | N | Y | N | N | N | N | N | N | Y |
| Wood *et al.* (311) | N | Y | N | N | N | N | N | N | Y | N | N | N | N | N | N | N | N |
|  | N | Y | N | N | N | N | N | N | Y | N | N | N | N | N | N | N | N |
| Wyndham *et al.* (108) | N | N | N | N | Y | N | N | N | N | N | N | N | N | N | N | N | N |
| Wyndham *et al.* (312) | N | Y | N | N | N | N | N | N | Y | Y | N | N | N | N | N | N | N |
| Yamada *et al.* (313) | N | N | N | N | Y | N | N | N | N | N | N | N | N | N | N | N | N |
| Yamazaki (314) | N | Y | N | N | N | N | N | Y | N | N | N | N | N | N | N | N | N |
| Yamazaki *et al.* (315) | Y | Y | N | N | N | N | N | Y | N | N | N | N | N | N | N | N | N |
| Young *et al.* (316) | N | Y | Y | N | N | N | N | N | Y | N | Y | N | N | N | N | N | N |
| Zappe *et al.* (317) | N | N | N | N | Y | N | N | N | N | N | N | N | N | N | N | N | N |
| Zhang *et al.* (318) | N | N | N | N | N | N | N | N | N | Y | N | N | N | N | N | N | N |
| Zimmermann *et al.* (319) | N | N | N | N | N | N | N | Y | N | N | Y | N | N | N | N | N | Y |

Y denotes yes; N denotes no.
